# The YNP Metagenome Project: Environmental Parameters Responsible for Microbial Distribution in the Yellowstone Geothermal Ecosystem

**DOI:** 10.3389/fmicb.2013.00067

**Published:** 2013-05-06

**Authors:** William P. Inskeep, Zackary J. Jay, Susannah G. Tringe, Markus J. Herrgård, Douglas B. Rusch, YNP Metagenome Project Steering Committee and Working Group Members

**Affiliations:** ^1^Department of Land Resources and Environmental Sciences, Montana State UniversityBozeman MT, USA; ^2^Thermal Biology Institute, Montana State UniversityBozeman MT, USA; ^3^Department of Energy, Joint Genome InstituteWalnut Creek, CA, USA; ^4^Novo Nordisk Foundation Center for Biosustainability, Technical University of DenmarkHørsholm Denmark; ^5^Center for Genomics and Bioinformatics, Indiana UniversityBloomington, IN, USA

**Keywords:** thermophiles, geochemistry, microbial interactions, microbial mats, functional genomics

## Abstract

The Yellowstone geothermal complex contains over 10,000 diverse geothermal features that host numerous phylogenetically deeply rooted and poorly understood archaea, bacteria, and viruses. Microbial communities in high-temperature environments are generally less diverse than soil, marine, sediment, or lake habitats and therefore offer a tremendous opportunity for studying the structure and function of different model microbial communities using environmental metagenomics. One of the broader goals of this study was to establish linkages among microbial distribution, metabolic potential, and environmental variables. Twenty geochemically distinct geothermal ecosystems representing a broad spectrum of Yellowstone hot-spring environments were used for metagenomic and geochemical analysis and included approximately equal numbers of: (1) phototrophic mats, (2) “filamentous streamer” communities, and (3) archaeal-dominated sediments. The metagenomes were analyzed using a suite of complementary and integrative bioinformatic tools, including phylogenetic and functional analysis of both individual sequence reads and assemblies of predominant phylotypes. This volume identifies major environmental determinants of a large number of thermophilic microbial lineages, many of which have not been fully described in the literature nor previously cultivated to enable functional and genomic analyses. Moreover, protein family abundance comparisons and in-depth analyses of specific genes and metabolic pathways relevant to these hot-spring environments reveal hallmark signatures of metabolic capabilities that parallel the distribution of phylotypes across specific types of geochemical environments.

## Introduction

The Yellowstone hotspot is responsible for an enormous number (>14,000) and diversity of thermal features that cover a wide range in pH (2–10), temperature (40–92°C), and geochemical properties (Fournier, 1989; Rye and Truesdell, 2007). The waters, rocks, and mineral surfaces in these geothermal sites provide an assortment of electron donors such as hydrogen, sulfide, and ferrous iron, as well as electron acceptors (e.g., dissolved oxygen or elemental S) that are vital to the survival of thermophilic microorganisms (Brock, 1978). Remarkably, many sites exhibit relative stability or regular patterns of temporal change, despite the dynamic nature of geothermal activity (Ball et al., 1998, 2002, 2010; McCleskey et al., 2005). Microbial communities in high-temperature environments are often dominated by just several types of microorganisms (phylotypes), and in general are significantly less diverse than lower-temperature habitats. Geothermal systems thus serve as valuable models for establishing linkages between genomic potential and environmental parameters, and for understanding environmental controls on community structure and function. Moreover, many current thermal environments in YNP harbor modern-day analogs of microbial communities potentially important in ancient Earth, perhaps most notably, cyanobacterial mats that may have analogs dating back to the beginning of oxygenic photosynthesis and the “Great Oxidation Event” (Canfield, 2005; Konhauser, 2006, 2009; Sessions et al., 2009). Consequently, the expansive field site of YNP provides a natural laboratory for studying microbial evolution and adaptation across uniquely protected and preserved thermal environments.

The evolutionary history of all three branches of Life (*Bacteria*, *Archaea*, and *Eucarya*) is intertwined with major geological events that have shaped the planet (Nealson and Ghiorse, 2001; Konhauser, 2006). Relationships between geological events and microbial function are subjects of considerable debate and interest across numerous disciplines, and provide a strong basis for interdisciplinary collaboration. The extant global distribution of microorganisms reveals an astounding diversity of functional attributes, many of which are specialized to specific physical or chemical constraints. The importance of microbially mediated reactions in the geosciences has been discussed (Newman and Banfield, 2002; Reysenbach and Shock, 2002; Falkowski et al., 2008), and numerous metabolic pathways are directly involved in key global processes such as nutrient cycling (e.g., C, N, S, Fe), gas exchange (CO_2_ and CH_4_), and trace element cycling (e.g., As, Sb, Hg). However, the microbial complexity typical of temperate soil and aquatic habitats (e.g., Madigan et al., 2012) has contributed to the difficulty in understanding specific linkages between community members and material cycling (Tringe et al., 2005; DeLong et al., 2006; Rusch et al., 2007). Metagenomics of high-temperature communities in YNP provides an unparalled opportunity to study specific microbiological associations across widely different geochemistry and mineralogy under highly controlled, pseudo-steady-state conditions.

**Figure 1:**
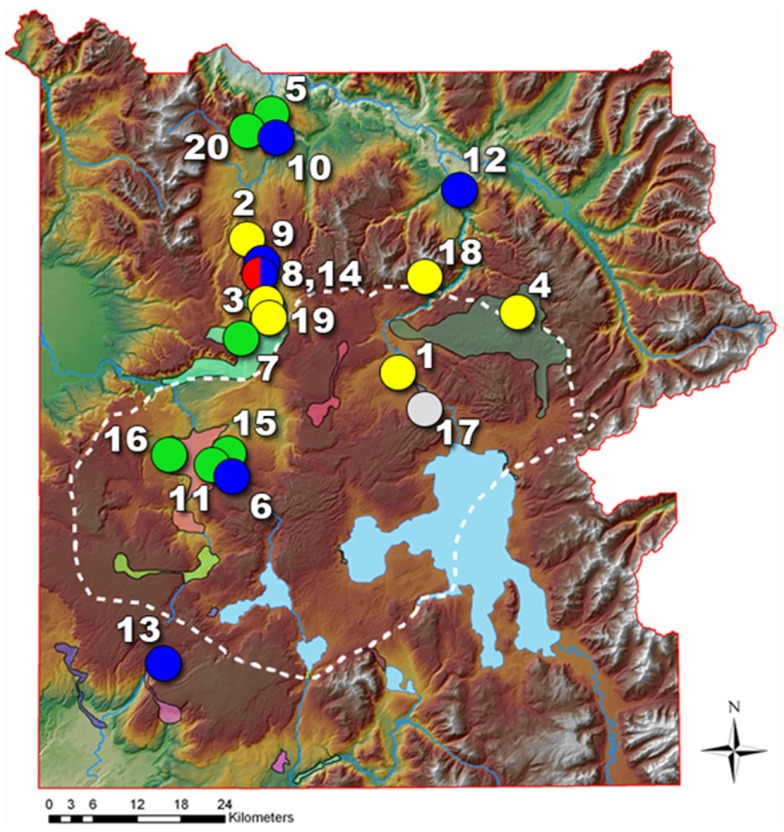
**Map of Yellowstone National Park (YNP) showing the locations of 20 geothermal sites sampled for metagenome and geochemical analysis (green = phototrophic sites; yellow/red = archaeal sites; blue = Aquificales “streamer” communities; gray = Obsidian Pool Prime; site numbers defined in Table 1)**. The dashed line represents the boundary of the most recent caldera.

Metagenome sequencing of environmental DNA has provided a useful tool for studying microbial community structure and function in numerous environments (e.g., Tyson et al., 2004; Tringe et al., 2005; Baker et al., 2006; DeLong et al., 2006; Rusch et al., 2007; Dick et al., 2009). Prior metagenome sequencing in geothermal systems of YNP has been used to dissect phototrophic mat communities (Klatt et al., 2011), and establish linkages among geochemical processes and microbial populations of chemotrophic communities (Inskeep et al., 2010). These studies provide specific examples of what can be gained from detailed phylogenetic and functional analysis of metagenome sequence. Here we describe, characterize and compare metagenomes and associated metadata collected across 20 different geothermal sites (Figure 1), which were selected to cover some of the major types of geochemical and microbiological systems active in YNP. The specific objectives of this manuscript were to (i) discuss rationale for the study design that focused on specific linkages between environmental parameters and microbial community structure, (ii) demonstrate the primary geochemical and geophysical attributes that separate different high-temperature microbial habitats, and (iii) provide an overview of metagenome sequence content and high-level (TIGRFAM) functional analyses that serve to introduce subsequent studies focused on three main geobiological ecosystem types: (1) phototrophic mats; (2) *Aquificales*-rich “filamentous- streamer” communities; and (3) archaeal-dominated sediments. These studies are the outgrowth of one of the major collaborative activities of an NSF Research Coordination Network, established to promote and coordinate research focused on geothermal biology and geochemistry in YNP. Importantly, the research presented here represents the first comprehensive and coordinated non-PCR based survey of microorganisms distributed across a broad spectrum of high-temperature habitats in YNP.

## Results and Discussion

### Geochemistry and environmental context

**Figure 2:**
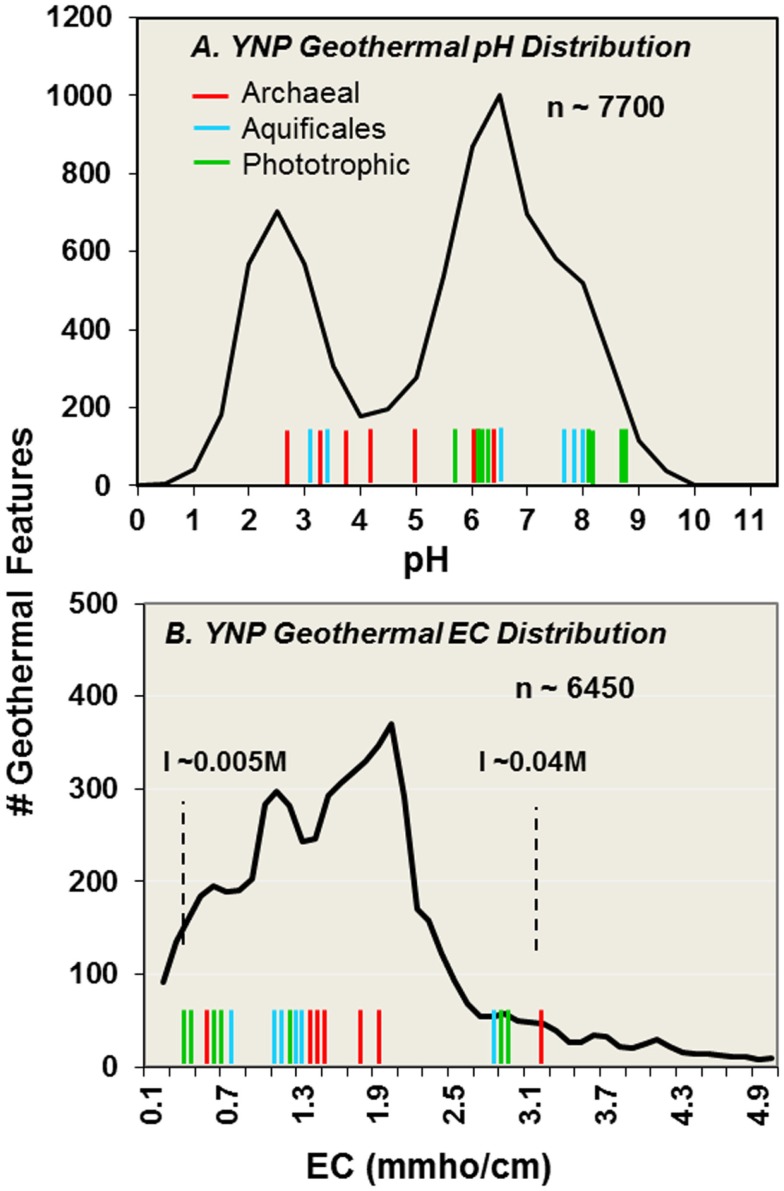
**Distribution of pH (A) and electrical conductivity (EC) (B) values across 7700 and 6450 geothermal features, respectively, cataloged in the Yellowstone Center for Resources (YCR) database (available on line via www.rcn.montana.edu)**. Vertical bars along pH and EC axes indicate values of the 20 sites described in the current study (Aquificales “streamer” communities = blue; archaeal-dominated sites = red; phototrophic mats = green). Ionic strengths ranging from ∼0.005–0.04 M are indicated with dashed lines (WS_18 has the highest EC in the study of ∼3.2 mmho/cm).

A geo-referenced geothermal database developed by the Yellowstone Center for Resources (U.S. National Park Service) contains information for numerous geothermal features including pH, temperature, electrical conductivity (EC), and site context (with image cataloging). A version of this database (∼8000 entries) is available at the RCN YNP website^1^, as well as other data on geothermal sites collected by either the U.S. Geological Survey (e.g., Ball et al., 1998, 2002, 2010; McCleskey et al., 2005) or individual researchers. The distribution of pH values across geothermal features in YNP is bimodal with peaks near pH 2.5 and 6.5 (Figure 2, based on *n* ∼ 7700). While sites chosen for this study (colored bars along *x*-axis) strategically cover the bimodal pH ranges observed in YNP (Figure 2), the microbial communities inhabiting this wide range in pH are also strongly influenced by temperature, oxygen, sulfide, and other physical variables. EC values for a similar number of sites (*n* ∼ 6450) show that most geothermal systems in YNP exhibit EC values ranging from 0.1–3 dS/m (or mmho/cm) (Figure 2), which corresponds to ionic strengths (I) of ∼0.005–0.04 M. Compared to marine (*I* ∼ 0.7 M), and other saline environments, the geothermal habitats sampled herein have significantly lower levels of dissolved ions, which would not be expected to impart significant environmental selection toward halophilic (high salt) or alkaliphilic (high-pH) microorganisms.

**Figure 3:**
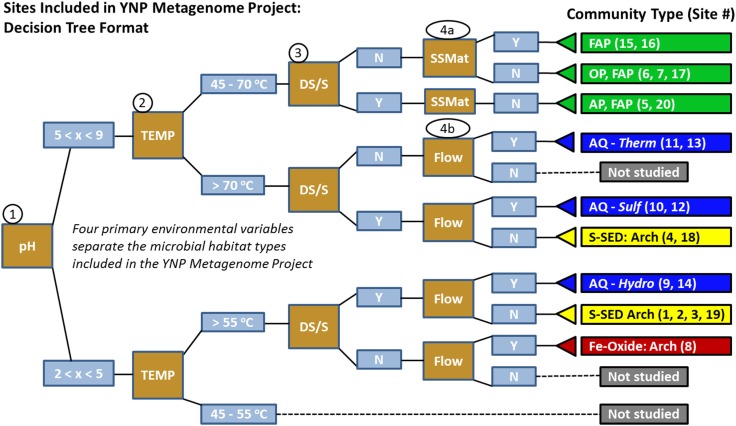
**The thermophilic microbial communities included in the YNP Metagenome Project were separated into predominant habitat types using a decision-tree with four “nodes” or primary environmental factors: pH, temperature (°C), the presence or absence of dissolved sulfide and/or elemental S, and additional physiographic context (4a: SSMat = sub-surface sample obtained from laminated phototrophic mats; 4b: Flow = primary flow channel)**. Decision nodes are represented by brown squares, node output by blue rectangles, and decision endpoints by triangles (i.e., predominant habitat types, site numbers). Targeted habitat types: FAP = filamentous anoxygenic phototrophs, OP = oxygenic phototrophs, AP = anoxygenic phototrophs, AQ-*Therm/Sulf/Hydro* = Aquificales “streamer” communities dominated by one of three major Aquificales lineages (i.e., *Thermocrinis*, *Sulfurihydrogenibium*, *Hydrogenobaculum*); S-SED = bottom or suspended sulfur-rich sediments dominated by archaea; Fe-Oxide = Fe-oxyhydroxide microbial mat dominated by archaea. The bulk aqueous characteristics of OPP_17 place this sample closest to sites WC_6 and CP_7 in the decision-tree, however, this sample was collected from biofilm growth on a large glass plate (see text, Table 1).

The current study was designed to cover a broad range of geochemical and temperature conditions, and to represent several of the major types of geothermal systems in YNP (Figure S1 in Supplementary Material). The breadth of habitats sampled capitalizes on a range of several key “system-defining” variables that give rise to different niches for thermophilic microorganisms. These key environmental variables and resulting habitat types are shown as a *decision-tree* (Figure 3), which is a descriptive tool to visualize the study design (showing replication of similar sites when possible). The nodes in the decision-tree also serve to represent hypotheses of the primary environmental factors that are most important for defining niches occupied by specific phylotypes. The corresponding metadata (geochemistry and physical context) associated with each of these sites are provided as supplemental material (Table S2 in Supplementary Material), but key attributes (T, pH, sulfide, or elemental S, and physical context) of each site are included here to understand the predominant site groupings, as well as the targeted communities and/or populations the study aimed to elucidate (Table 1; Figure 3).

**Table 1:** **Site names, abbreviations, and minimal set of environmental metadata necessary to separate different microbial community types (temperature, pH, dissolved sulfide or the presence of elemental S; Figure 3)**.

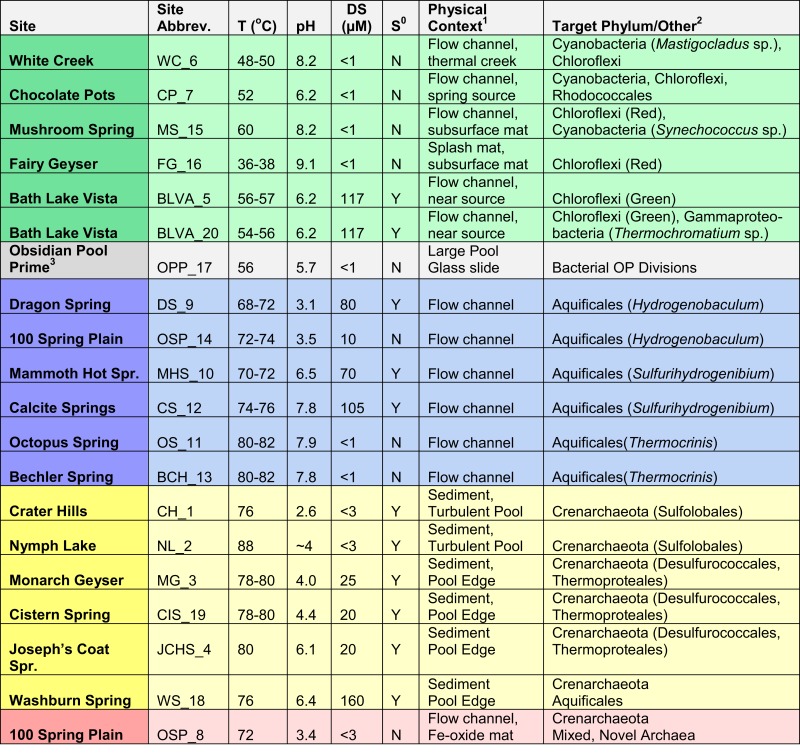

*The three major habitat types sampled include phototrophic mats (green), filamentous “streamer” communities (blue), and archaeal-dominated, elemental sulfur-rich or Fe-oxide sediments (yellow and red, respectively)*.

^1^Physical context refers to location of sample: Examples include: dissected sub-surface layer (under-mat) within a phototrophic mat; high-velocity channels containing filamentous “streamer” communities or down-gradient Fe-oxide mats; S-rich sediment in various geothermal pools

*^2^Target phylum as discussed in submitted “RI-Files” and based on prior molecular, culture, microscopic and/or other investigations performed by individual investigators included in this project (Table S1 in Supplementary Material)*.

*^3^The sample from Obsidian Pool Prime (OPP_17) represents a different habitat type and was obtained from a large thermal pool after colonization of a glass plate. Physicochemical characteristics place this sample closest to “phototrophic mats” in the current study*.

The separation of sites as a function of pH represents a key decision point in the study design. As the first node, pH allows for an important distinction between the effective temperature limit for colonization by known phototrophs in acidic (pH < 5) versus more neutral (pH 5–9) environments. It is well established that the upper temperature limit for oxygenic phototrophs is ∼70–74°C (Brock, 1978; Madigan et al., 2012), but cyanobacteria do not generally colonize habitats below pH 4.5–5. Consequently, at pH values < 5, a different phototrophic limit is established near 54–56°C, corresponding to the upper temperature limit of members of the Cyanidales (red algae), diatoms, or possible representatives from the mildly acidophilic purple-bacteria (Toplin et al., 2008; Madigan et al., 2012). Consequently, bacterial phototrophic mats are confined to the upper node of the decision-tree, and are not expected as dominant organisms in habitat types below pH 5. Diverse eukaryotic (algal) phototrophic mats exist in YNP (<55°C), but these were not considered in the current study. The phototrophic sites (5, 6, 7, 15, 16, and 20) were chosen to target communities dominated by either oxygenic phototrophs (OP), filamentous anoxygenic phototrophs (FAPs), or anoxygenic phototrophs (AP). As noted, the sample from Obsidian Pool Prime (OPP_17) was obtained from biofilm growth on a large glass plate and represents a different physical context relative to other phototrophic sites.

Both low- and high-pH sites are further separated based on the presence or absence of sulfide and/or elemental S (Figure 3). This is an important variable because high sulfide concentrations also imply low oxygen (hypoxia). When sulfide levels are above detection (or if significant elemental sulfur and/or thiosulfate are present), there is generally rapid abiotic consumption of oxygen by reduced S species (Nordstrom et al., 2005, 2009). This does not rule out the possibility that O_2_ influx from the atmosphere will influence the resulting microbial community, but systems with plentiful sulfide and/or elemental S will (i) contain little to no detectable dissolved oxygen, and (ii) contain species of S that can serve as potential electron donors or acceptors. Utilization of an O_2_ variable in the decision-tree does not offer the same utility; low O_2_ does not imply the presence of reduced S species. Some geothermal springs discharge non-sulfidic, hypoxic waters that contain other reduced constituents such as ferrous Fe (e.g., CP_7, OSP_8).

Two additional variables related to physical context are necessary to explain the study design and the corresponding habitat types included for metagenome analysis. Although these variables are included here as a simple *yes* or *no*, they represent important physical determinants of microbial community structure. Gradients in oxygen, light quantity, and quality, and sulfide are substantial as a function of mat depth and influence the distribution of microorganisms (Ward et al., 1998; Ramsing et al., 2000; van der Meer et al., 2005). For instance, sub-surface mat (SSMAT) layers are known to be less oxygenated and generally contain a greater abundance of FAPs relative to surface layers, which show a greater abundance of cyanobacteria (Ward et al., 2006; Klatt et al., 2011). Two of the phototrophic sites sampled in this study were from sub-surface mat positions at Mushroom Spring (MS_15) and Fairy Geyser (FG_16), where mat dissection was conducted to focus on novel FAP communities discovered in prior studies (Ward et al., 1998; Boomer et al., 2002), and because other metagenome investigations were focused on the top layers of the MS_15 phototrophic mats (Klatt et al., 2011; Liu et al., 2011). Other phototrophic samples (CP, WC, BLVA) were obtained from surface mats that occur within geothermal outflow channels, and thus flow is an implied physical variable that defines these habitats.

Hydrodynamic conditions (i.e., flow rate, turbulence) are crucial for defining the microenvironmental context of microorganisms in geothermal settings. All Aquificales “streamer” communities (DS_9, MHS_10, CS_12, OSP_14, OS_11, BCH_13) were obtained within the primary flow path of high-velocity (0.1–0.3 ms^−1^) outflow channels, as well as site OSP_8 (Fe-oxide mat), which is immediately down-gradient of OSP_14. Members of the deeply rooted bacterial Aquificales are known for assuming filamentous morphology in turbulent environments where the discharge of hot reduced waters occurs in the presence of air or oxygenated waters (e.g., marine hydrothermal vents, Reysenbach et al., 2000). Flow and turbulence of discharged geothermal waters encourages the degassing of H_2_S(aq) as well as the ingassing of O_2_(g) (Inskeep et al., 2005; Nordstrom et al., 2005). Consequently, the high-velocity, stream-channel habitats are considerably different from geothermal pools, which accumulate sediments of various sulfides, S^0^, alunite, kaolinite, and polymorphs of SiO_2_, depending on specific geochemical conditions such as pH, and levels of H_2_S, As, Sb, and Fe (Inskeep et al., 2009). Sulfidic and elemental S-rich sediments (CH_1, NL_2, MG_3, CIS_19, JCHS_4, WS_18) were sampled from low-flow habitats over a wide pH range (2.5–6.1) at temperatures considerably greater than the phototrophic limit (Figure 3). Sulfidic sites less than pH 5–6 can be further separated based on pH, an important variable influencing the distribution of specific crenarchaea (e.g., organisms within the order Sulfolobales increase in abundance with decreasing pH).

The oxidation of aqueous Fe(II) to solid-phase ferric oxyhydroxide is an exergonic reaction under most hydrothermal conditions (Amend and Shock, 2001; Amend et al., 2003; Inskeep et al., 2005), but of course requires dissolved O_2_. The in-channel flow environments promote equilibration with atmospheric conditions and encourage O_2_ influx. The acidic Fe-oxide mat (0.5–1 cm depth) sampled in Norris Geyser Basin (OSP_8) occurs within a high-velocity channel where oxygenation results in O_2_(aq) concentrations of ∼ 40–60 μM, which are 20–30% of theoretical saturation at this temperature and pressure. The concentration of Fe(II) does not appear in the decision-tree, because pH and the inference of oxygen (e.g., lack of sulfide) are already included as major determinants (concentrations of total Fe generally increase three orders of magnitude per pH unit decrease). Consequently, it is not the concentration of Fe *per se* that determines whether Fe(II) is oxidized, but whether the hydrodynamic or physical setting promotes oxygenation. For example, the more acidic CH_1 site contains ∼5 times more soluble Fe(II) than OSP_8 (250 versus 45 μM), but due to the consistent geothermal delivery of low levels of H_2_S(g) and the ubiquity of solid-phase elemental sulfur in CH_1, the consumption of any O_2_ is likely driven by reduced phases of sulfur rather than by Fe(II). Consequently, Fe-oxides are not present in the thermal pool of CH_1, although evidence of these phases was observed where the air-water interface meets rocks along the edge of the pool.

The study design captures a significant swath of high-temperature habitats in YNP. It is important to integrate the primary variables responsible for the separation of these habitat types together with analysis and interpretation of the metagenomes. The decision-tree (Figure 3) is also useful for identifying habitat types not included in the study, such as acidic geothermal habitats less than 55–60°C, in which members of the Cyanidales (red algae) are commonly observed (Toplin et al., 2008), and in which moderately thermophilic heterotrophic bacteria are known to increase in abundance (Macur et al., 2004; Kozubal et al., 2012). The sample collected at Obsidian Pool Prime (OPP_17) represents a different habitat context relative to other sites included in the study and was obtained from 2 months growth on a large (625 cm^2^) glass slide suspended in the water column at 56°C (pH = 5.7). OPP is a large pool (∼0.5 ha) receiving mixed water inputs, and is the only sample in the study to be collected from the photic zone of a large water-body, albeit heavily influenced by geothermal inputs. Although the physical context is considerably different than other springs and pools included in the study, characteristics of OPP_17 place it near the phototrophic sites in the decision-tree (Figure 3).

### Sequencing overview and major assemblies

**Figure 4:**
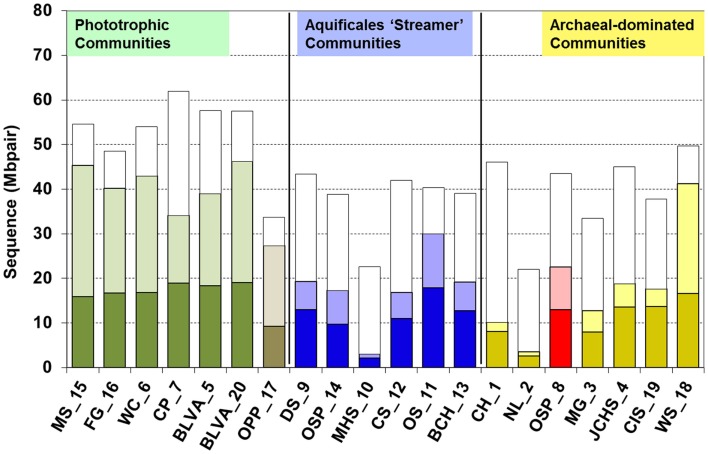
**Total amount of Sanger sequence (Mbpair) obtained for each site (top of open bars)**. The sum of assembled sequences (e.g., sum of all contig lengths) as well as that for all singleton sequences is shown as dark and light stacked bars, respectively [e.g., 62 Mbp of total sequence was obtained for CP_7, which resulted in 19 Mbp of assembled sequence (total contig length) and ∼14 Mbp of singleton sequences].

One of the primary aims of the study was to utilize the distinct geochemical differences across these 20 sites as a basis for understanding the distribution and function of different thermophiles. Moreover, given the uncertainties in predicting *in situ* metabolism solely using thermodynamic favorability or inference based on distantly related cultured isolates, it is useful to determine the actual organisms and genes present in high-temperature habitats as a means of constraining the metabolic possibilities and focusing our efforts on specific electron transfer reactions or catabolic pathways. The total metagenome sequence (Sanger) per site ranged from 20 to 60 Mb (Figure 4), but the ratio of *assembled* sequence to total sequence varied considerably across these microbial communities. The total assembled sequence (i.e., the sum of total contig length) reflects the collapse of redundant sequence reads into contigs that correspond to the predominant populations present (Table 1). Consequently, for communities dominated by a single phylotype (e.g., MHS_10, NL_2), the total assembled sequence was only ∼2 Mbp, and there were few singlet reads remaining after assembly (nearly 90% of the sequences assembled into contigs in these cases). In contrast, more assembled sequence was obtained in more diverse communities with a greater number of dominant phylotypes (e.g., CP_7, BLVA, OS_11, WS_18), but at lower contig coverage. Moreover, the significant number of singlet reads remaining after assembly in many sites suggested that the sequencing coverage was insufficient to allow genome assembly of potentially important members of these communities.

**Figure 5:**
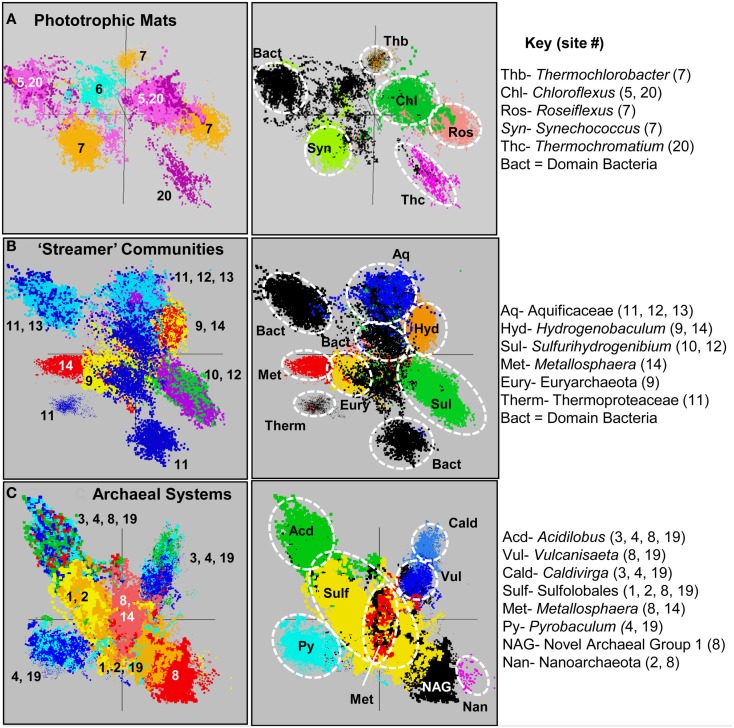
**Principal components analysis of oligonucleotide word frequencies (i.e., k-mers) of assembled metagenome sequence data for each group of sites**. **(A)** Phototrophic, **(B)** “Streamer,” and **(C)** Archaeal. Left Column: Assembled sequence data colored by site [*Phototrophic Mats*: BLVA_5, 20 = light-purple, dark-purple; WC_6 = light-blue; CP_7 = brown; MS_15 and FG_16 did not contain a significant number of contigs > 10,000 kb. “*Streamer*” *Communities*: DS_9 = yellow; OSP_14 = red; MHS_10 = green; CS_12 = violet; OS_11 = dark-blue; BCH_13 = light-blue. *Archaeal Systems*: CH_1 = gold; NL_2 = yellow; MG_3 = green; CIS_19 = light-blue; JCHS_4 = dark-blue; OSP_8 = red; OSP_14 = pink). Sites OPP_17 and WS_18 not included. Right Column: Phylogenetic assignment of sequence clusters (identical PCA orientation) based on comparative sequence analysis of all contigs to reference genomes (APIS, Badger et al., 2006).

The predominant phylotypes present in these three sample groups include expected microorganisms that have been the focus of prior studies at these exact locations (Table 1), as well as novel microorganisms in both the *Bacteria* and *Archaea*. Members of the Chloroflexi, Cyanobacteria, Chlorobi, Firmicutes, Bacteroidetes, Acidobacteria, and Proteobacteria dominated the phototrophic mats (Figure 5). The only sulfidic phototrophic site (BLVA) included in the study was sampled twice (∼8 months apart), before and during a bloom of purple sulfur bacteria (Gamma-proteobacteria), and both samples contained a strong signature of green Chloroflexi. Each of the in-channel “streamer” communities were dominated by one of three major Aquificales lineages specialized to variations in pH and sulfide (Figure 5). However, there was considerable variation in the other organisms present across the six Aquificales communities. The low-pH Aquificales communities (DS_9, OSP_14) contained significant sub-populations of different archaea (separated by the amount of sulfide), as did sites with higher pH values (OS_11, CS_12, BCH_13). In contrast, the MHS_10 sample contained no archaeal sequence and was dominated by one genus within the Aquificales (*Sulfurihydrogenibium*-like).

The high-temperature sulfur sediments and Fe-oxide mat samples were dominated by archaeal sequence reads (i.e., >85%) and contributed an extensive diversity of archaeal protein families (TIGRFAMS) not currently represented in environmental metagenome data, with the exception of replicate sites included in a smaller study of YNP chemotrophic communities (Inskeep et al., 2010). The sites dominated by Sulfolobales (CH_1, NL_2) represented a low-pH extreme and these communities are compared in more detail to other sulfur sediments at moderate, but slightly acidic pH values (pH 4–6), which are dominated by members of the orders Desulfurococcales and Thermoproteales (sites MG_3, JCHS_4, CIS_19) (Figure 5). In contrast, the acidic Fe-mats (OSP_8, 14) contained Fe(II)-oxidizing Sulfolobales (*Metallosphaera* sp.) and novel archaeal populations observed only in the absence of significant levels of dissolved sulfide (Table 1; Figure 5).

### Relative distribution of protein families

**Figure 6:**
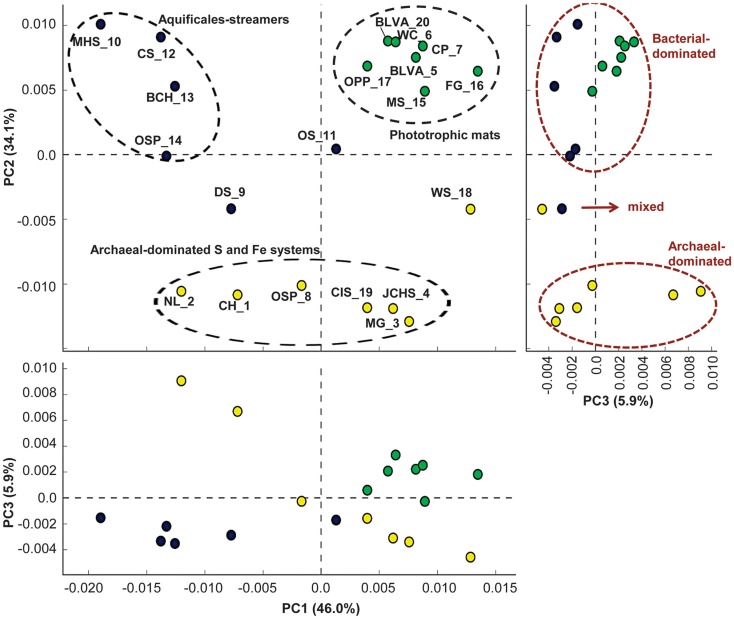
**Principal components analysis of normalized TIGRFAM protein family abundance data across all 20 sites**. The three panels show pairwise plots of the first three principal components (PC1 and PC2 account for 80% of the variation across sites, while PC3 only represents ∼6%). The first and second components separate sites into the three main habitat types studied (site labels and color groups are described in Table 1).

To provide an independent assessment of the three major site groups, we analyzed broad functional differences among the 20 sites using relative TIGRFAM protein family abundances present in the metagenomes. TIGRFAM protein families represent a relatively complete set of manually curated prokaryotic protein family models, which makes them suitable for the type of comparison we sought to perform. Abundance data, which were weighted by the scaffold assembly coverage, were obtained by counting protein copy numbers in assembled scaffolds for each site. Principal Component Analysis (PCA) of the TIGRFAM abundance data (Figure 6) showed that the first two principal components accounted for 80.1% of the functional variation among sites, and a two component plot (PC1 versus PC2) clearly separates the three major types of sites included in the study (phototrophic mats, “filamentous streamer communities,” and archaeal-dominated S or Fe sediments). In particular, Component 2 appeared to measure the relative fraction of bacterial versus archaeal sequence in the metagenome. For example, although sites DS_9 and OSP_14 contain a dominant Aquificales population, these sites were more similar to the archaeal group because of the presence of sub-dominant (<30%) archaeal populations (albeit different archaeal phylotypes in each site). Although site OS_11 also contained a major Aquificales population, the community contained significant sub-populations of novel bacterial groups that were also found in phototrophic mats (e.g., Bacteroidetes, Firmicutes), and thus fell closer to the phototrophic group. Site WS_18 contained at least three major archaeal populations and was included with the archaeal group, but this sediment sample also contained at least two major bacterial populations that plotted away from the main archaeal cluster in TIGRFAM PCA space (Figure 6).

Comparing groups, the Aquificales “streamer” communities and archaeal-rich habitats yielded broader functional diversity than phototrophic sites because the chemotrophic sites included both bacteria and archaea while the phototrophic sites were dominated by bacteria. The phototrophic mats contained very few sequences attributable to archaea or members of the Aquificales. Component 1 reflects the amount of Aquificales versus phototrophs (e.g., important for bacterial sites) as well as the amount of sequence data attributable to members of the Sulfolobales (especially relevant for archaeal sites), in which the content of Sulfolobales decreased from left to right (see PC1, Figure 6). Component 3 separates two extreme sites (CH_1 and NL_2) that were dominated by Sulfolobales (Figure 5) from the majority of other sites, although PC3 explains only 5.9% of the variation across sites.

**Figure 7:**
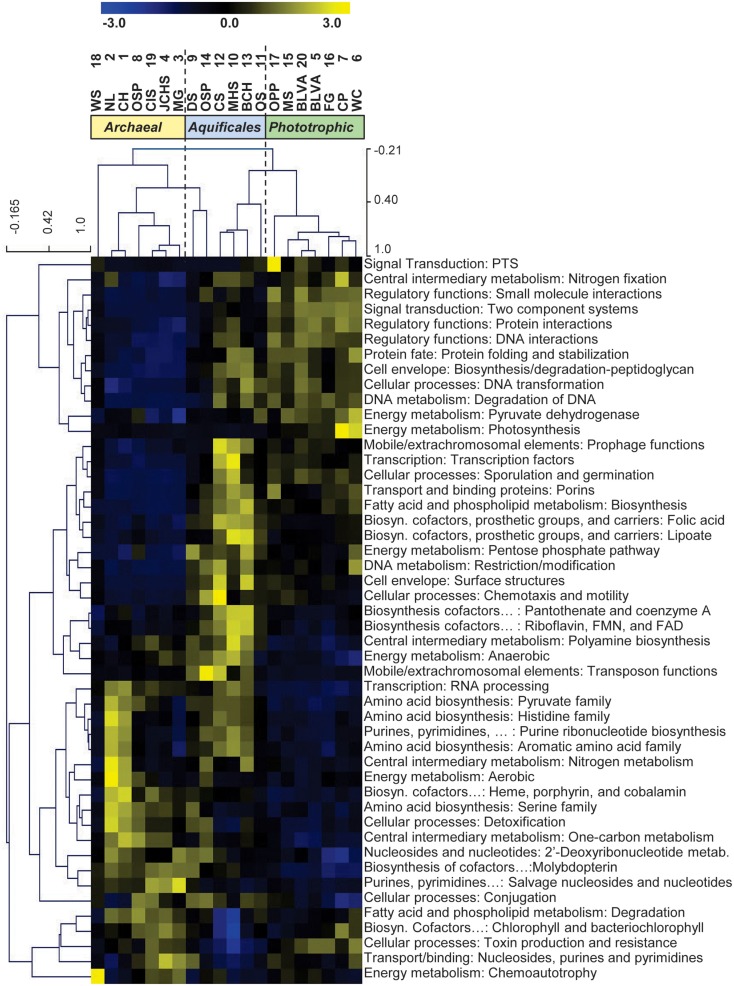
**Two-way hierarchical clustering of normalized TIGRFAM protein family abundance data averaged across intermediate-level TIGRFAM functional categories**. The data was standardized (subtract mean and divide by standard deviation) across sites before clustering so that the color scale units are in standard deviations from the mean across sites. Yellow colors correspond to values that are higher than the site mean and blue colors to values that are lower than the mean.

To identify what functional categories underlie the differences among sites, we obtained average TIGRFAM (Selengut et al., 2007) abundances for each functional category and site, and clustered these values using two-way hierarchical clustering (Figure 7). The site clustering tree based on TIGRFAM abundances showed remarkable similarity to the decision-tree (Figure 3), which indicated that the environmental variables separating the major habitat types (especially pH, temperature, and reduced sulfur versus oxygen) are key determinants of microbial community structure and function. The TIGRFAM categories that varied most significantly among sites included a broad array of proteins important in central metabolism, cell replication, motility, photosynthesis, electron transport, and other metabolic functions (Figure 7). The observed site separation within a TIGRFAM category could result from several factors that would not be evident without further investigation. These include the possibility that house-keeping functions simply reflect distinct phylogenetic differences observed across sites, and/or alternatively, that the range in abundance within a category may reflect the presence versus absence of specific metabolic capabilities contributed by phyla unique to a site. Consequently, the separation of sites using broad functional categories (Figure 7) accounts for both phylogenetic and functional differences, and provides strong support for the major site groupings emphasized in the three accompanying articles (Inskeep et al., 2013a,b; Takacs-Vesbach et al., 2013).

Specific TIGRFAM categories that varied in abundance across site groups included differences in functional categories such as nitrogen fixation and photosynthesis, expected to be highly represented in phototrophic sites (Figure 7). In contrast, TIGRFAMS that included cofactor biosynthesis (folic acid and lipoate), surface structures, fatty-acid biosynthesis, and chemotaxis/motility were more abundant in “streamer” communities. The archaeal sites exhibited a greater abundance of TIGRFAMs that included RNA processing, amino acid biosynthesis, nitrogen metabolism, aerobic respiration, and detoxification (Figure 7). In some cases, differences in TIGRFAM abundance across sites may have resulted from phylogenetic differences; for example, archaeal genes encoding a specific function may not be recognized as part of a TIGRFAM category that has been established primarily from bacterial genomes. TIGRFAM categories that result in the greatest separation among the 20 sites included signal transduction, nitrogen fixation and regulatory functions, which represent a greater proportion of total sequences in phototrophic sites versus either Aquificales or archaeal communities (Figure 7; Figure S2 in Supplementary Material shows two of these TIGRFAM categories in greater detail).

**Figure 8:**
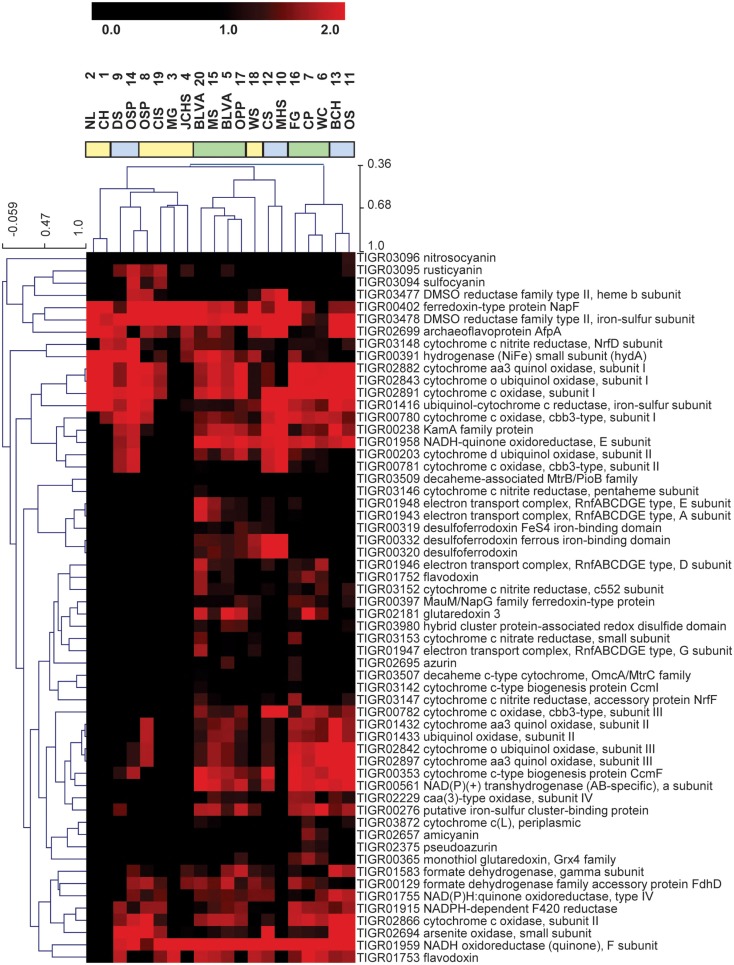
**Hierarchical clustering of metagenomes using relative TIGRFAM family abundances of genes in the TIGRFAM category “Electron Transport”**. Note that the abundance data has not been standardized, but rather log2-transformed (absolute abundances are shown).

More fine-grained functional differences among sites were obtained by only considering TIGRFAMs in a specific category such as “Electron Transport” (Figure 8). The site clustering based on the relative abundance of different electron transport domains provided a different view of the variation in functional attributes across sites. The distribution of specific respiratory complexes across sites [e.g., heme Cu oxidases (HCO), cytochrome *bd*-ubiquinol oxidases, blue Cu proteins, NiFe-hydrogenases, and nitrite/nitrate reductases] correlates more closely with geochemical parameters (e.g., oxygen, sulfur, hydrogen). Consequently, the distribution of different electron transport proteins across sites did not result in identical site clustering obtained using broader TIGRFAM categories (Figure 7). These observations are consistent with the fact that each group (i.e., phototrophic, archaeal, streamer communities) contained a range of sites with variable levels of sulfide or oxygen. Consequently, the factors contributing to functional variation within each of the three main site groups require additional dissection to appreciate how changes in these attributes are correlated with specific phylotypes (Inskeep et al., 2013a,b; Takacs-Vesbach et al., 2013).

The distribution and breadth of protein families observed in the current study represents a significant contribution to total protein diversity observed in all metagenomes currently found in the Integrated Microbial Genomes and Metagenomes (IMG/M) database (Markowitz et al., 2012) (as of spring 2012). This is due primarily to the fact that the current study focused on high-temperature communities rich in Aquificales, other deeply rooted novel bacteria, and numerous lineages of archaea. The functional diversity of the 20 Yellowstone geothermal samples included in this study was compared to other types of microbiomes as represented by the metagenomic sequence sets available in IMG/M, and based upon the TIGRFAM protein family abundance profiles for the assembled metagenomes. YNP samples are clearly separated from soil, water, gut, and other types of microbial communities in the public database (Figure S3 in Supplementary Material). As might be expected, the archaeal-dominated sites are most distant from the majority of public metagenomes, as few metagenome studies have targeted communities with significant archaeal populations. For example, the Crater Hills (CH_1) and Nymph Lake (NL_2) sites contained 1–2 predominant Sulfolobales populations and represented one extreme in the TIGRFAM PCA plots. Moreover, nearly identical samples obtained ∼2 years prior to this study (Inskeep et al., 2010) from four of the sites discussed here (CH_1, JCHS_4, CS_12, MHS_10) grouped with their expected replicate using PCA of TIGRFAM categories. Phototrophic mats and Aquificales “streamer” communities, which are dominated by eubacteria, fall closer to publicly available environmental metagenomes. The first principal component (which accounted for 24.8% of total variability in the TIGRFAM protein family abundance data across all sites in the database) almost entirely represents variation that exists within the YNP samples alone. The dramatic segregation of TIGRFAMS from YNP metagenomes versus previously sequenced mesophilic community metagenomes is indicative of the extensive contributions attributable to previously undescribed functional diversity.

## Summary

The metagenomes and corresponding metadata discussed here and in the accompanying articles provides a significant foundation of phylogenetic and metabolic information relevant to a wide range of geothermal ecosystems in YNP. Although the total sequence data obtained on an individual site basis was not sufficient to achieve assembly of all major phylotypes present in these distinct habitats, this study makes a considerable step toward understanding how microbial community structure and metabolic potential vary across a wide range of environmental parameters. A comparative distribution of protein families identified in the metagenome sequence reflects the major differences in phylogenetic structure among three primary groups of sites studied: phototrophic mats, Aquificales “streamer”-communities, and archaeal-dominated sediments. The systematic selection of geochemically distinct sites provides an enormous opportunity to link individual phylotypes (and associated metabolic attributes) with specific physicochemical properties of the habitats they occupy.

## Materials and Methods

### Geochemical analysis

Twenty geothermal sites in Yellowstone National Park (Table 1) were sampled and characterized in 2007–2008 (Figure S1 in Supplementary Material). Parallel samples of the bulk aqueous phase (<0.2 μm) and sediment intimately associated with the microbial community were obtained simultaneously and analyzed using a combination of field and laboratory methods. Temperature, pH, and redox-sensitive species (Fe^2+^/Fe^3+^; total dissolved sulfide; dissolved O_2_) were determined using field methods (with some exceptions given the number and diversity of sites studied) as described in more detail in previous reports (Langner et al., 2001; Macur et al., 2004). Total dissolved ions were determined using inductively coupled plasma (ICP) spectrometry and ion chromatography (for all major cations, anions and trace elements). Dissolved gases (CO_2_, H_2_, CH_4_) were determined using closed headspace gas chromatography (Inskeep et al., 2005) of sealed serum bottles (<0.2 μm) obtained in the field without air contact. A complete dataset of geochemical information corresponding to these samples is provided (Table S2 in Supplementary Material). Selected sediment and microbial mat samples were analyzed using scanning electron microscopy (Phillips Field Emission-SEM) in combination with energy-dispersive analysis of x-rays (EDAX) as well as x-ray diffraction (XRD). Thermodynamic calculations adjusted for temperature effects and performed using site-specific activities of dissolved and solid-phase constituents (Amend and Shock, 2001) showed that numerous oxidation-reduction reactions are exergonic (i.e., energy-yielding) in these types of geothermal systems and could support chemolitho- or chemoorgano-trophic metabolisms (Amend et al., 2003; Inskeep et al., 2005; Shock et al., 2005). The oxidation of energy-rich, reduced constituents such as H_2_, CH_4_, H_2_S, S^0^, and As(III) is extremely favorable when geothermal waters are exposed to atmospheric O_2_, or in some cases using alternate electron acceptors such as nitrate, ferric Fe, sulfate, or elemental S^0^. Dissolved Fe(II) and ammonium (NH_4_) concentrations can be very high in certain YNP geothermal systems, and offer yet another set of exergonic reactions that serve as a potential geochemical niche for chemotrophic organisms.

### DNA extraction and library construction

A standard DNA extraction protocol was used for the majority of samples, however, several sulfur sediments were ultimately subjected to various extraction kits to generate sufficient DNA yields for library construction. Our main emphasis was to obtain representative, unbiased environmental DNA for construction of small-insert libraries. Briefly, 3–25 g wet samples were extracted with 1 ml of Buffer A (200 mM Tris, pH 8; 50 mM EDTA; 200 mM NaCl; 2 mM sodium citrate; 10 mM CaCl_2_) with lysozyme (1 mg/ml final concentration) for 1.5 h at 37°C. Proteinase K (final concentration 1 mg/ml) and SDS [final concentration 0.3% (w/v)] were then added and incubated for 0.5 h at 37°C. This first lysate was removed and the samples were re-extracted using bead-beating protocols. The two lysates were combined and extracted with phenol-chloroform, and the resulting DNA was re-precipitated in ethanol, treated with RNAase and quantified by gel electrophoresis and staining. Our intent was to avoid biasing the samples against organisms that may be difficult to lyse with chemical methods. The extraction kits (e.g., MoBio) used on several of the sulfur-containing sediment samples also included a modest physical lysis step. Across all 20 sites, the extracted DNA ranged in size from ∼4 to 12 kbp, and for many samples these procedures resulted in higher MW DNA ranging from 9 to 20 kbp.

### Random shotgun sequencing

Short-insert (∼3 kbp) pUC18 libraries were constructed from all sites, and preliminary sequencing of 5–10 megabases (Sanger), was performed as a quality control to determine if the initial sequencing results [via MEGAN (Huson et al., 2007) and blastx analysis] were consistent with the sample origin. All samples passed this quality checkpoint and were further sequenced to produce a total of 40–60 Mbp per site (Figure 4), with the exception of two sites. Both MHS_10 and NL_2 contained only one major population type that was covered sufficiently with ∼20 Mbp of Sanger sequence and these sites were also included for a half plate of 454 titanium pyro-sequencing (∼225 Mbp per sample), along with two additional samples (MG_3, JCHS_4). When combined with the total amount of Sanger sequencing performed (871 Mbp), this collectively represented nearly 2 Gbp of random shotgun sequence. 16S rRNA genes were also amplified from the DNA of each site using universal primers specific for bacteria and archaea, and one 384-well plate was sequenced from each successful library. Results from 16S rRNA gene sequencing were generally consistent with the phylogenetic signatures observed in the metagenome data, with the exception of one site (NL_2), presumably due to PCR amplification bias. For NL_2, the majority of 16S rRNA clones were Thermoproteales-like, but the random shotgun sequence data showed a predominant Sulfolobales-like population.

### Sequence assembly

Metagenome assembly was conducted using two approaches (Celera and PGA), which resulted in reasonably similar overall assembly statistics. Due to the slightly better scaffold construction and prior history using the Celera assembler (Rusch et al., 2007; Inskeep et al., 2010), these assemblies were subjected to more detailed phylogenetic and functional analyses. However, the presence of genes or protein families described here are found in both assemblies, and the major results discussed here are not significantly different using either set of assembled data. Automated tools in IMG were used for gene identification and annotation for both sets of assembled data, and both sets of data are available on IMG/M. The following parameters were used for the Celera Assembler (Version 4.0): doOverlapTrimming = 0, doFragmentCorrection = 0, globalErrorRate = 12, utgErrorRate = 150, utgBubblePopping = 1, and useBogUnitig = 0. For PGA assemblies (Zhao et al., 2008), the following parameters were employed: OverlapLen = 30; Percent = 0.75; Clearance = 30; ClipIdn = 77; ClipQual = 10; CutoffScore = 400; EndOverhang = 800; InOverhang = 500; MinCovRep = 50; MinLinks = 2; MinSat = 3; NumIter = 50; PenalizeN = 1; QualOverLim = 400; QualScoreCutoff = 200; QualSumLim = 3500; SimDiFac = 30; Verbosity = 1.

As mentioned above, four sites (NL_2, MG_3, JCHS_4, MHS_10) received both Sanger sequence and one-half plate of 454 titanium pyro-sequencing (also available on IMG/M). Due to the success of Sanger sequencing in generating significant assemblies from these four sites, the additional pyro-sequence is not discussed in great detail in the subsequent manuscripts. In all four of these sites, contig coverage increased significantly when the pyro-sequence reads were included. However, the analyses discussed here are mostly derived from the Sanger sequence data, in order to maintain a consistent analytical approach for the data from all 20 sites.

### Phylogenetic analysis of environmental sequence data

Phylogenetic analysis of random shotgun sequence reads was performed using a number of different approaches, and these yielded convergent information regarding the predominant phylotypes present in these geothermal sites. Individual sequence reads were analyzed using G + C content (mol%) distribution coupled with blastx and MEGAN (Huson et al., 2007) analysis. Genome-level phylogenetic analysis was accomplished using fragment recruitment of environmental sequence data to reference microbial genomes (Rusch et al., 2007). At the time of writing, the database contained reference microbial genomes for ∼1500 bacteria and 100 archaea, however, only a handful of microbial genomes currently serve as appropriate references for the indigenous organisms within these communities. Assembled metagenome sequences were also analyzed using three dimensional PCA plots of nucleotide word frequencies (Teeling et al., 2004) with a simultaneous phylogenetic classification based on APIS or on a blast-based classification (Badger et al., 2006; Rusch et al., 2007). Metagenome sequence reported here can be viewed with these utilities at http://gos.jcvi.org/openAccess/scatterPlotViewer.html.

### High-level TIGRFAM analysis of environmental sequence data

Assembled scaffolds were annotated as described in Inskeep et al. (2010) and predicted proteins from the scaffolds were assigned to TIGRFAM protein families (Selengut et al., 2007) using HMMER 3 (Eddy, 2011) with *e*-value cutoff of 1*e*−6. TIGRFAM family counts for each scaffold were multiplied by the average coverage for the scaffold and the coverage weighted counts across all scaffolds for a particular site were summed to obtain estimated total family counts for a site. Different sites were normalized so that the total count over all TIGRFAM protein families was kept constant. TIGRFAM families were categorized using a two-level functional classification described on the TIGRFAM website^2^. PCA and statistical analysis of site group differences was performed using the STAMP v2.0 software (Parks and Beiko, 2010). The ANOVA test and Benjamini-Hochberg FDR correction implemented in STAMP was used to test for differences between multiple site groups. Two-way clustering was done on row-standardized (across sites) average TIGRFAM category abundance data using the Euclidean distance metric and complete-linkage hierarchical clustering. The MeV 4.8 (Saeed et al., 2003) software package was used for clustering and visualization. For comparison with other metagenomes in the IMG system, TIGRFAM profiles were generated for all the sites in this study and other metagenomic data sets downloaded using the IMG web interface.

### Detailed functional analysis of environmental metagenomic sequence data

The assembled metagenome sequence data was also screened for specific functional genes corresponding to known or putative pathways in material and energy transfer. We were specifically interested in assessing metabolic potential for chemolithoautotrophy (CO_2_ fixation and electron transfer) in high-temperature geothermal systems. Query DNA sequences known to code for proteins important in the oxidation of reduced chemical constituents or the reduction of a terminal acceptor (Table S3 in Supplementary Material) were used to search the environmental sequence data. Environmental sequence fragments exhibiting sequence similarity (*e*-values < 10^−10^) to query sequences were then reanalyzed using blastp, and assessed individually using phylogenetic analysis of deduced protein sequences against known relatives, as well as fragment length relative to query length. False positives were minimized using this screening process. This included (i) sequences matching the correct protein family of the query sequence, but not the exact query sequence (e.g., Mo-pterin oxidoreductases versus a specific protein within this family); (ii) sequences matching a query sequence due to regions of sequence similarity, but were clearly associated with a gene or gene cluster with different function; and (iii) sequences that returned mis-annotated blastp relatives. It is also possible that our inventory of metabolic potential missed sequences related to a specific query gene. For example, some genes found in the metagenome data were of insufficient length relative to a specific query sequence (<40%) to make a definitive assignment. Moreover, the lower depth of coverage (<1×) of sub-dominant phylotypes precluded a complete functional analysis of these organisms.

### Sequence availability

All annotated metagenome sequence assemblies (Celera/PGA) are available through the DOE-JGI IMG/M website^3^ under IMG taxon OID numbers as follows (site order is identical to that presented in Table 1): Phototroph Sites [YNPSite06 (2022920004/2013515000), Site07 (2022920013/2014031006), Site15 (2022920016/2015219002), Site16 (2022920018/2016842003), Site05 (2022920003/2013954000), Site20 (2022920020/2016842008), and Site17 (2022920021/2016842005).]; Aquificales Sites [YNPSite09 (2022920010/2014031004), Site14 (2022920007/2013954001), Site10 (2022920015/2015391001), Site12 (2022920011/2014031005), Site11 (2022920012/2014031007), and Site13 (2022920006/2013515002).]; Archaeal Sites [YNPSite01 (2022920009/2014031002), Site02 (2022920014/2015219001, 2016842002), Site03 (2022920002/2014031003, 2016842001), Site19 (2022920017/2015219000), Site04 (2022920008/2013843003), Site18 (2022920019/2016842004), and Site08 (2022920005/2013515001).].

## Conflict of Interest Statement

The authors declare that the research was conducted in the absence of any commercial or financial relationships that could be construed as a potential conflict of interest.

## Supplementary Material

The Supplementary Material for this article can be found online at http://www.frontiersin.org/Microbial_Physiology_and_Metabolism/10.3389/fmicb.2013.00067/abstract

Supplementary Table S1 **Contributions of NSF Research Coordination Network Steering Committee and Working Group members to the Yellowstone Metagenome Community Sequencing Project (DOE_JGI CSP 787081)**.

Supplementary Table S2 **Geochemical parameters measured in the bulk aqueous (<0.2 μm) phase in 20 different geothermal systems sampled for metagenome analysis**.

Supplementary Table S3 **List of gene sequences and corresponding accession numbers used to query the assembled environmental sequence data for assessing potential metabolic attributes associated with the predominant phylotypes found within these geothermal sites**.

Supplementary Figure S1 **Additional site photographs emphasizing landscape context of geothermal habitats and field sampling efforts (included as a separate file containing 53 annotated photographs)**.

Supplementary Figure S2 **Top ranked TIGRFAM categories based on their ability to differentiate the three types of sites discussed in this study: aquificales “streamer” communities (blue), archaeal-dominated sediments (yellow), and phototrophic mats (green)**. **(A)** Signal transduction; **(B)** Regulatory functions (ANOVA test with Benjamini-Hochberg FDR multiple testing correction).

Supplementary Figure S3 **Principal components analysis of normalized TIGRFAM protein family abundance data across all 20 sites as well as a subset of diverse metagenomic datasets from IMG**. The metagenomes are colored based on rough classification of the environments from which the sample originates: *Yellowstone sites in this study* = red; *Yellowstone sites from other studies* = orange; *sediments* = turquoise; *soils* = pink; *aquatic* = light-blue; *fungus-garden* = green; *animal-gut* = brown; *enrichment community* = light-orange; *other samples* = gray.

## Footnotes

^1^www.rcn.montana.edu

^2^http://www.jcvi.org/cgi-bin/tigrfams/index.cgi

^3^http://img.jgi.doe.gov/m
