# Supplementary material for: The YNP Metagenome Project: Environmental Parameters Responsible for Microbial Distribution in the Yellowstone Geothermal Ecosystem
Source: Front Microbiol. 2013 May 6;4:67. doi: 10.3389/fmicb.2013.00067 (PMC3644721; doi:10.3389/fmicb.2013.00067)
Supplement: Supplementary Table S1 — Contributions of NSF Research Coordination Network Steering Committee and Working Group members to the Yellowstone Metagenome Community Sequencing Project (DOE_JGI CSP 787081). [file 41697_Inskeep_DataSheet1.ZIP › 41697_Inskeep_Table_S1.pdf]

Supplemental Table 1. Contributions of NSF Research Coordination Network Steering Committee and Working Group members to the Yellowstone Metagenome Community Sequencing Project (DOE_JGI CSP 787081).

Project Steering Committee

Name	Address	Site Interest	Contributions
Bill Apel	Idaho National Laboratory		Financial support for pilot JCVI study (Inskeep et al., 2010)
Don Bryant	Pennsylvania St. University	5, 15, 20	Phototrophic site selection, analysis approaches and manuscript review.
Jonathan Eisen	UC Davis - JGI		Input discussion at March 2009 YNP RCN/DOE-JGI working group meeting
Bruce Fouke	University of Illinois	10	Aquificales site selection, sample collection, and manuscript review.
Doug Rusch	J.Craig Venter Institute		Bioinformatics: nucleotide word frequency principal components analysis
Bill Inskeep	MSU-TBI	3,4,8,14,19	Project PI, RCN Director: Sample collection, site geochemistry, bioinformatics and manuscript preparation, submission, coordination and review.
Markus Herrgard	Synthetic Genomics		Bioinformatics: TIGRFAM/PFAM and principal components analysis
David Mead	Lucigen Corporation		Input discussion at March 2009 YNP RCN/DOE-JGI working group meeting
A-L. Reysenbach	Portland State University	10,11,12	Aquificales site selection, discussion and manuscript review.
Frank Roberto	Idaho National Laboratory	1, 2	Sample collection, DNA extraction (CH_1, NL_2).
John Spear	Colorado School of Mines	17,18	Site selection, sample collection and DNA extraction (OP_17).
Susannah Tringe	DOE-Joint Genome Institute		Project Oversight at JGI, Bioinformatics, manuscript preparation and review
Mark Young	MSU-TBI	1, 2	Sample collection, DNA extraction (CH_1, NL_2).

Working Group Members and Field Collaborators

Sarah Boomer	Western Oregon Univ.	16	Phototroph site selection, sample collection, and manuscript review.
Igor Brown	NASA	7	Phototroph site selection and working group discussion.
Eric Boyd	MSU-TBI	3,19	Archaeal site selection, sample collection, and manuscript review.
Seth D'Imperio	MSU-TBI	9	Sample collection and DNA extraction (Dragon Spring).
Natsuko Hamamura	MSU/PSU	11,12	Aquificales site selection, sample collection, DNA extraction and manuscript review.
Christie Hendrix	US NPS/YNP	All	Research Permitting
Zack Jay	MSU-TBI	All	NSF RCN Coordinator: Sample collection, DNA extraction, site geochemistry, bioinformatics and manuscript preparation.
Chris Klatt	MSU-TBI	5, 15, 20	Phototroph sample collection, DNA extraction, data analysis and manuscirpt preparation.
Richard Macur	MSU-CBE		Site selection, sample collection, geochemistry, and DNA extraction.
Tim McDermott	MSU-TBI	9	Site selection, sample collection.
Scott Miller	Univ. of Montana	6	Site selection, sample collection, and manuscript review.
Niki Parenteau	NASA	7	Site selection, sample collection, and manuscript review.
Beverly Pierson	University of Puget Sound	7, 16	Sample collection (Chocolate Pots, Fairy Geyser)
Tina Takacs-Vesbach	University of New Mexico	13	Aquificales sample collection, DNA extraction, data analysis and manuscipt preparation.
Dave Ward	MSU-TBI	5, 15, 20	Phototroph site selection, sample collection, and manuscipt review.
