# Supplementary material for: The YNP Metagenome Project: Environmental Parameters Responsible for Microbial Distribution in the Yellowstone Geothermal Ecosystem
Source: Front Microbiol. 2013 May 6;4:67. doi: 10.3389/fmicb.2013.00067 (PMC3644721; doi:10.3389/fmicb.2013.00067)
Supplement: Supplementary Table S1 — Contributions of NSF Research Coordination Network Steering Committee and Working Group members to the Yellowstone Metagenome Community Sequencing Project (DOE_JGI CSP 787081). [file 41697_Inskeep_DataSheet1.ZIP › 41697_Inskeep_Table_S3.pdf]

Table S3. List of gene sequences and corresponding accession numbers used to query the assembled environmental sequence data for assessing potential metabolic attributes associated with the predominant phylotypes found within these geothermal sites.

Gene	Protein	Length (aa)	Query Seq. Reference	Accession No.	Classifications
aoxB	cytochrome c oxidase (aa3-type), subunit 1	815	Aeropyrum pernix K1	gi|118431201	NULL
aroA	arsenite oxidase large subunit	1006	Aeropyrum pernix K1	gi|118431923	DMSO Reductase
aroA	arsenite oxidase large subunit	825	Alcaligenes faecalis	gi|41688465	DMSO Reductase
aroA	arsenite-oxidase large subunit	826	Cenibacterium arsenoxidans	gi|22758844	DMSO Reductase
aroA	arsenite oxidase, large subunit	836	Chloroflexus aurantiacus J-10-fl	gi|163846783	DMSO Reductase
aroA	molydopterin dinucleotide-binding region	1026	Pyrobaculum calidifontis JCM 11548	gi|126459978	DMSO Reductase
aroA	hypothetical protein ST2391	1133	Sulfolobus tokodaii str. 7	gi|15922722	DMSO Reductase
aroA	arsenite oxidase, large subunit	961	Thermus thermophilus HB8	gi|55773483	DMSO Reductase
arrA	dissimilatory arsenate reducatse	846	Chrysiogenes arsenatis	gi|51871102	DMSO Reductase
arrA	dissimilatory arsenate reducatse	652	Desulfosporosinus sp. Y5	gi|77743874	DMSO Reductase
arrA	dissimilatory arsenate reducatse	854	Shewanella sp. ANA-3	gi|117920784	DMSO Reductase
arrA	dissimilatory arsenate reducatse	740	Sulfurospirillum barnesii	gi|146230645	DMSO Reductase
arsB	arsenite efflux membrane protein, ars operon	411	Sulfolobus solfataricus P2	gi|15897099	NULL
arsB	arsenite efflux membrane protein, ars operon	397	Archaeoglobus fulgidus DSM 4304	gi|11499889	NULL
arsB	arsenite efflux membrane protein, ars operon	432	Sulfurihydrogenibium sp. YO3AOP1	gi|188996862	NULL
arsB	arsenite efflux membrane protein, ars operon	419	Hydrogenobaculum sp. Y04AAS1	gi|195952836	NULL
arsB	arsenite efflux membrane protein, ars operon	425	Pyrococcus abyssi GE5	gi|5459107	NULL
arsB	arsenite efflux membrane protein, ars operon	431	Thermoplasma volcanium GSS1	gi|14324502	NULL
arsB	arsenite efflux membrane protein, ars operon	426	Thermococcus kodakarensis KOD1	gi|57640815	NULL
arsC	arsenate reductase, ars operon	140	Acidiphilium cryptum JF-5	gi|148261794	NULL
arsC	arsenate reductase, ars operon	125	Acidovorax avenae subsp. citrulli AAC00-1	gi|120609609	NULL
arsC	arsenate reductase, ars operon	125	Alkalilimnicola ehrlichei MLHE-1	gi|114319987	NULL
arsC	arsenate reductase, ars operon	144	Aquifex aeolicus VF5	gi|15606093	NULL
arsC	arsenate reductase, ars operon	112	Desulfitobacterium hafniense Y51	gi|89894529	NULL
arsC	arsenate reductase, ars operon	112	Desulfotomaculum reducens MI-1	gi|134300073	NULL
arsC	arsenate reductase, ars operon	147	Geobacillus thermodenitrificans NG80-2	gi|138896594	NULL
arsC	arsenate reductase, ars operon	141	Methylobacterium extorquens PA1	gi|163851337	NULL
arsC	arsenate reductase, ars operon	120	Shewanella loihica PV-4	gi|127512768	NULL
arsC	arsenate reductase, ars operon	131	Staphylococcus aureus subsp. aureus USA3	gi|87161126	NULL
arsM	arsenite S-adenosylmethyltransferase	248	Methanosarcina mazei Go1	gi|21228345|	NULL
arsM	arsenite S-adenosylmethyltransferase	271	Rhodopseudomonas palustris BisB5	gi|91977888	NULL
arsM	arsenite S-adenosylmethyltransferase	266	Geobacter metallireducens GS-15	gi|78223987	NULL
arsM	arsenite S-adenosylmethyltransferase	273	Pelotomaculum thermopropionicum SI	gi|147676775	NULL
bisC	biotin sulfoxide reductase	777	E. coli	gi|6920066	DMSO Reductase
c579	cytochrome c579 - Fe oxidation	185	Leptospirillum sp. Group II UBA	gi|124514988	Fe oxidation
cbdA	cytochrome bd ubiquinol oxidase	457	Thermoplasma acidophilum DSM 1728	gi|16082030	terminal oxidase
cbsA	cytochrome b558/566, subunit A	474	Metallosphaera sedula	gi|42516539	Fe oxidation, cytochrome b558/566
cdc6-1	Cdc6-1	407	Hyperthermus butylicus DSM 5456	gi|124027479	cell division control
cdc6-2	cell division control protein 6	411	Hyperthermus butylicus DSM 5456	gi|124027782	cell division control

clrA	chlorate reductase	914	Ideonella dechloratans	gi|34494349	DMSO Reductase
cox1	Cytochrome-c oxidase, heme copper oxidase	472	Hydrogenobaculum sp. Y04AAS1	gi|195953198	heme copper oxidase
cueo	multicopper oxidase - type I, ii, and iii domains.	529	e-coli	gi|188496066	multi-copper oxidase
cyd	cytochrome bd-type quinol oxidase, subunit 1	455	Thermoplasma volcamium	gi|13540914	cytochrome d oxidase
dcytb	Cytochrome b561 / ferric reductase transmemb	190	Candidatus Methanoregula boonei 6A8 - an	gi|153999613	cytochrome B561, Fe reduction
dmsA	anaerobic dimethyl sulfoxide reductase	814	E. coli	gi|9911072	DMSO Reductase
dmsA	dimethylsulfide dehydrogenase	910	Rhodovulum sulfidophilum	gi|24110653	DMSO Reductase
doxB	cytochrome c oxidase, subunit 1	585	Metallosphaera sedula DSM 5348	gi|145703030	heme copper oxidase
dsrA	sulfite reductase, dissimilatory-type alpha subu	408	Chlorobium phaeobacteroides BS1	gi|189500785	Nitrite/Sulfite reductase
dsrA	sulfite reductase, alpha subunit	418	Archaeoglobus fulgidus DSM 4304	gi|11498035	Sulfite Reductase
fdh	Formate dehydrogenase Alpha subunit (fdhF-2	979	Sulfolobus solfataricus P2	gi|15899539	DMSO Reductase
fdh	Formate dehydrogenase	647	Thermofilum pendens Hrk 5	gi|119525654	DMSO Reductase
fdh	Formate dehydrogenase	673	Methanocaldococcus jannaschii DSM 2661	gi|15669895	DMSO Reductase
fer	Predicted ferric reductase	431	Bifidobacterium animalis subsp. lactis HN01	gi|183602244	Fe reduction
fer	ferric reductase, flavin reductase	446	Magnetospirillum gryphiswaldense MSR-1	gi|144898159	Flavin reductase
FhuF	ferric iron reductase - FhuF is involved in the re	345	Shewanella sp. MR-7	gi|114047021	Fe reduction, ferric iron reductase
flaB	flagellin	387	Thermotoga maritima MSB8	gi|4981286	Motility
flaB	flagellin-like protein	246	Aeropyrum pernix K1	gi|14601713	Motility
foxA	cytochrome c oxidase, subunit 1	570	Metallosphaera sedula DSM 5348	gi|145701519	heme copper oxidase
foxB	cytochrome c oxidase, subunit II	203	Metallosphaera sedula	gi|145701515	Fe oxidation, cytochrome C oxidase
foxC	hypothetical cytochrom b	548	Metallosphaera sedula	gi|145701513	Fe oxidation
FoxG	hypothetical protein Msed_0469	619	Metallosphaera sedula	gi|146303254	polyferridoxin, NapH
Fre3P	Ferric reductase, reduces siderophore-bound ir	711	Saccharomyces cerevisiae	gi|6324957	Fe reduction
Frp1	ferric-chelate reductase Frp1	564	Schizosaccharomyces pombe - fungi	gi|173388	Fe reduction, ferric iron reductase
hdrA	Heterodisulphide reductase subunit A - FAD-de	365	Metallosphaera sedula	gi|145702558	Pyridine nucleotide-disulphide
hdra	FAD-dependent pyridine nucleotide-disulphide	365	Metallosphaera sedula DSM 5348	gi|145702558	Pyridine nucleotide-disulphide
hdrb	hypothetical protein Msed_1546	444	Metallosphaera sedula DSM 5348	gi|146304309	heterodisulfide reductase
hdrB	Heterodisulphide reductase subunit B	306	Metallosphaera sedula	gi|145702555	Heterodisulfide reductase
hdrc	Heterodisulfide reductase subunit C-like protei	280	Metallosphaera sedula DSM 5348	gi|145702560	heterodisulfide reductase
hynL	nickel-dependent hydrogenase, large subunit	638	Pyrobaculum arsenaticum DSM 13514	gi|145591511	NULL
hynL	iron only hydrogenase large subunit	605	Syntrophus aciditrophicus SB	gi|85722031	NULL
hynL	periplasmic [NiFeSe] hydrogenase large subun	514	Desulfomicrobium baculatum	gi|172045678	NULL
hynL	H(2)-forming N(5),N(10)-methylenetetrahydrom	344	Methanothermobacter marburgensis	gi|2828203	NULL
hynS	membrane-bound NiFe hydrogenase	420	Acidianus ambivalens	gi|22204173	NULL
hynS	Nickel-iron dehydrogenase small subunit	432	Metallosphaera sedula DSM 5348	gi|145701974	NULL
hynS	hydrogenase (NiFe) small subunit HydA	423	Thermofilum pendens Hrk 5	gi|119524624	NULL
hynS	NiFe hydrogenase	506	Hyperthermus butylicus DSM 5456	gi|123978915	NULL
hynS	hydrogenase, Fe-only	579	Caldicellulosiruptor saccharolyticus DSM 89	gi|145410445	NULL
hynS	hydrogenase small subunit	353	Aquifex aeolicus VF5	gi|2983268	NULL
mco	multicopper oxidase - 2 type I blue copper dom	545	Metallosphaera sedula	gi|146303975	Blue copper protein, plastocyanin
mcrA	methyl-coenzyme M reductase I, alpha subunit	553	Methanococcus maripaludis S2	gi|45359122	methanogenesis/methanotrophy
merA	mercuric reductase	448	Metallosphaera sedula DSM 5348	gi|146304009	pyridine dinucleotide
merA	mercuric reductase	546	Geobacillus kaustophilus HTA426	gi|56421631	pyridine dinucleotide

merB	alkylmercury lyase	216	Staphylococcus aureus	gi|57157629	NULL
molyox	molybdopterin oxidoreductase	812	Thermofilum pendens Hrk 5	gi|119720027	DMSO Reductase
moxA	molybdopterin oxidoreductase	822	Archaeoglobus fulgidus DSM 4304	gi|11499961	DMSO Reductase
mtrA	decaheme cytochrome c MtrA - iron reduction	333	Shewanella oneidensis MR-1	gi|24373343	Fe reduction, decaheme c-type
mtrB	outer membrane protein precursor MtrB	697	Shewanella oneidensis MR-2	gi|24347596	Fe reduction, MtrB/PioB family
mtrC	decaheme cytochrome c MtrC -iron reduction?	735	Shewanella oneidensis MR-1	gi|24373345	Fe reduction, decaheme c-type
mtrC	decaheme cytochrome c	671	Shewanella oneidensis MR-1	gi|24373344	Fe reduction, decaheme c-type
napA	periplasmic nitrate reductase precursor	828	E. coli	gi|2506868	DMSO Reductase
narG	nitrate reductase, alpha subunit	1294	Pyrobaculum aerophilum str. IM2	gi|18314193	NULL
ndh	FAD-dependent pyridine nucleotide-disulphide	452	Thermofilum pendens Hrk 5	gi|119719061	Pyridine nucleotide-disulphide
nifD	nitrogenase_MoFe	532	Methanosarcina mazei	gi|27461018	Nitrogenase component 1 type
nifH	nitrogenase reductase	261	Rhizobium gallicum	gi|12004629	nitrogenase
nifH	RecName: Full=Nitrogenase iron protein; AltNa	280	Methanospirillum hungatei JF-1	gi|121697779	nitrogenase
nirK	nitrite reductase	352	Geobacillus thermodenitrificans NG80-2	gi|138894322	multi-copper oxidase
nirK	nitrite reductase	359	Haloferax denitrificans	gi|30089160	multi-copper oxidase
nirK	nitrite reductase	362	Natronomonas pharaonis DSM 2160	gi|76557314	multi-copper oxidase
nirK	nitrite reductase	180	Nitrosospira tenuis	gi|116734399	multi-copper oxidase
nirK	nitrite reductase	364	Bradyrhizobium sp. ORS278	gi|146191128	multi-copper oxidase
nirK	nitrite reductase	166	Nitrosomonas europaea	gi|116734407	multi-copper oxidase
nirS	cytochrome cd1 nitrite reductase	551	Hydrogenobacter thermophilus	gi|76667604	Nitrite Reductase
nirS	nitrite reductase (cytochrome C), conjectural	205	Pyrobaculum aerophilum str. IM2	gi|18314182	Nitrite Reductase
norB	nitric oxide reductase, cytochrome b subunit	721	Pyrobaculum aerophilum str. IM2	gi|18314186	heme copper oxidase
nosZ	nitrous oxide reductase	659	Haloarcula marismortui ATCC 43049	gi|55377286	NULL
nrfD	Polysulphide reductase NrfD molybdopterin oxi	402	Geobacter lovleyi SZ	gi|189426395	polysulphide reductase
nrfD	polysulphide reductase	349	Sulfolobus acidocaldarius DSM 639	gi|70607826	polysulphide reductase
perox	peroxiredoxin family protein	232	Hyperthermus butylicus DSM 5456	gi124027125	O2 detox
perox	Chain D, Crystal Structure Of Peroxiredoxin-Lik	171	Aquifex aeolicus	gi|160286241	O2 detox
pioA	Fe-oxidation in phototrophs, cytochrome	540	Rhodopseudomonas palustris TIE-1	gi|119331447	decaheme c-type cytochrome
pioB	Fe-oxidation in phototrophs, hypothetical	811	Rhodopseudomonas palustris TIE-1	gi|119331449	MtrB/PioB family
rdh	rhodanese-like domain protein	109	Acidithiobacillus ferrooxidans ATCC 23270	gi|218517303	rhodanese
rhodanes	Rhodanese domain protein	307	Sulfurihydrogenibium sp. YO3AOP1	gi|188996870	rhodanese
rhodanes	Rhodanese domain protein	126	Hydrogenobaculum sp. Y04AAS1	gi|195932447	rhodanese
rhodanes	rhodanese domain-containing protein	251	Caldivirga maquilingensis IC-167	gi|159041166	rhodanese
rhodanes	Rhodanese domain protein	133	Metallosphaera sedula DSM 5348	gi|145701813	rhodanese
rhodanes	thiosulfate sulfurtransferase	289	Aeropyrum pernix K1	gi|118431935	rhodanese
rhodanes	Rhodanese domain protein	281	Nitrosopumilus maritimus SCM1	gi|160339250	rhodanese
serA	selenate reductase	918	Thauera selenatis	gi|38605512	DMSO Reductase
sodA	superoxide dismutase	204	Thermus aquaticus	gi|217184	O2 detox
sodA	Superoxide dismutase	211	Sulfolobus solfataricus 98/2	gi|261601925	O2 detox
sor	sulfur oxygenase reductase	311	Sulfolobus tokodaii str. 7	gi|15921384	sulphur oxygenase reductase
sox	sulfite oxidase, molybdopterin	200	Metallosphaera sedula DSM 5348	gi|146303147	Sulfite Oxidase
soxB	cytochrome-c oxidase, subunit 1	550	Metallosphaera sedula DSM 5348	gi|146303075	heme copper oxidase
soxC	sulfide/sulfite oxidase, molybdopterin	427	Thermus aquaticus Y51MC23	gi|218294901	Sulfite Oxidase

soxC	sulfite dehydrogenase soxC precursor	430	Thermus thermophilus HB27	gi|46199348	Sulfite oxidase
soxD	sulfite dehydrogenase cytochrome subunit Sox	185	Thermus thermophilus HB28	gi|55981379	cytochrome c
soxM	cytochrome-c oxidase, subunit 1	801	Metallosphaera sedula DSM 5348	gi|146303109	heme copper oxidase
soxX	sulfur oxidation protein SoxX	183	Sulfurihydrogenibium sp. YO3AOP1	gi|188996461	cytochrome C
sqr	sulfide-quinone oxidoreductase	414	Sulfolobus solfataricus P2	gi|15899031	Pyridine nucleotide-disulphide
sreA	sulfur reductase molybdopterin subunit	1018	Acidianus ambivalens	gi|22213647	DMSO Reductase
sreA	molybdopterin oxidoreductase, molybdopterin b	1050	Sulfolobus solfataricus P2	gi|15898396	DMSO Reductase
sreA	molybdopterin oxidoreductase	882	Hydrogenobaculum sp. Y04AAS1	gi|156719515	DMSO Reductase
sreA	DMSO reductase chain A	984	Aquifex aeolicus VF5	gi|2983678	DMSO Reductase
Thioredo	Msed_1553	187	Metallosphaera sedula DSM 5348	gi|145702566	Thioredoxin
thioredox	thioredoxin reductase - annotated wrong, dihyd	440	Metallosphaera sedula DSM 5348	gi|145702564	Dihydrolipoamide dehydrogenase
torA	trimethylamine-N-oxide reductase	848	E. coli	gi|2506867	DMSO Reductase
tqoA	small subunit DoxA domain	166	Metallosphaera sedula DSM 5348	gi|146303149	DoxA
tqoB	small subunit DoxD	182	Metallosphaera sedula DSM 5348	gi|146303148	DoxD
ttrB	tetrathionate reductase	1020	Salmonella enterica Typhi str. CT18	gi|16760527	DMSO Reductase
ZntA	copper-transporting ATPase	695	Sulfolobus solfataricus P2	gi|15899608	ATPase, Hydrolase
