# Supplementary material for: The YNP Metagenome Project: Environmental Parameters Responsible for Microbial Distribution in the Yellowstone Geothermal Ecosystem
Source: Front Microbiol. 2013 May 6;4:67. doi: 10.3389/fmicb.2013.00067 (PMC3644721; doi:10.3389/fmicb.2013.00067)

Montana State University Thermal Biology Institute
NASA
Funding provided by: National Science Foundation
DOE Joint Genome Institute, US Department of Energy Office of Science
J. Craig Venter Institute

Supplemental Figure 1. Appendix Site Photographs for Yellowstone National Park (YNP)_Metagenome Project: DOE-JGI CSP 787081

1. Photographs are grouped by site types, then ordered by site number and site name (see Table 1, Inskeep et al., 2013) (Site types = Phototrophic mats, Aquificales ‘streamer’ communities, and archaeal-dominated sediments.

2. Comments in notes refer to details of slide location, mat types and scale reference .

3. All photographs require appropriate citation.

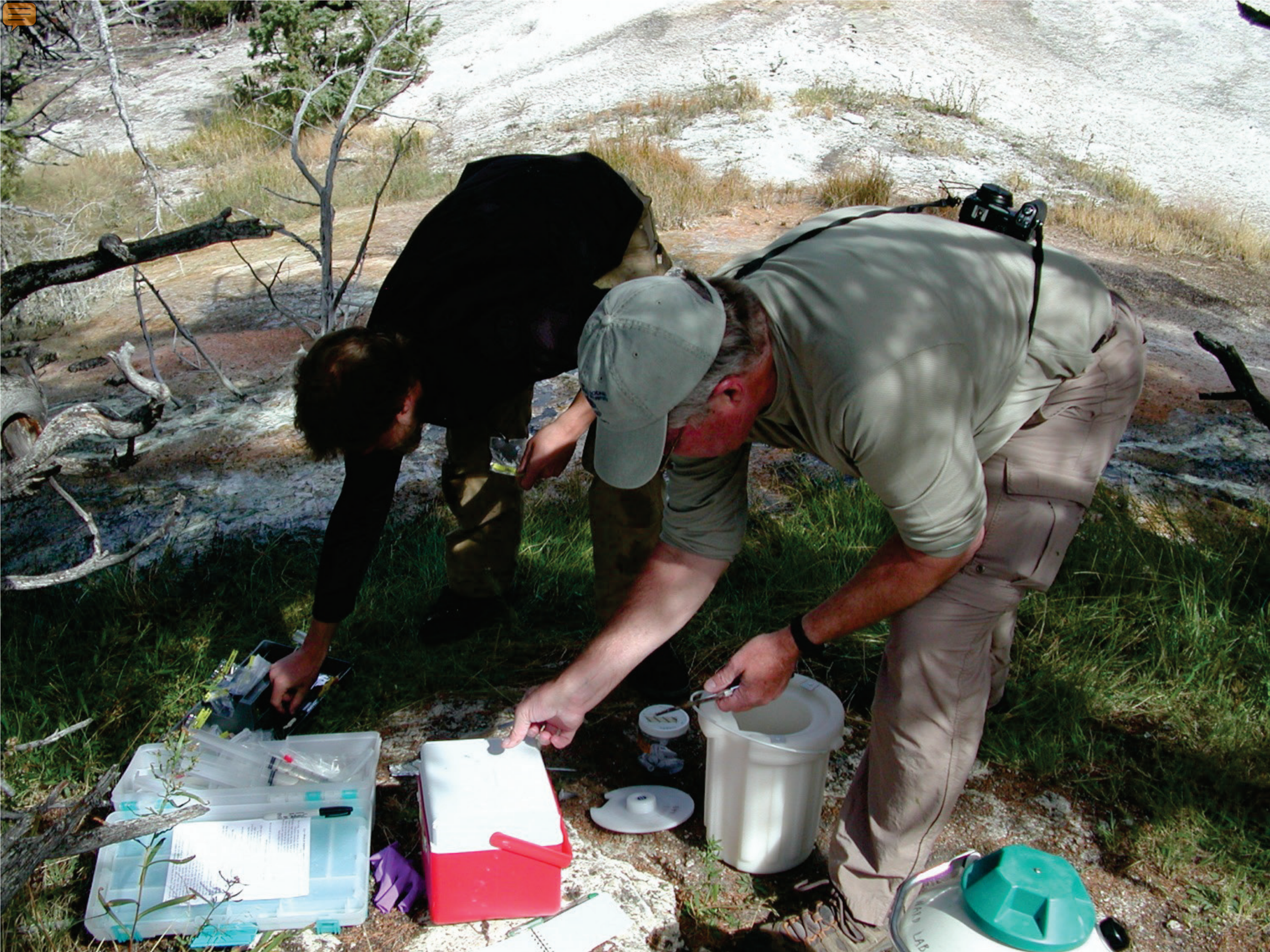

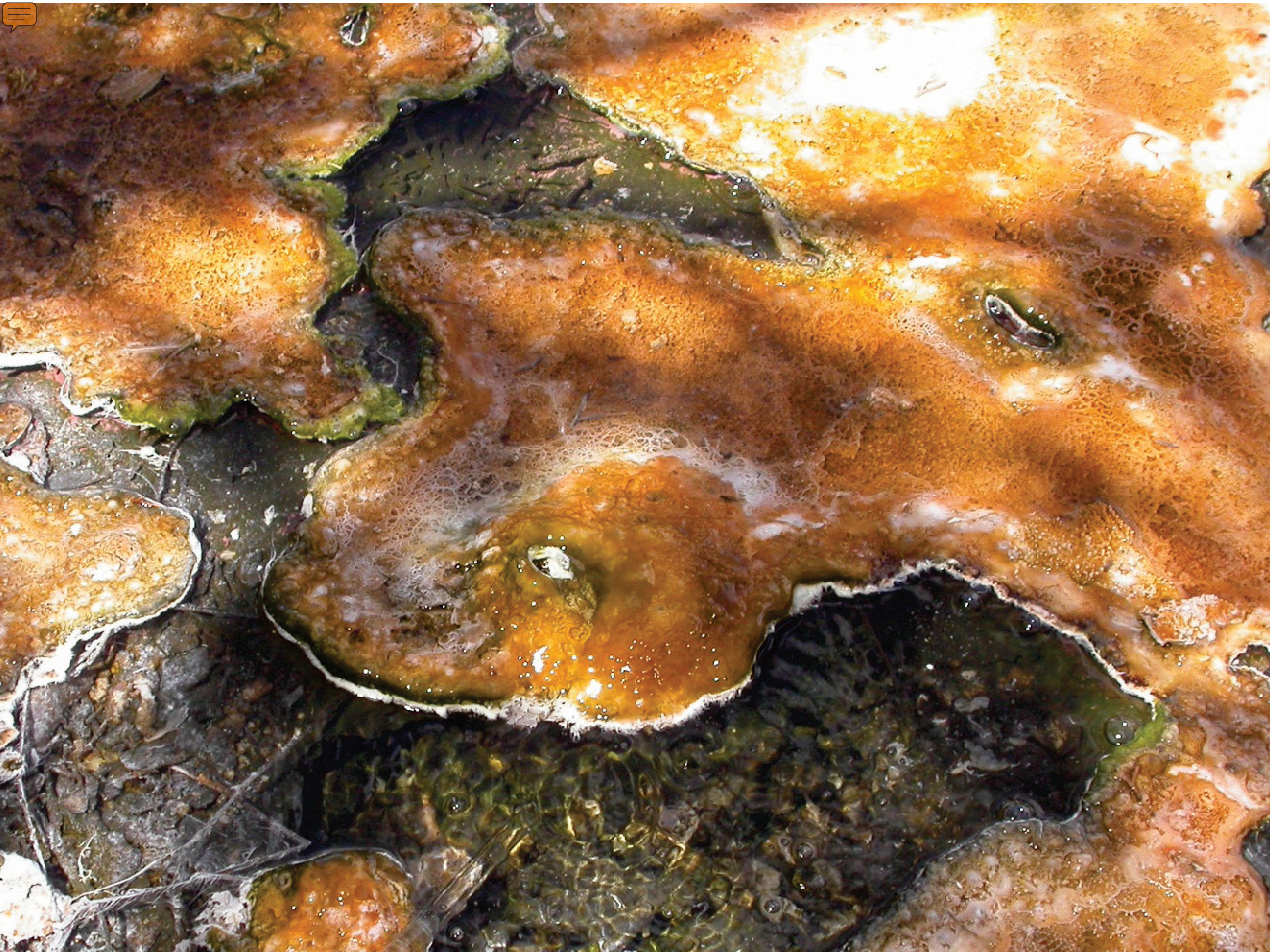

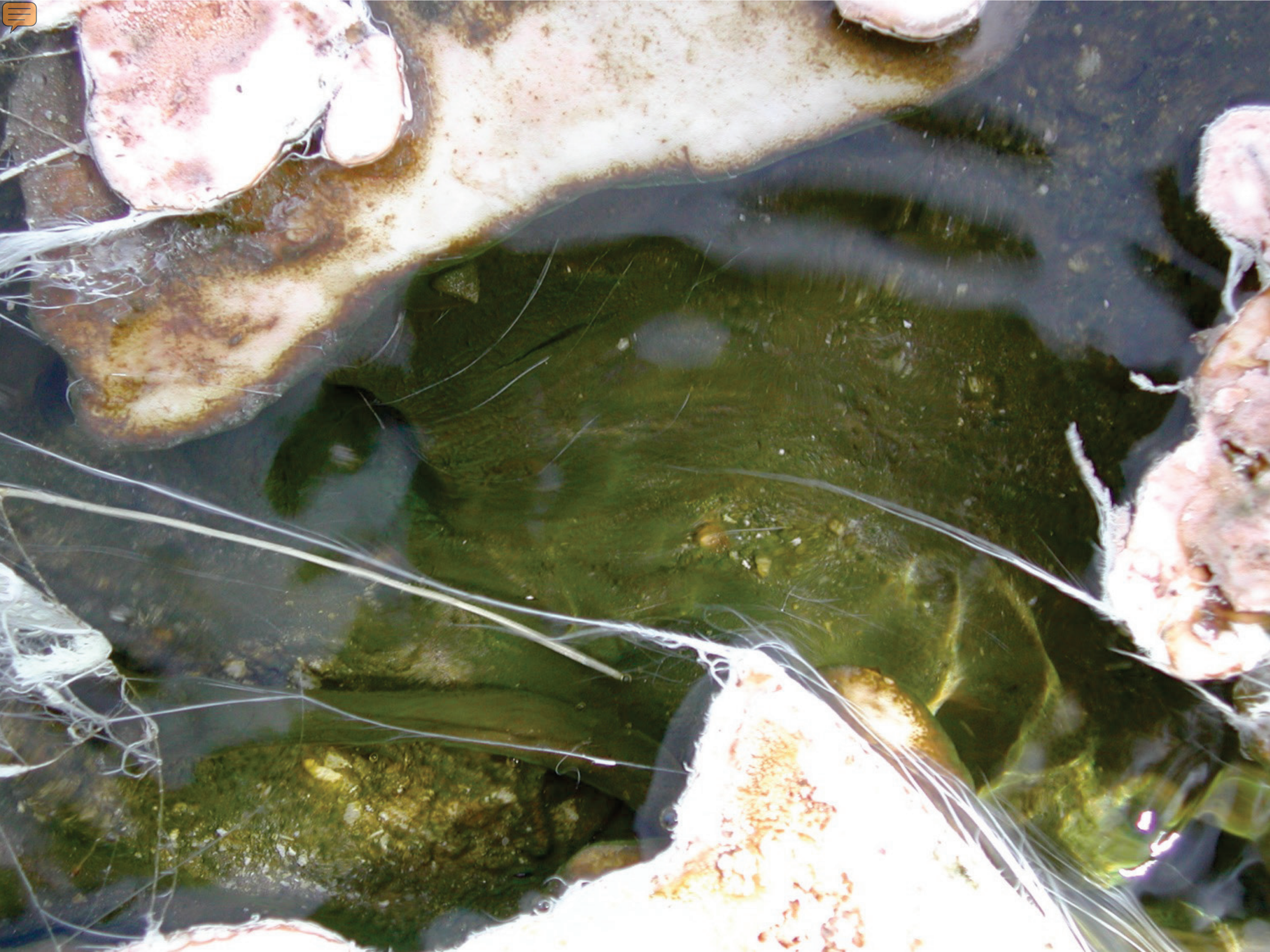

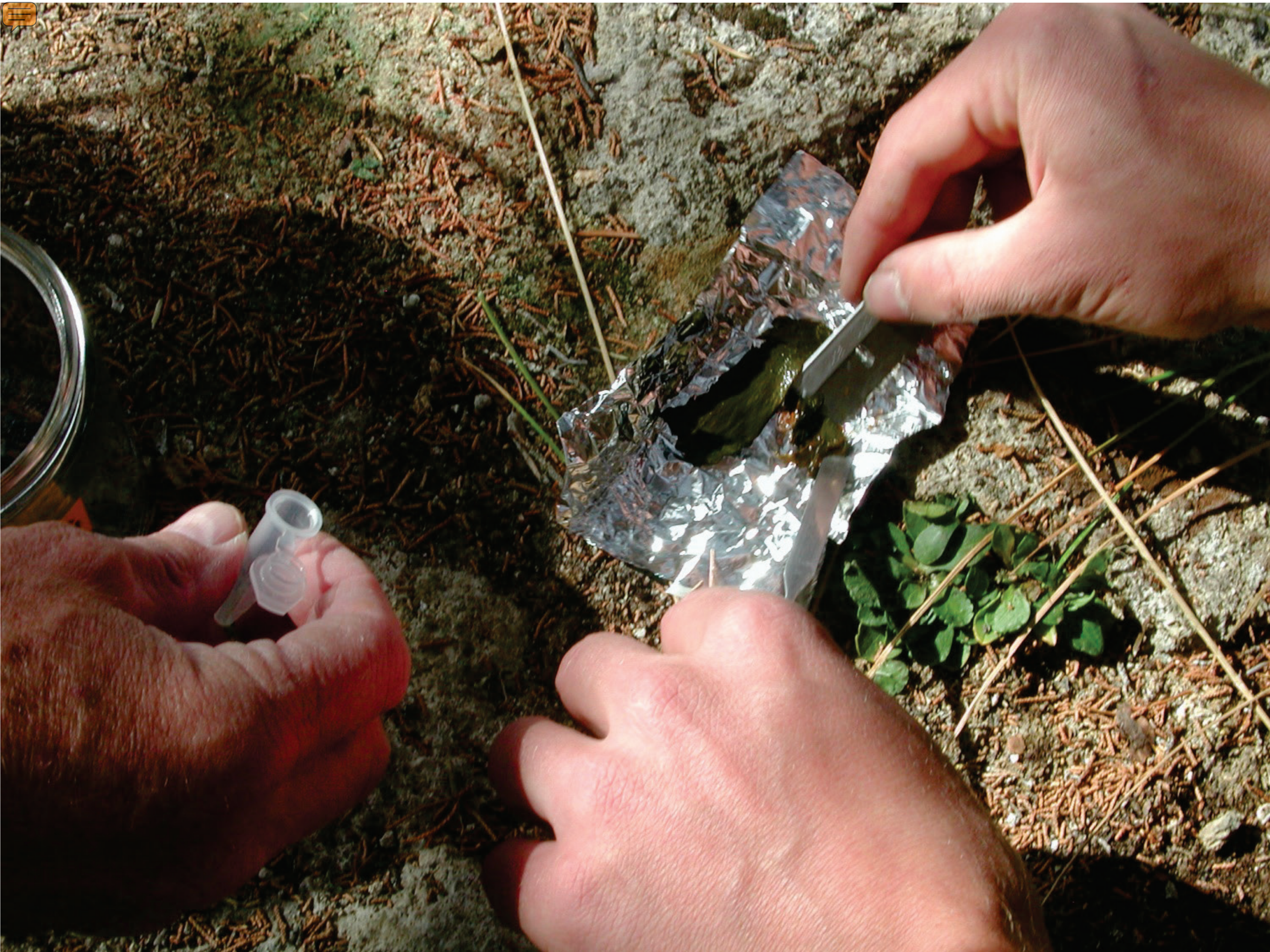

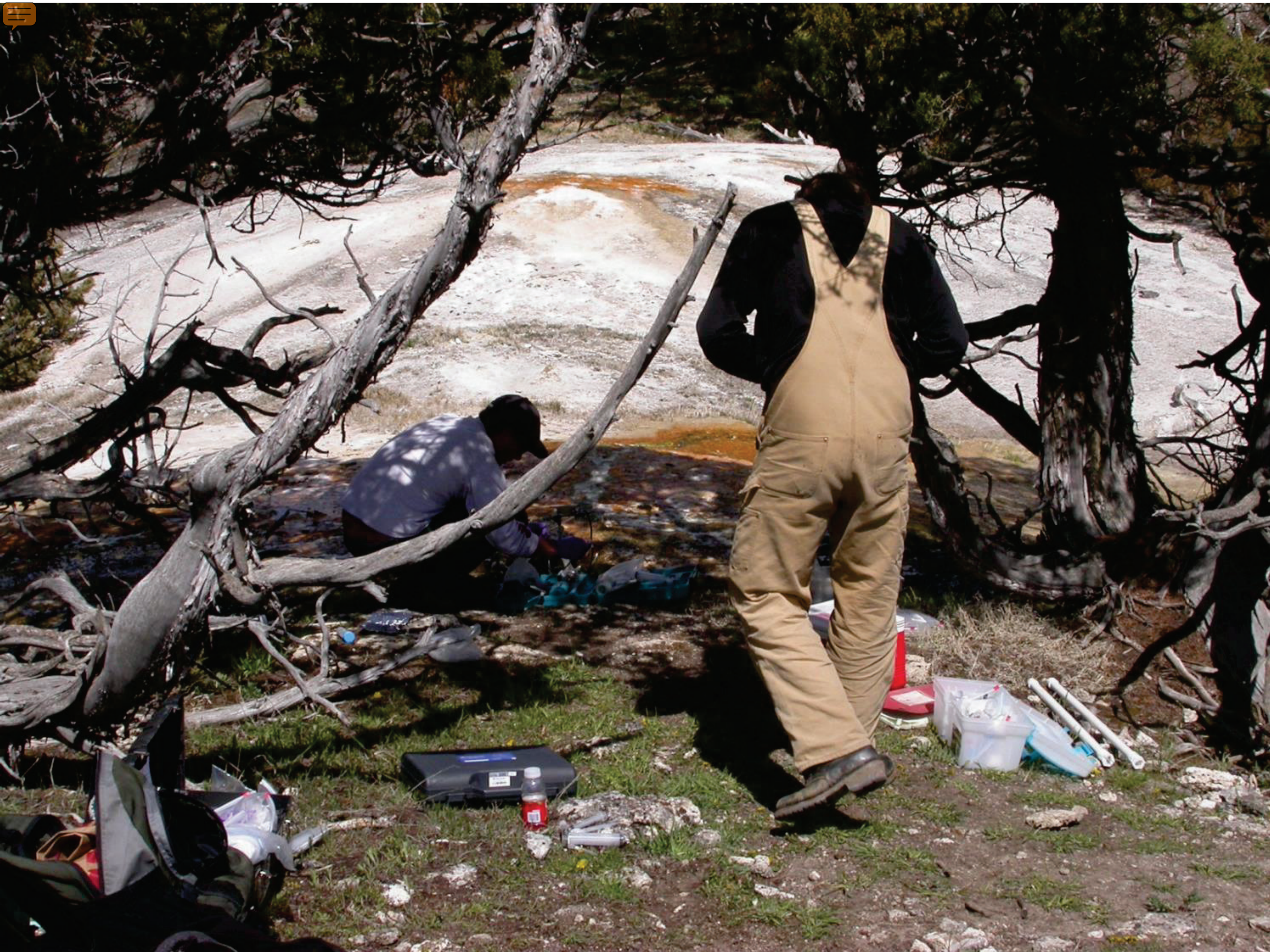

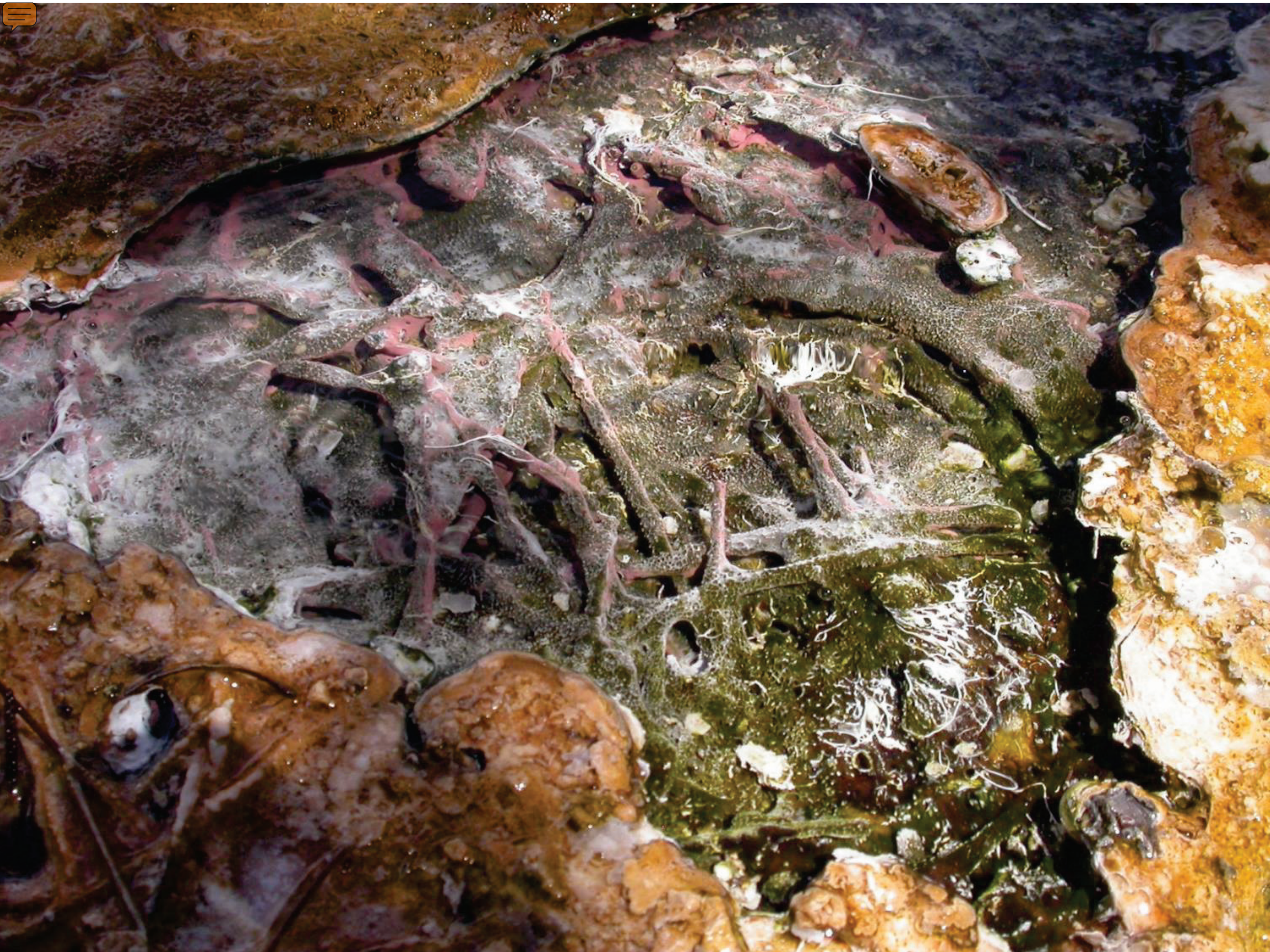

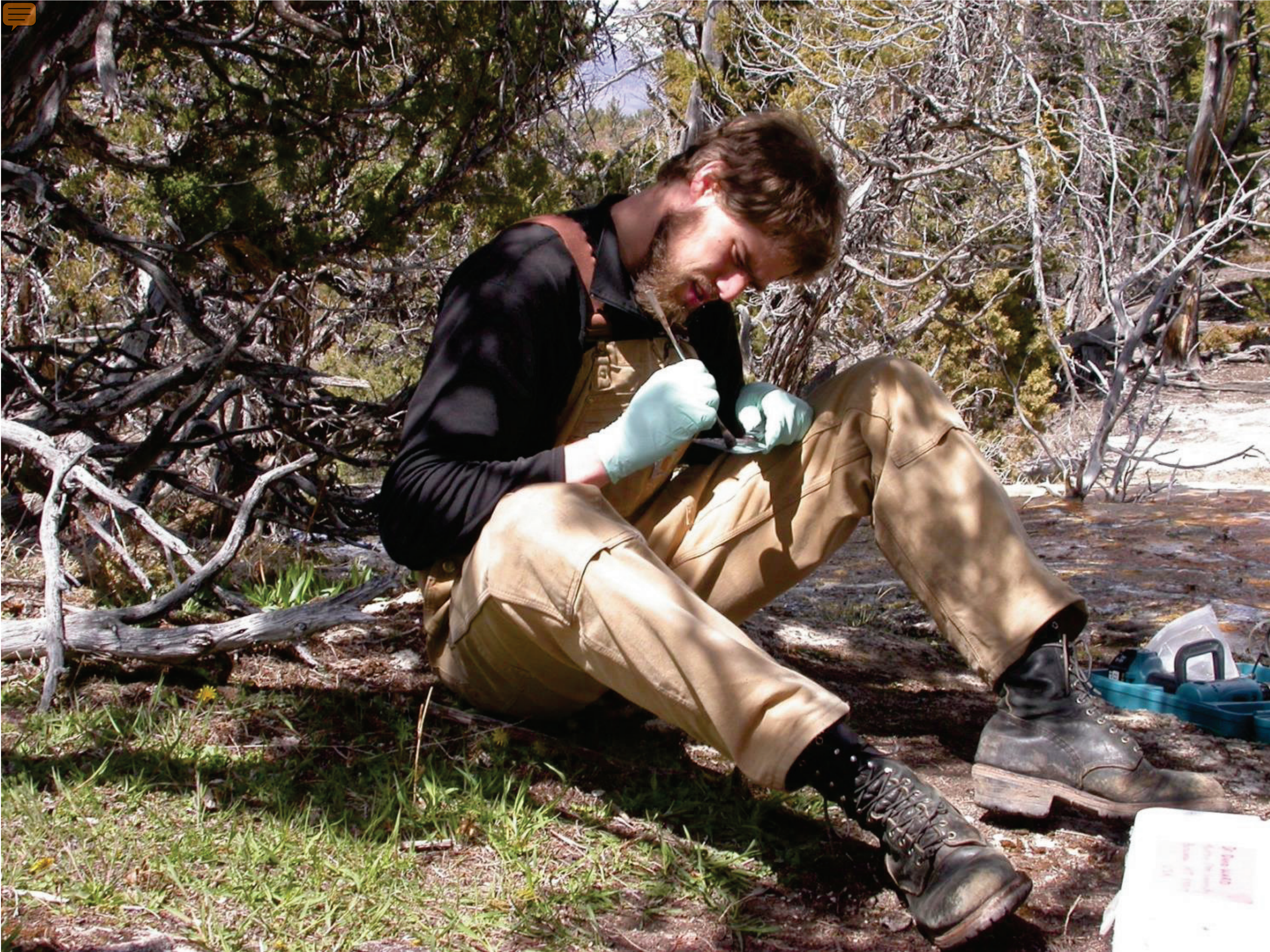

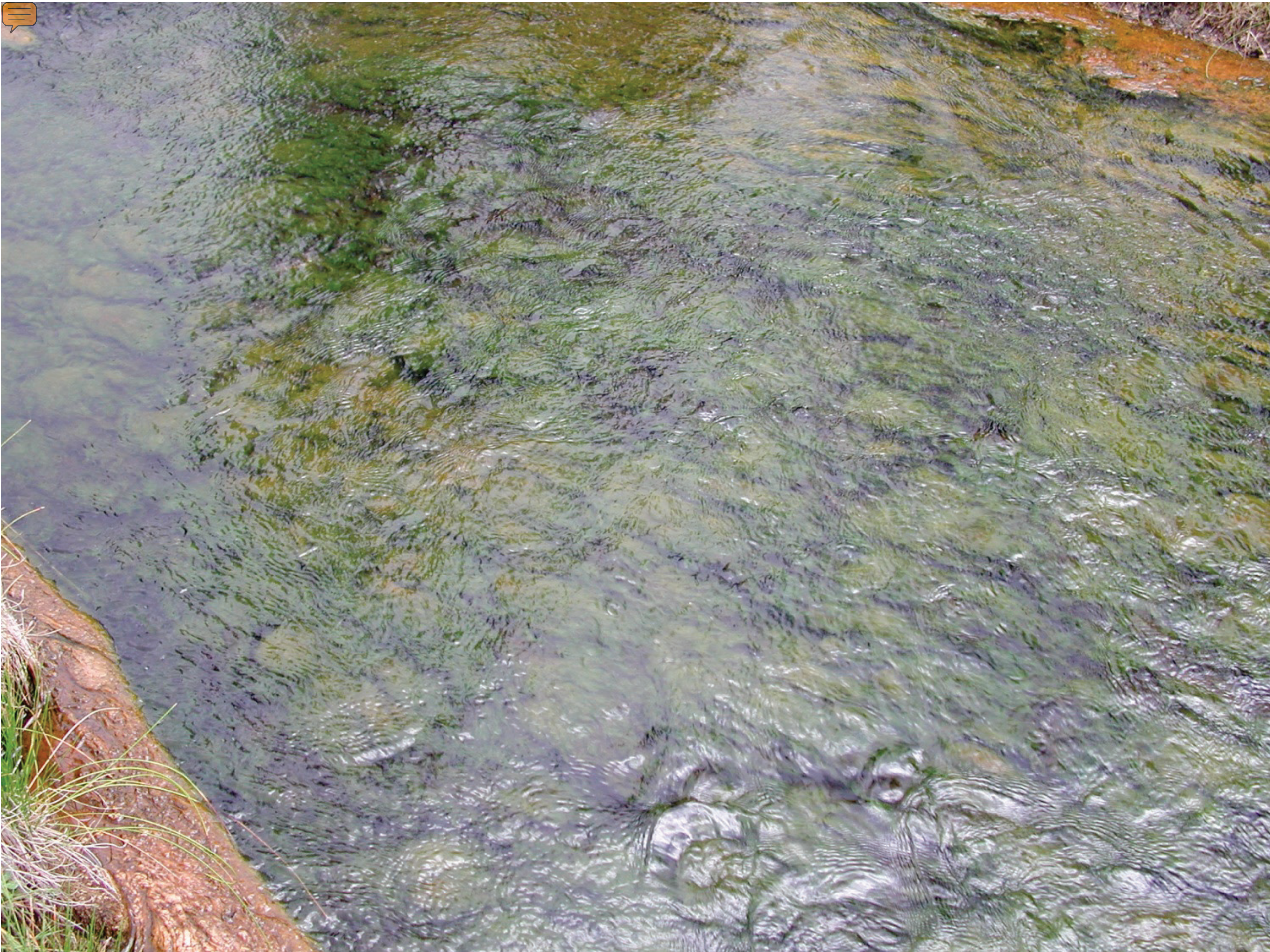

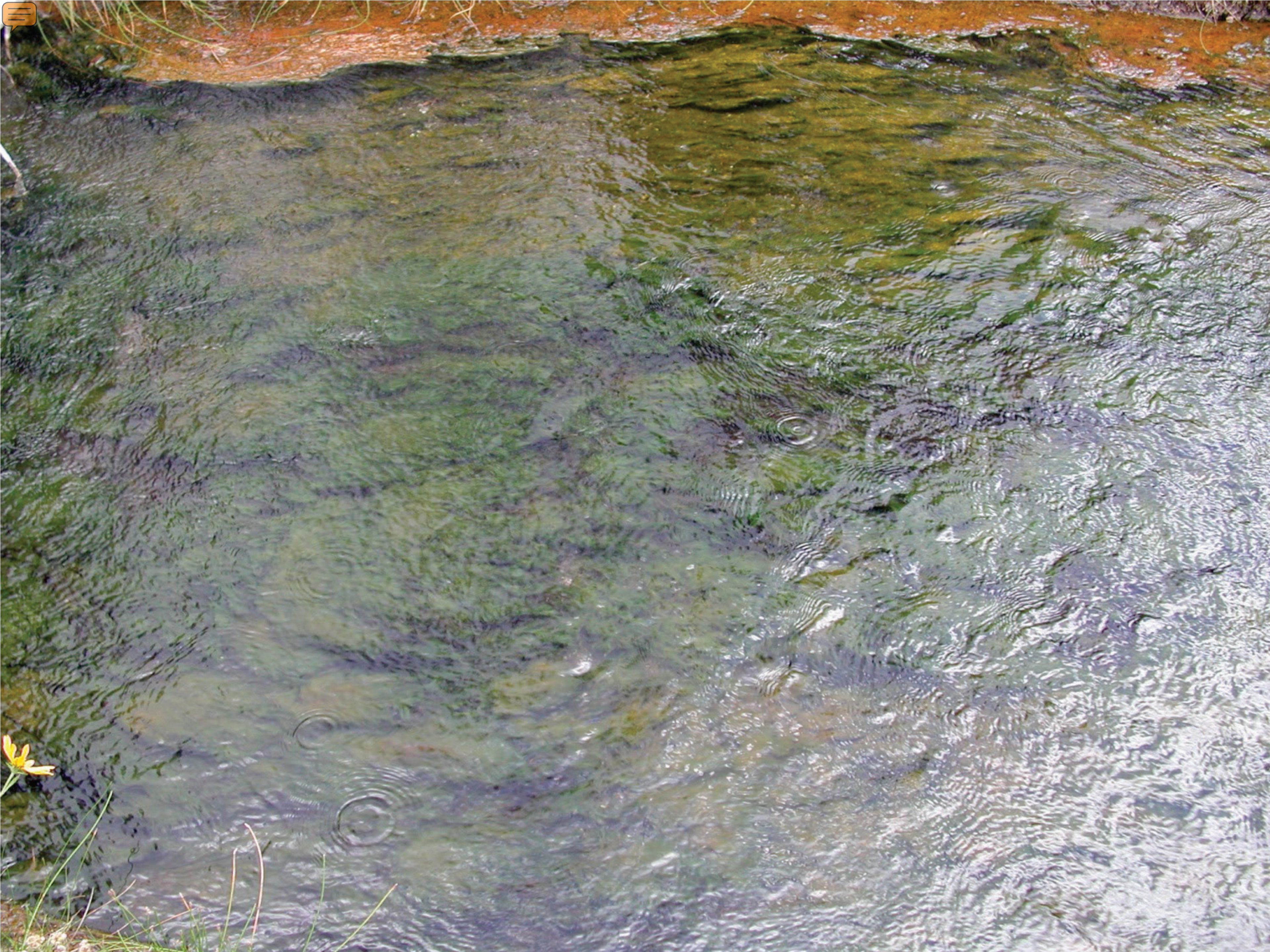

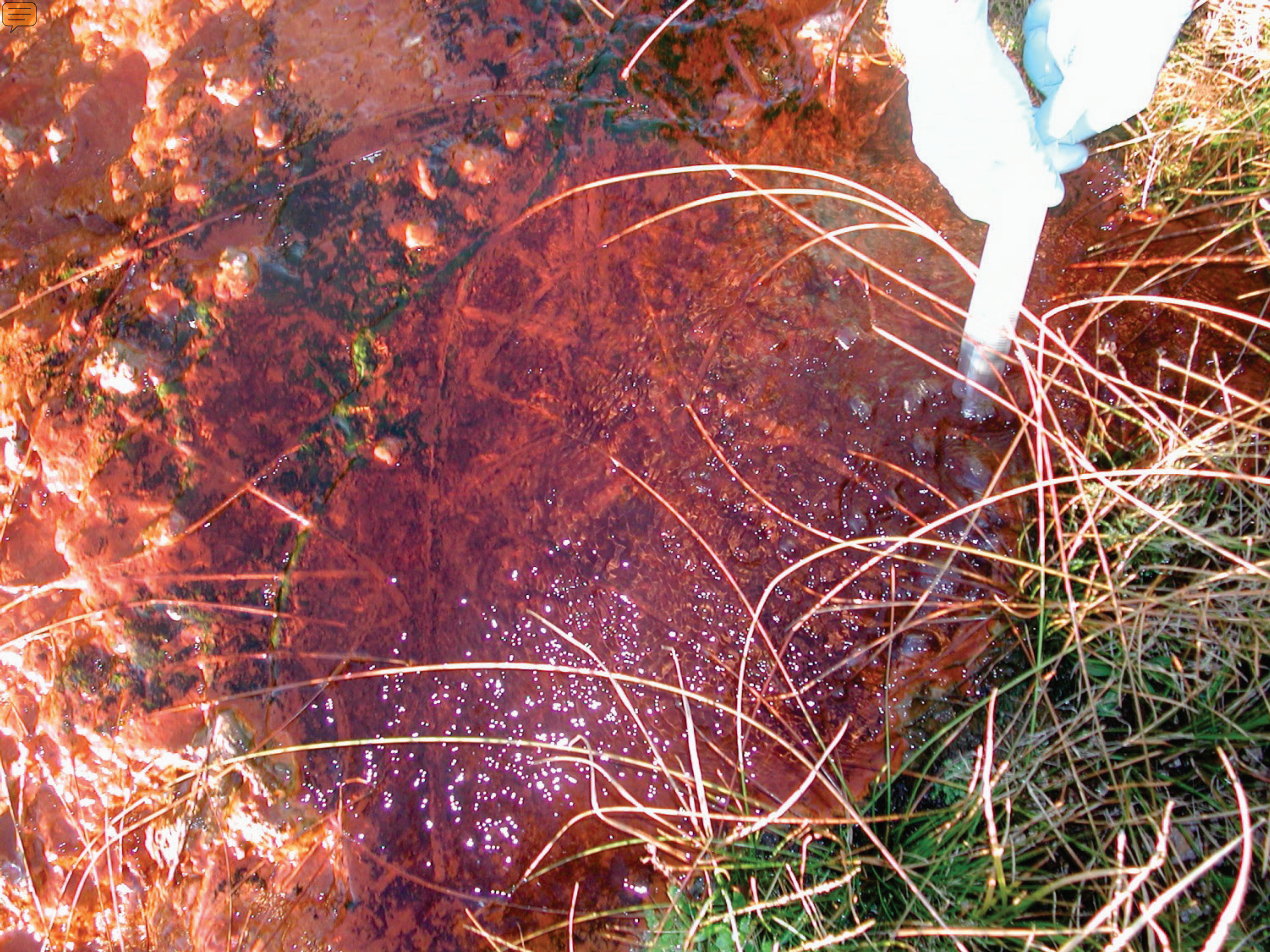

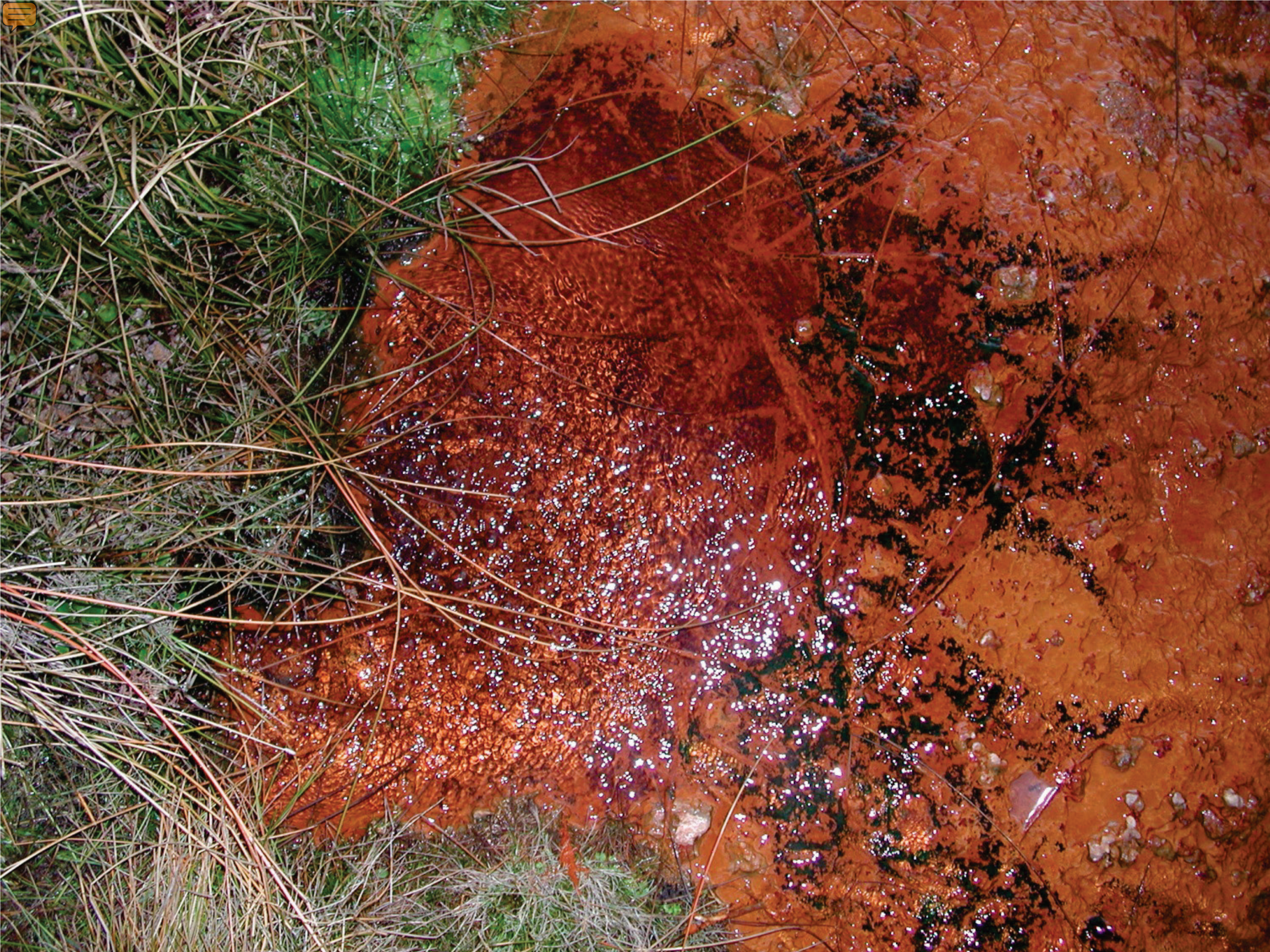

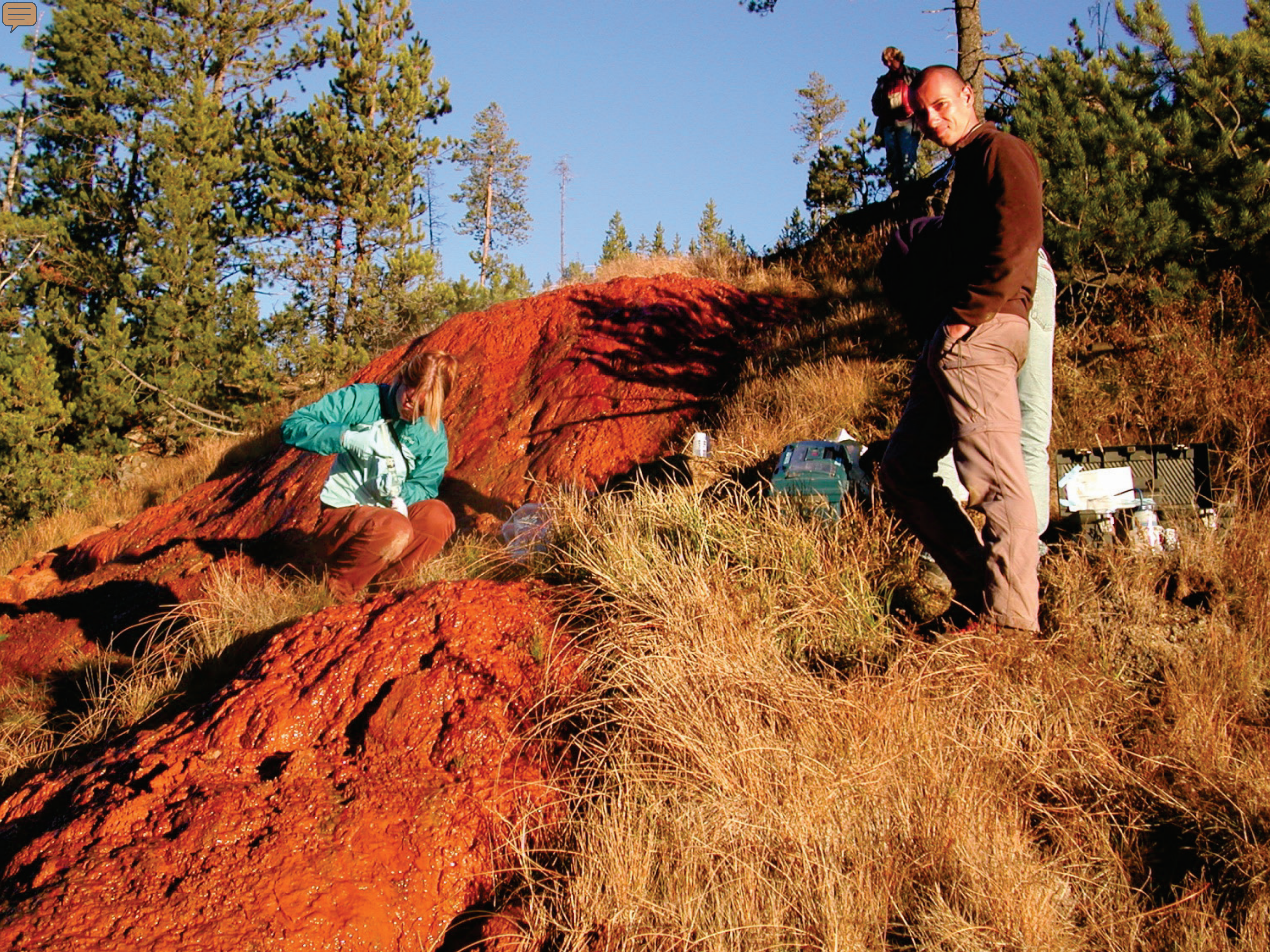

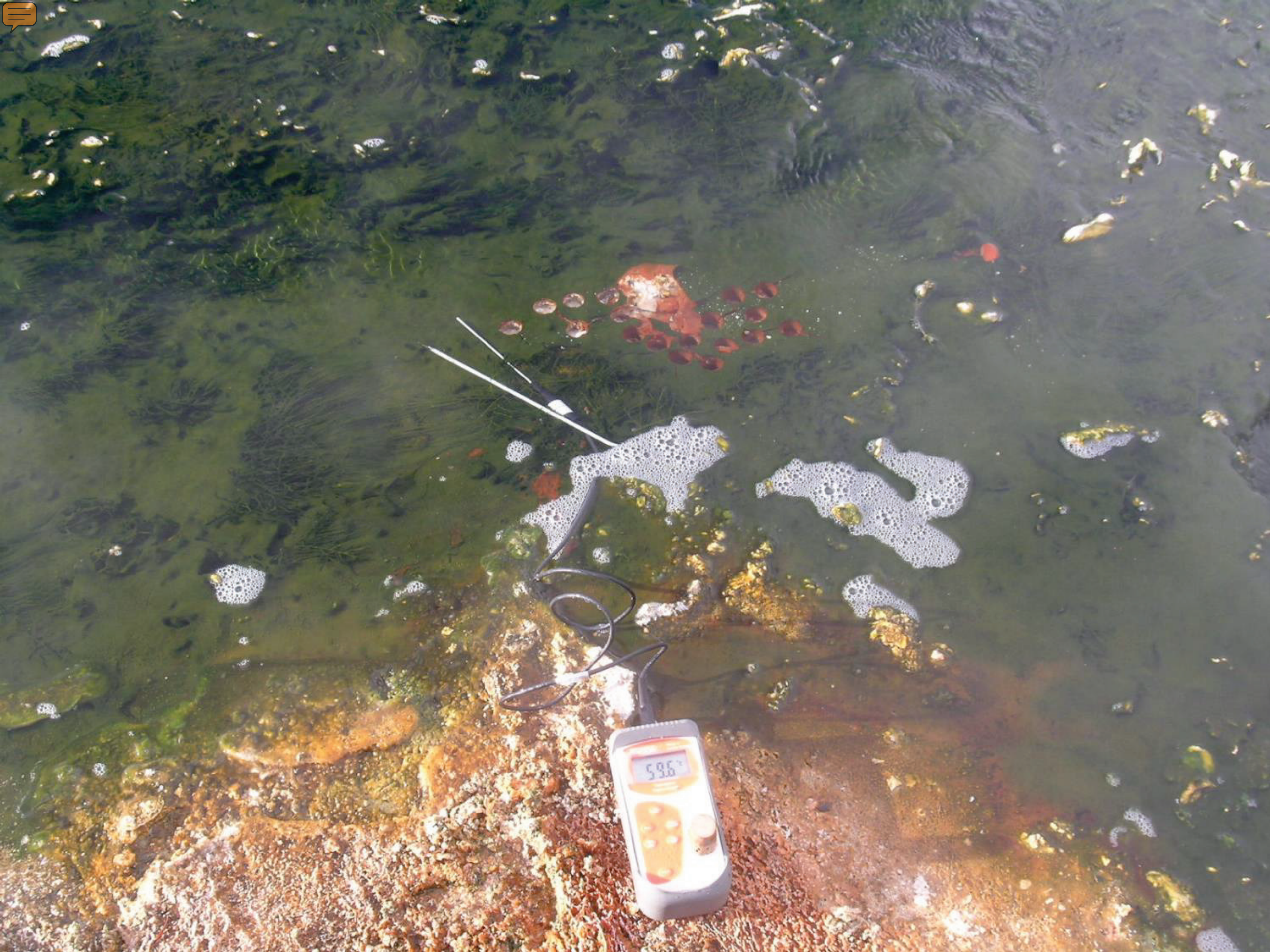
59.6

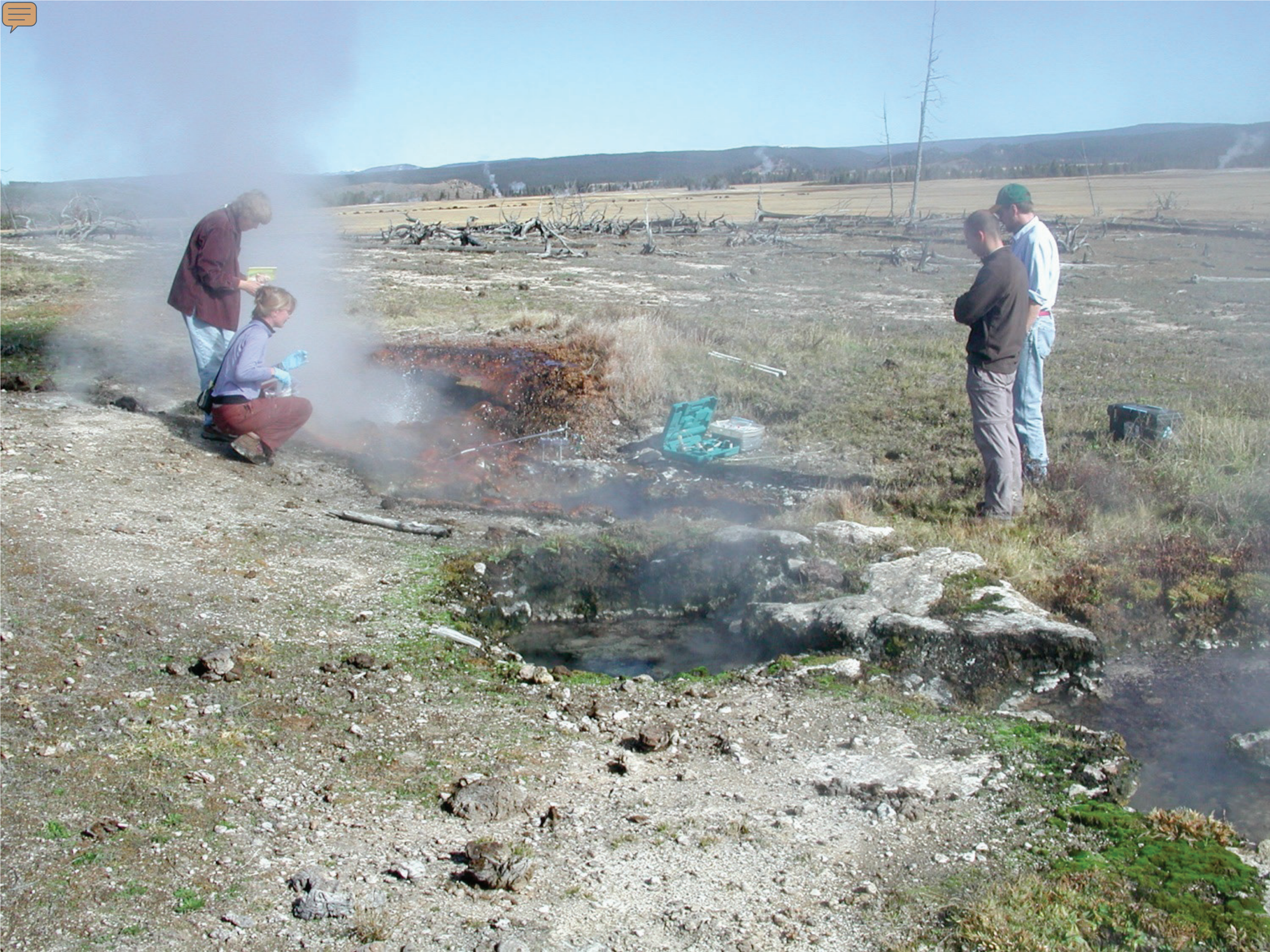

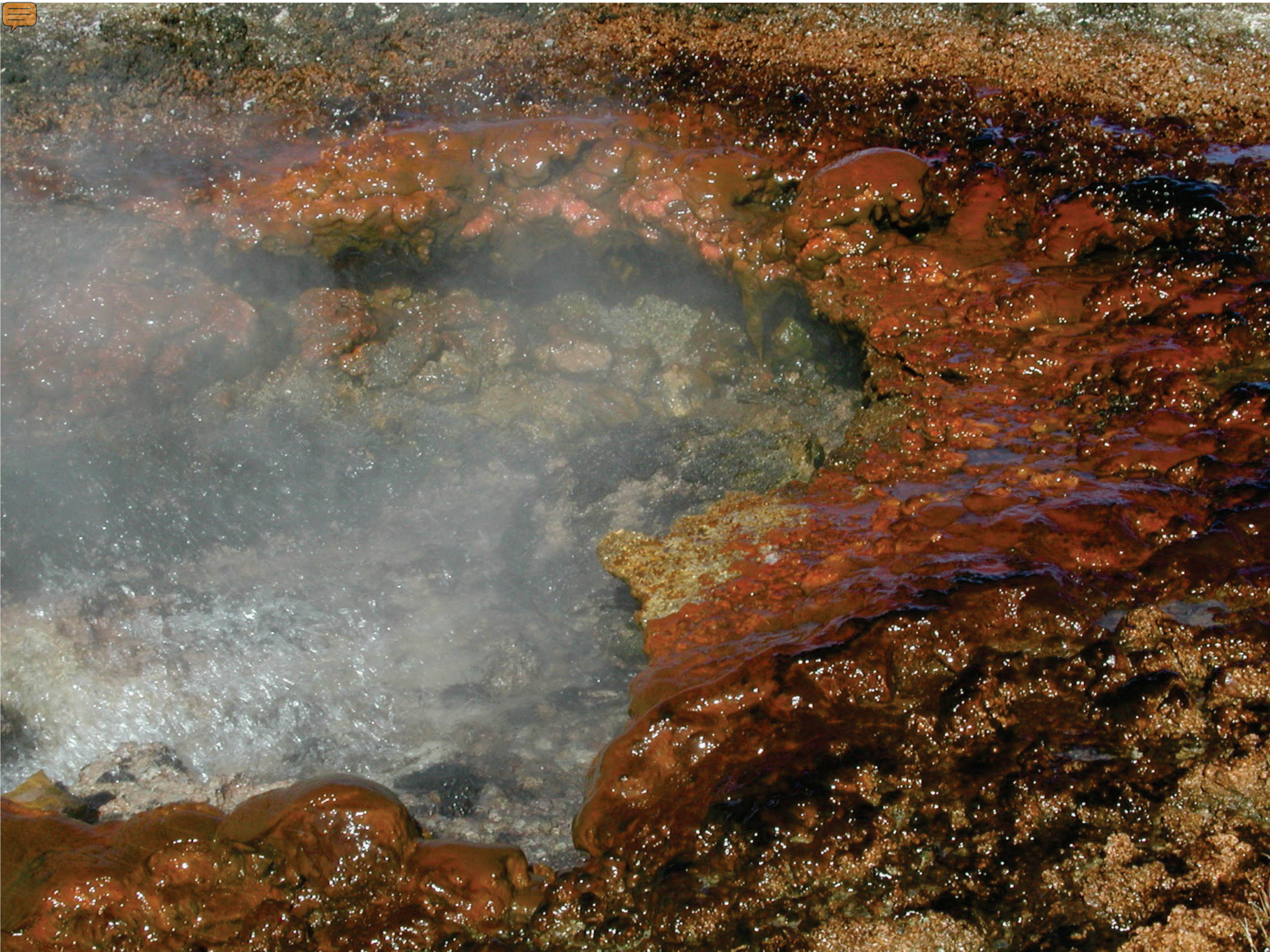

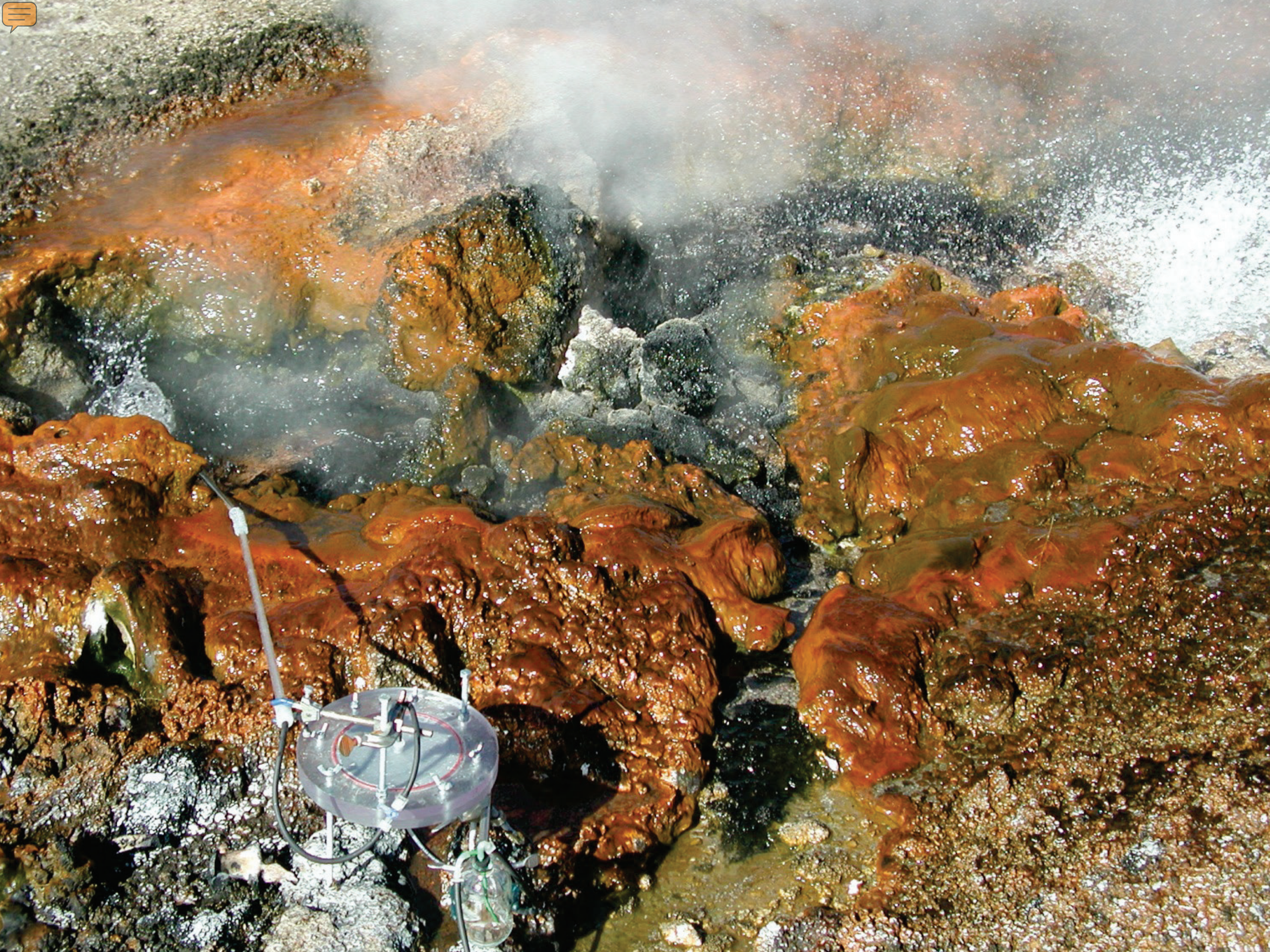

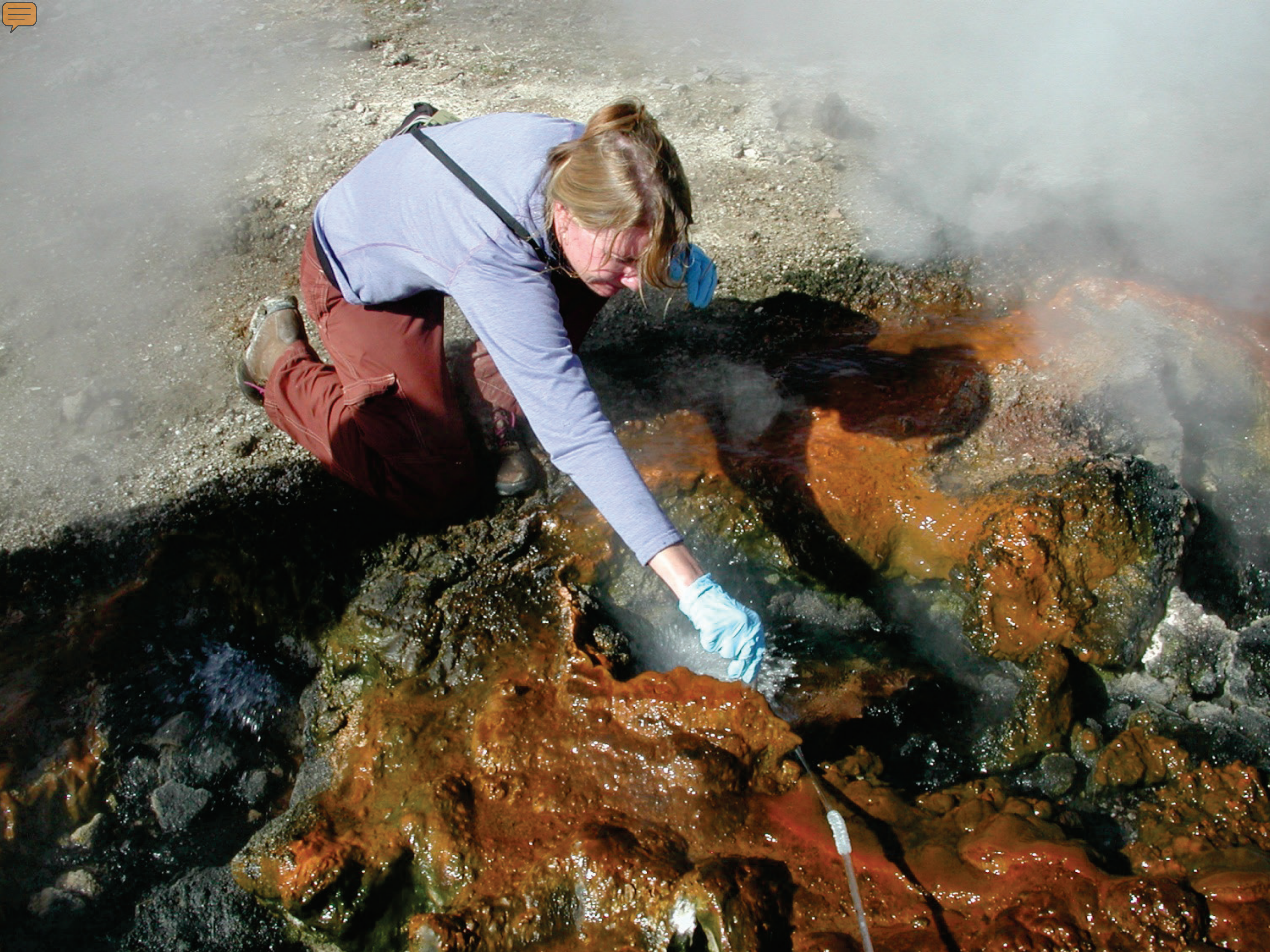

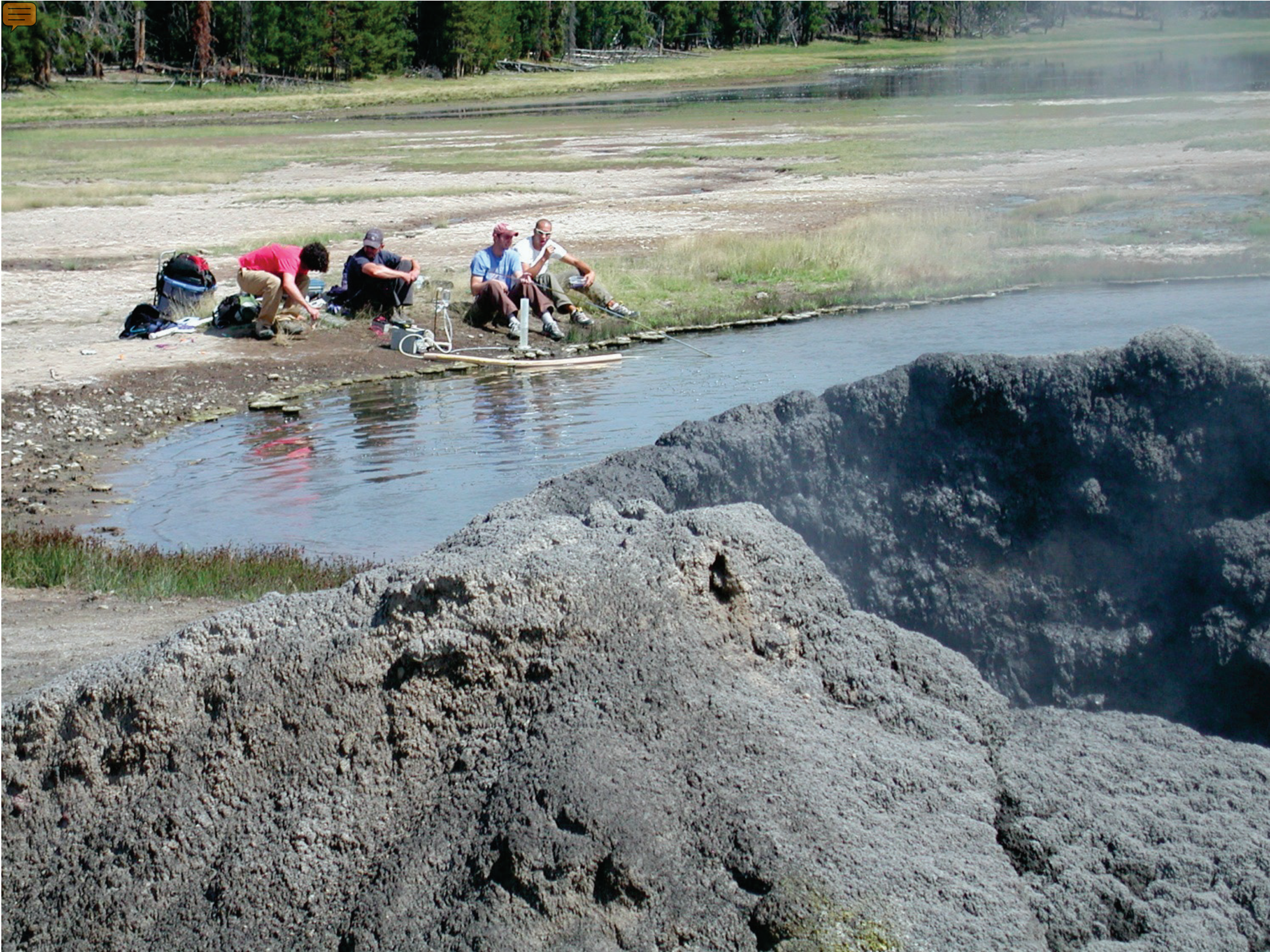

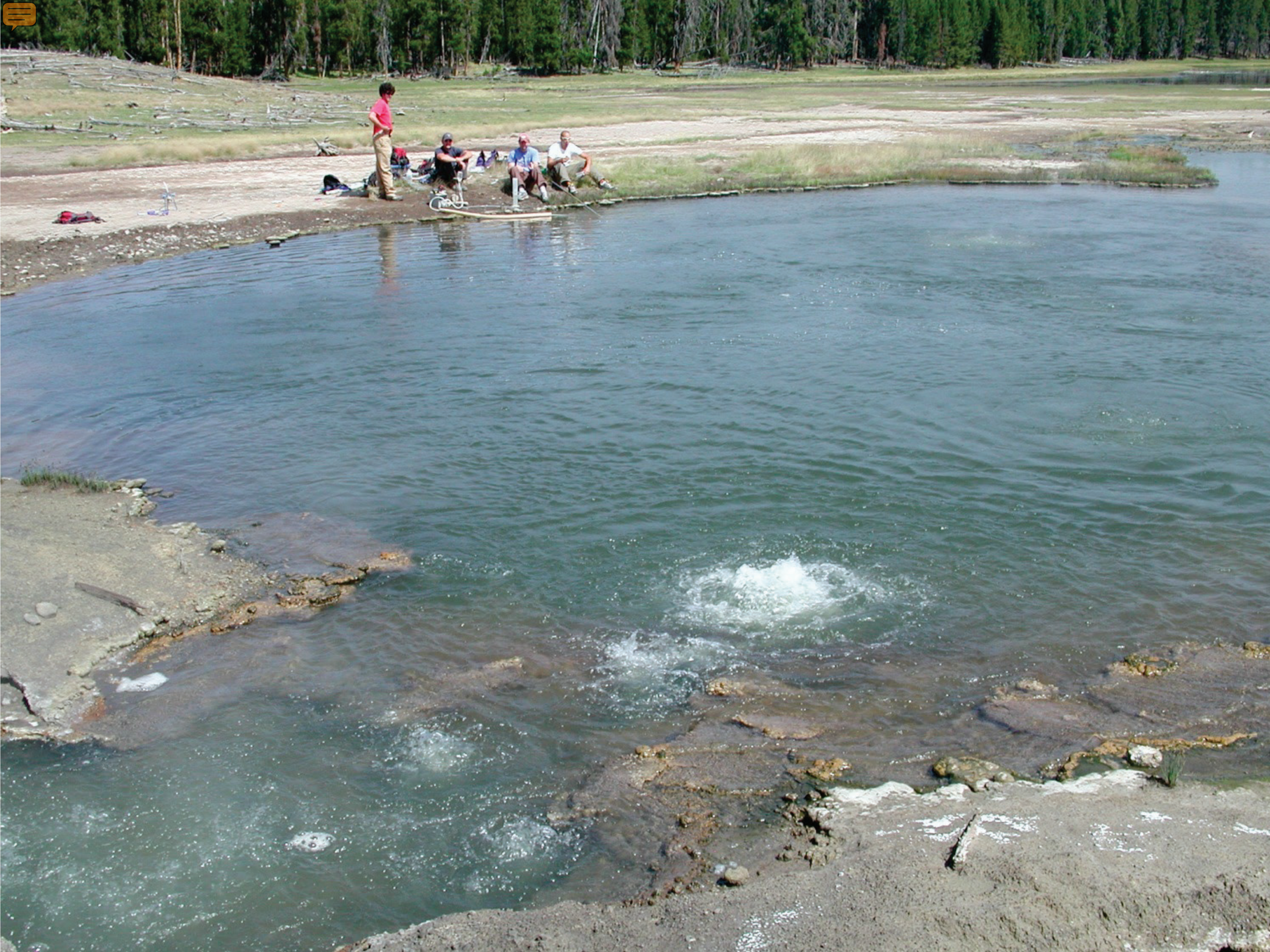

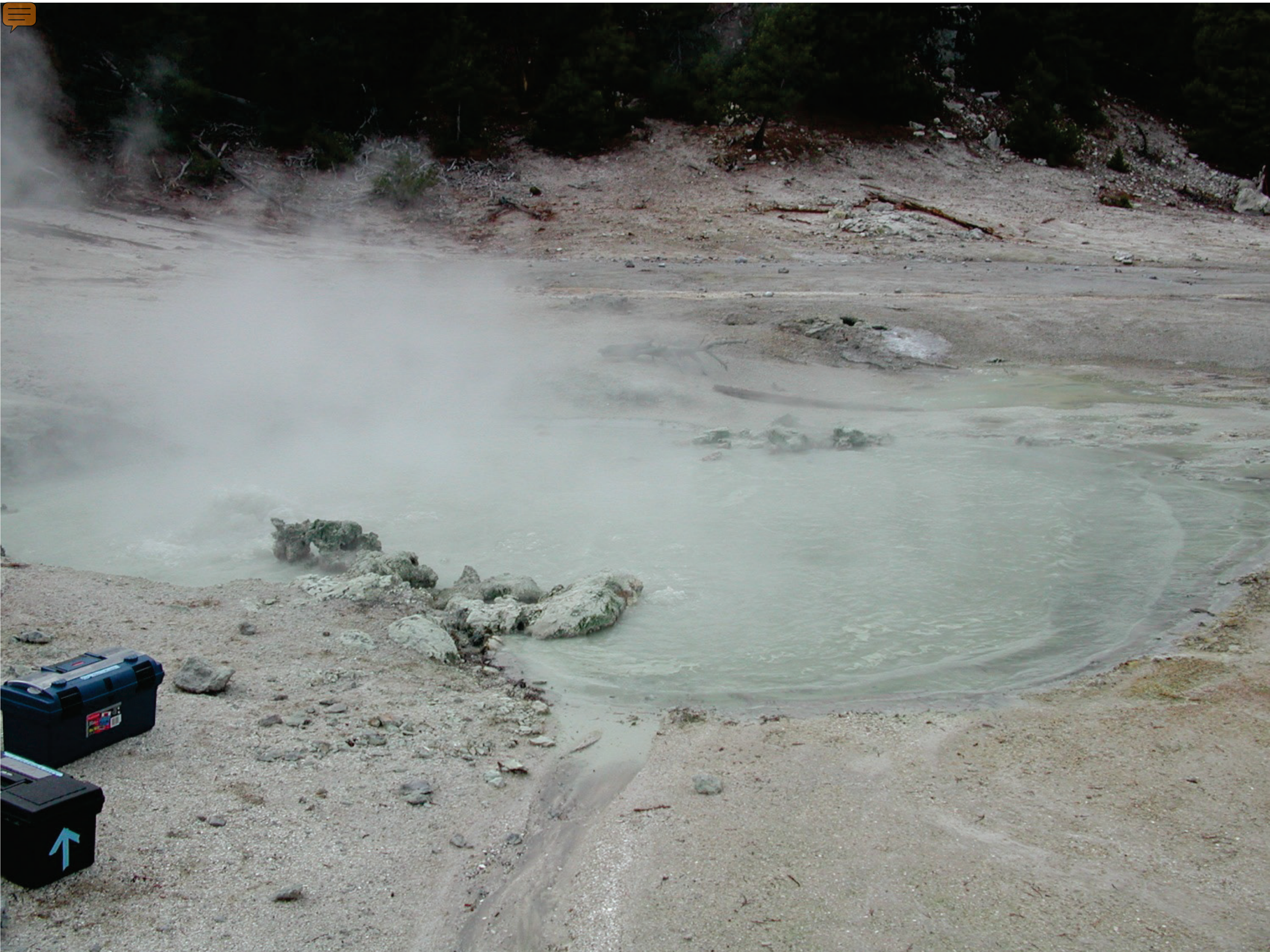

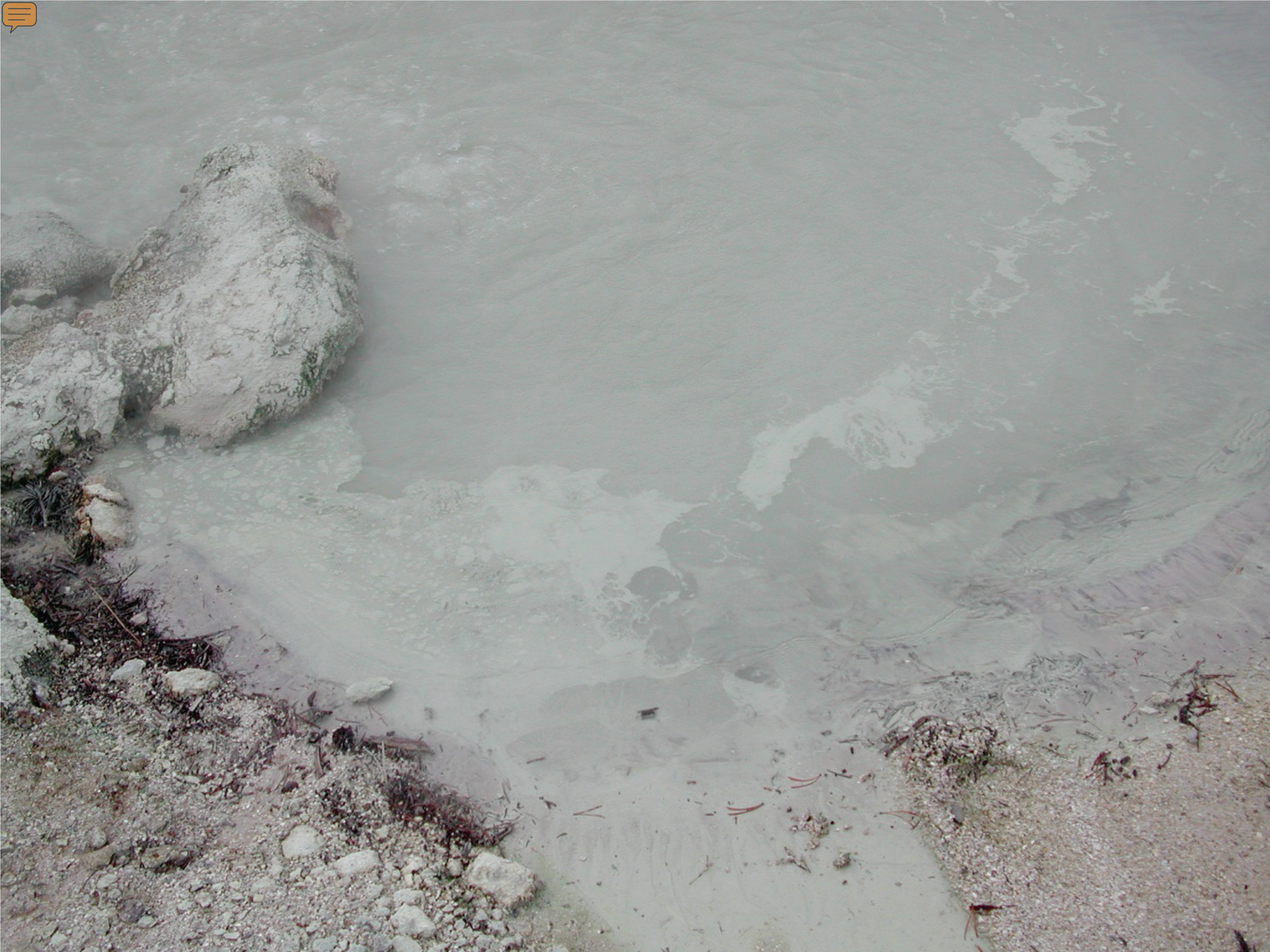

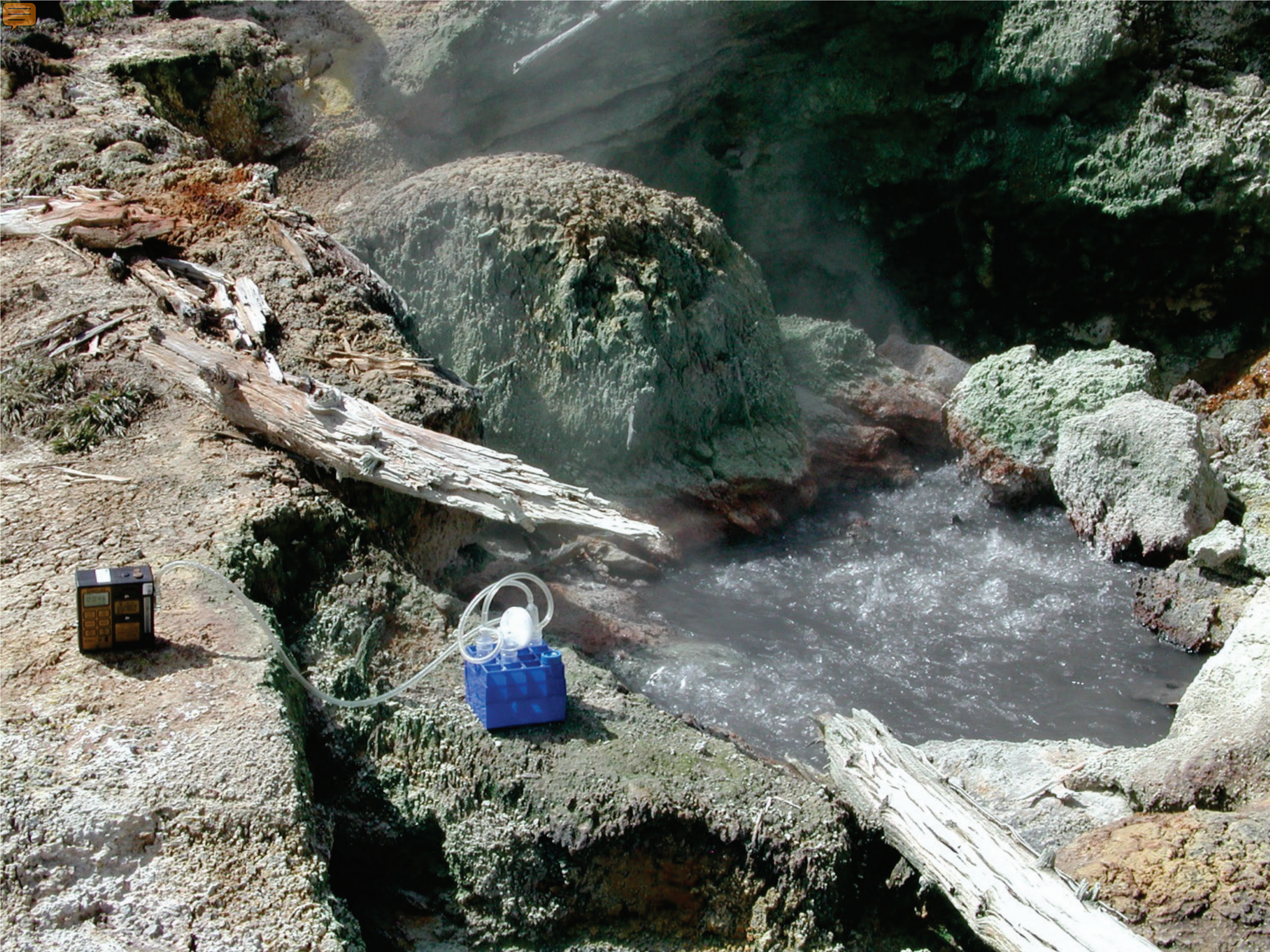

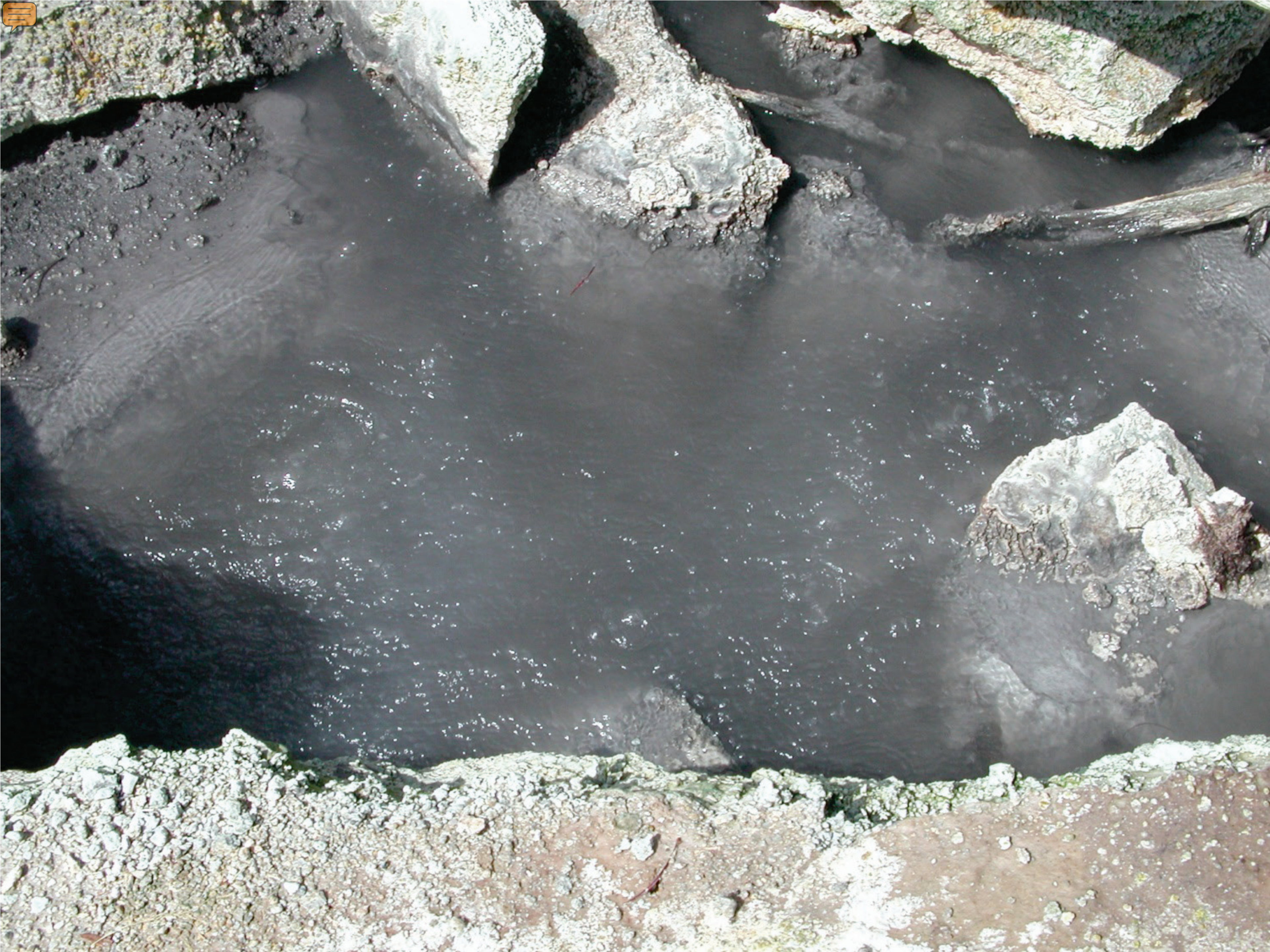

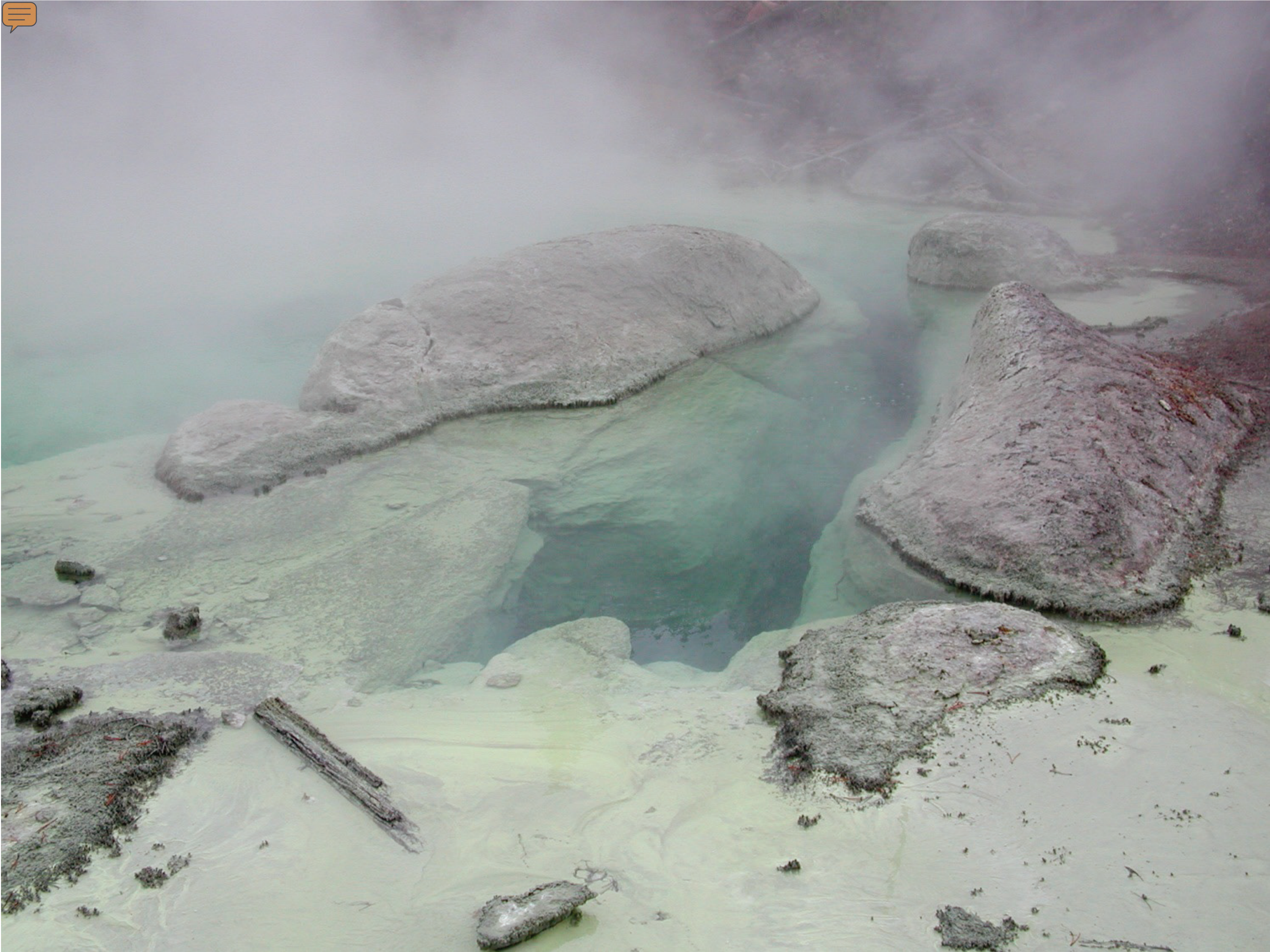

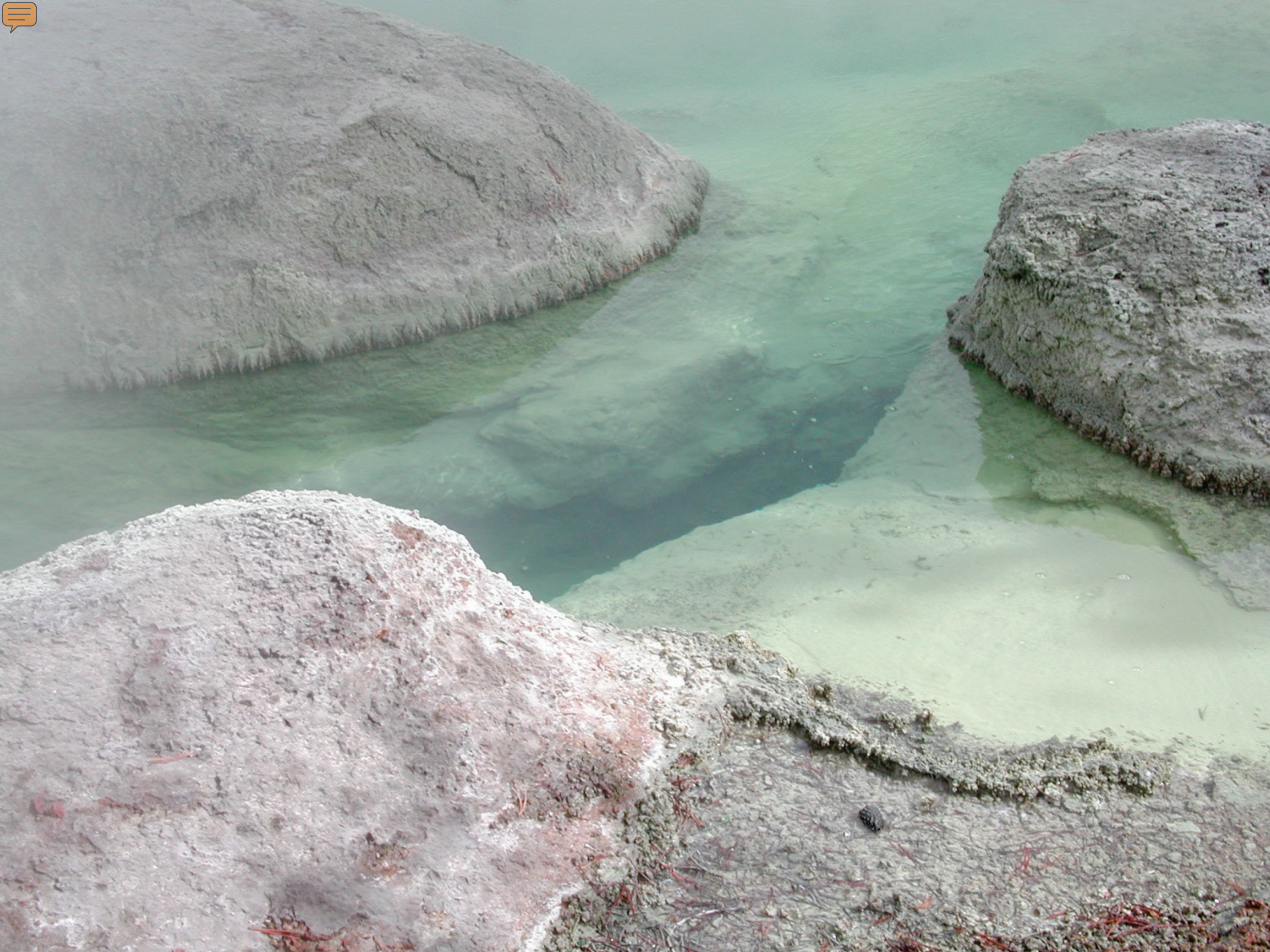

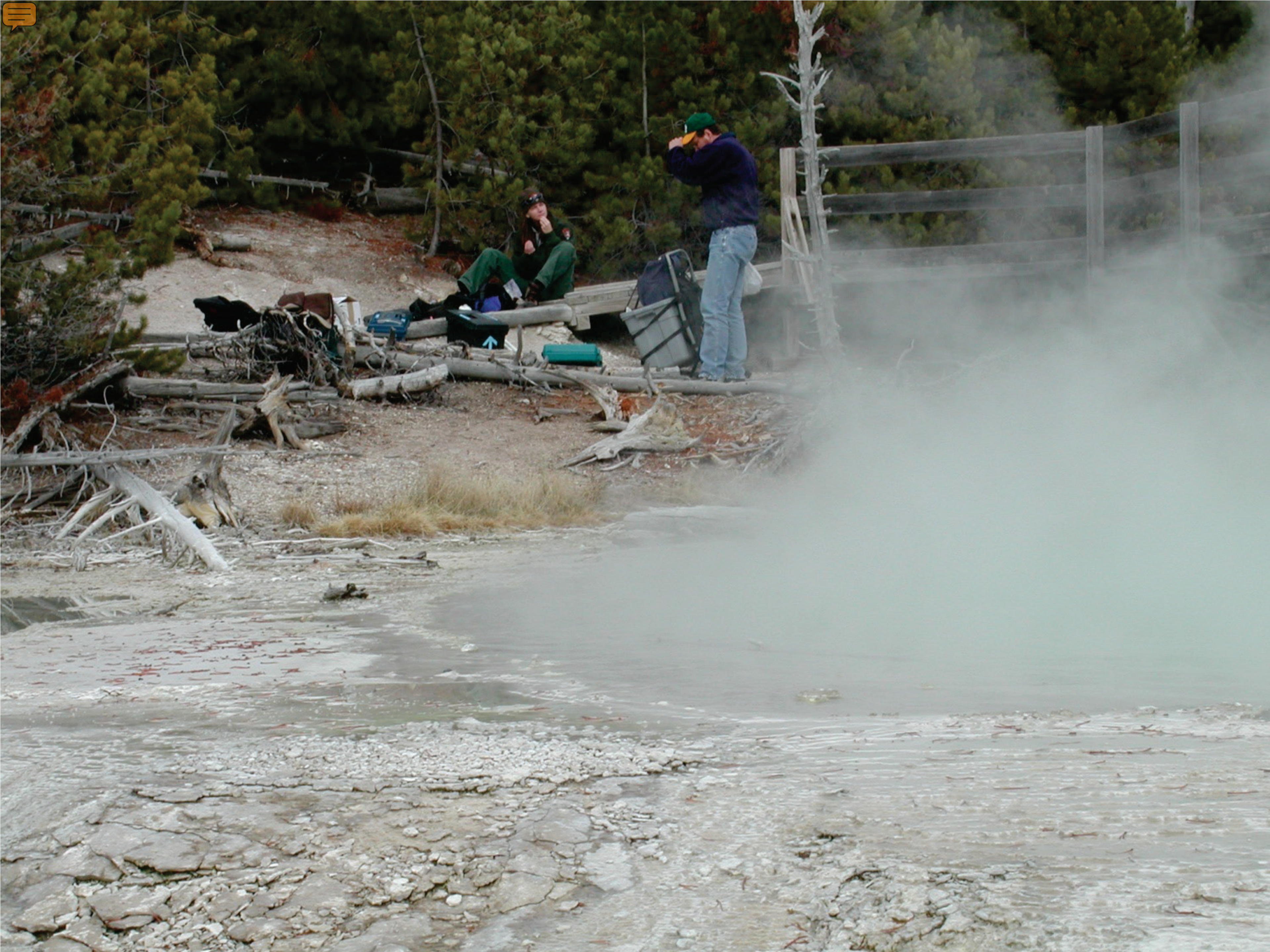

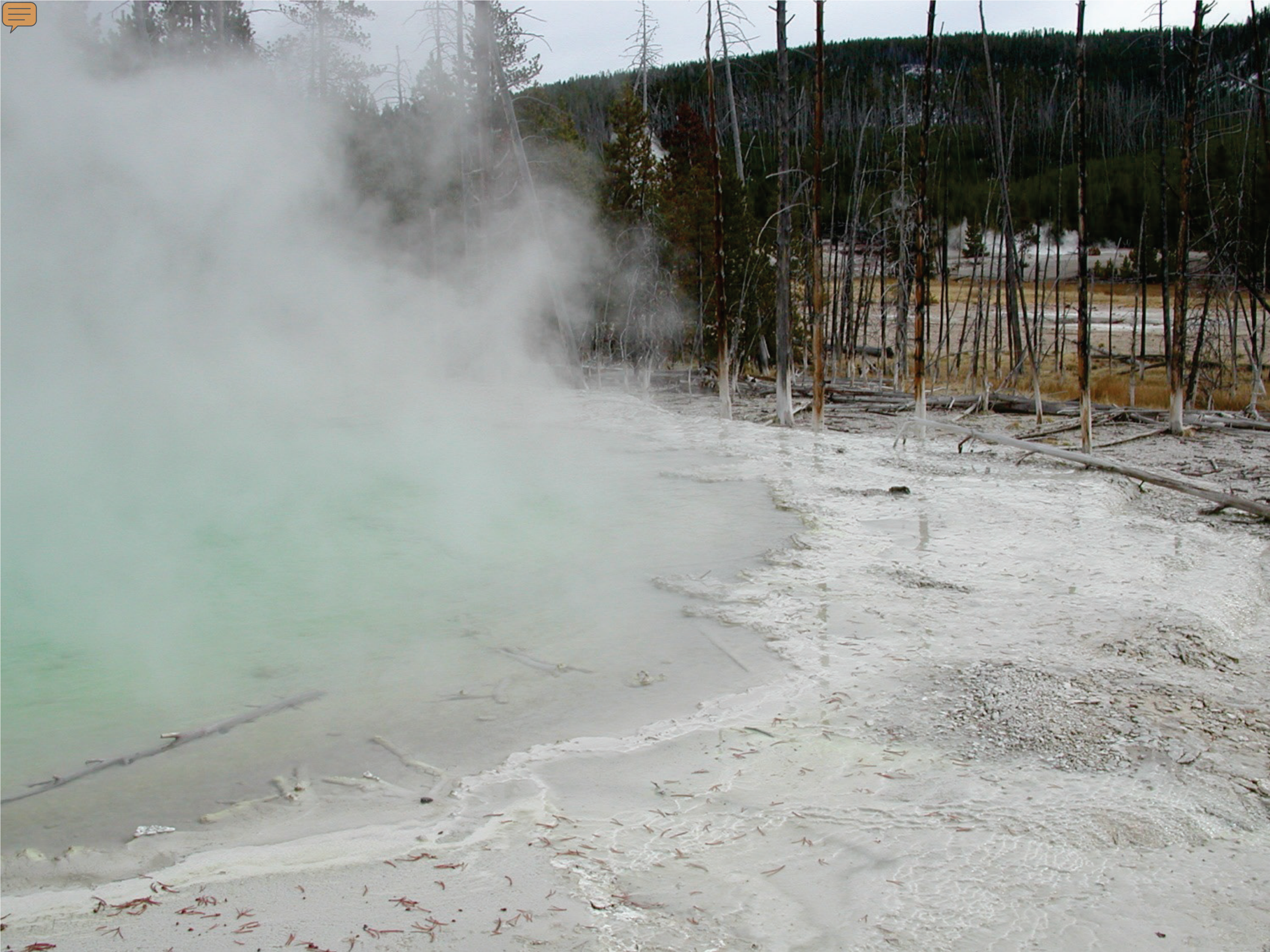

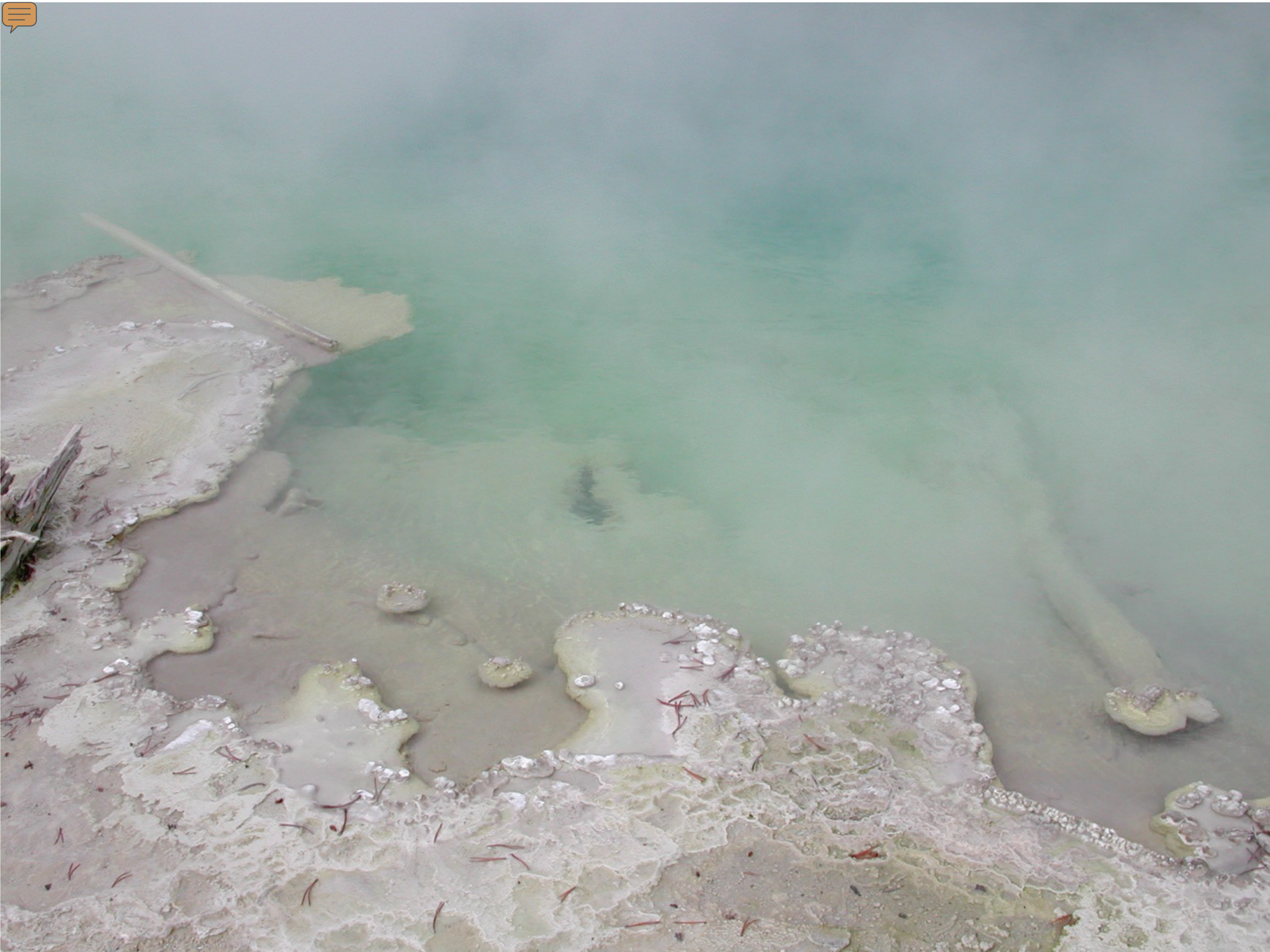

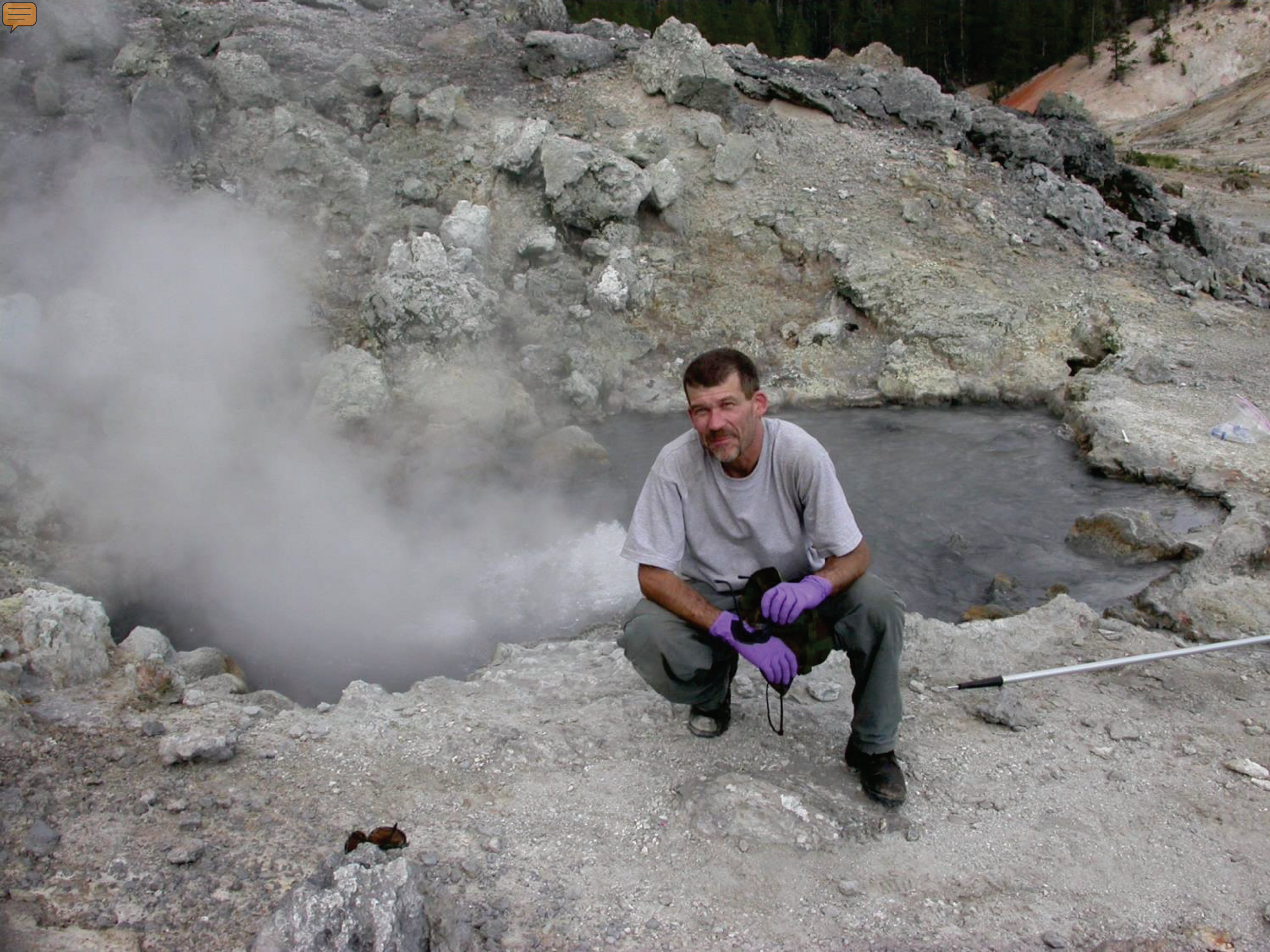

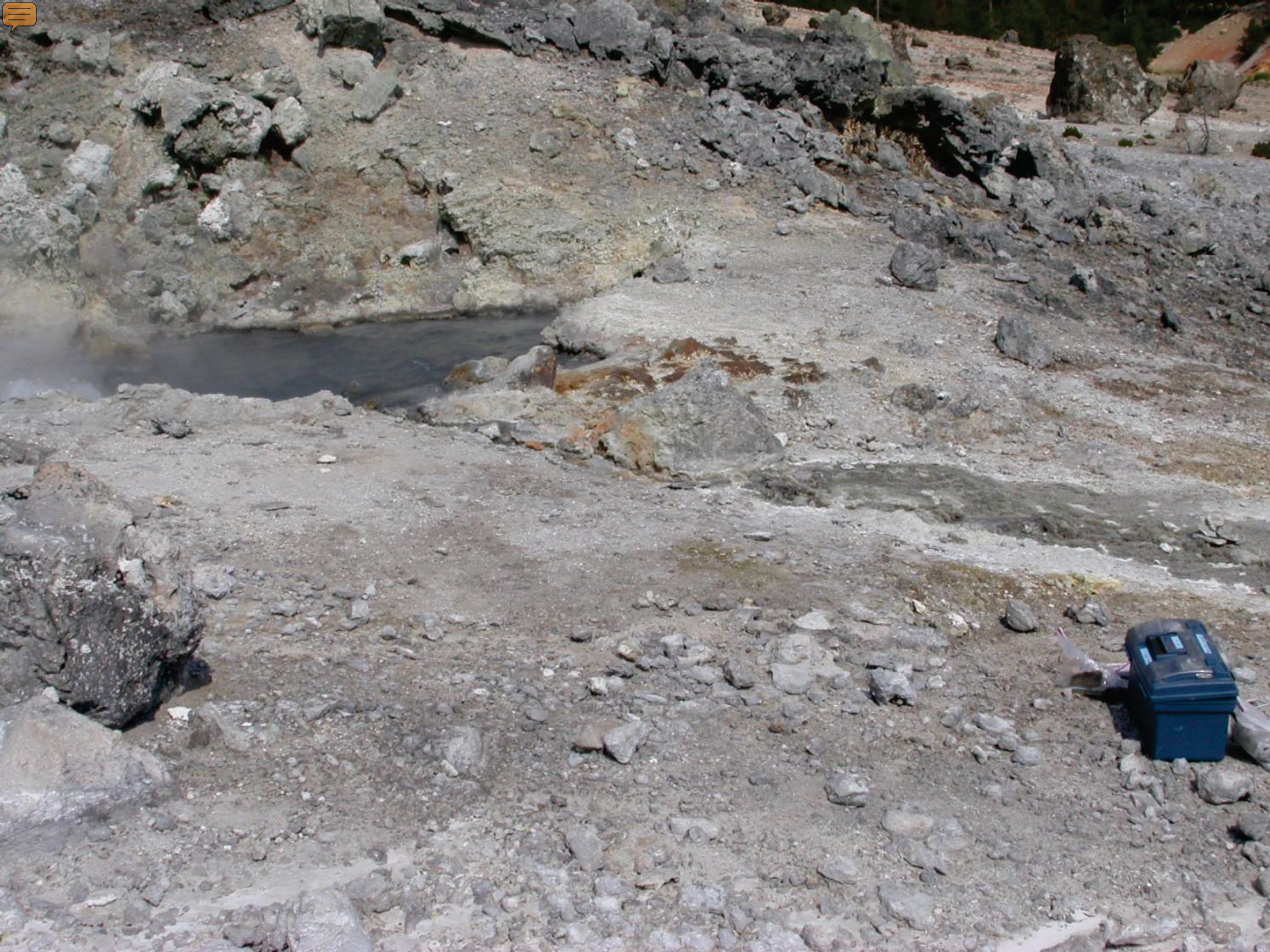

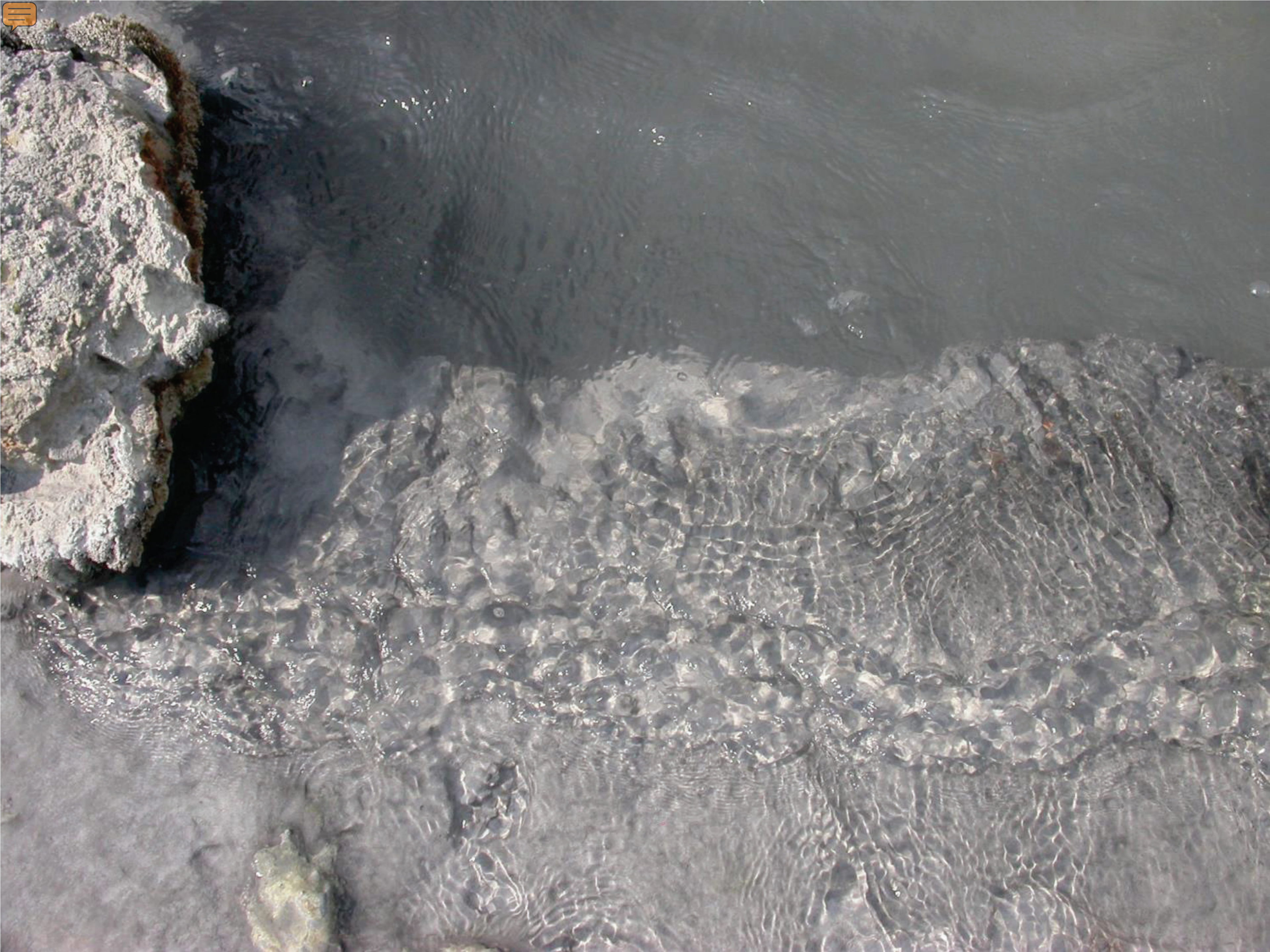

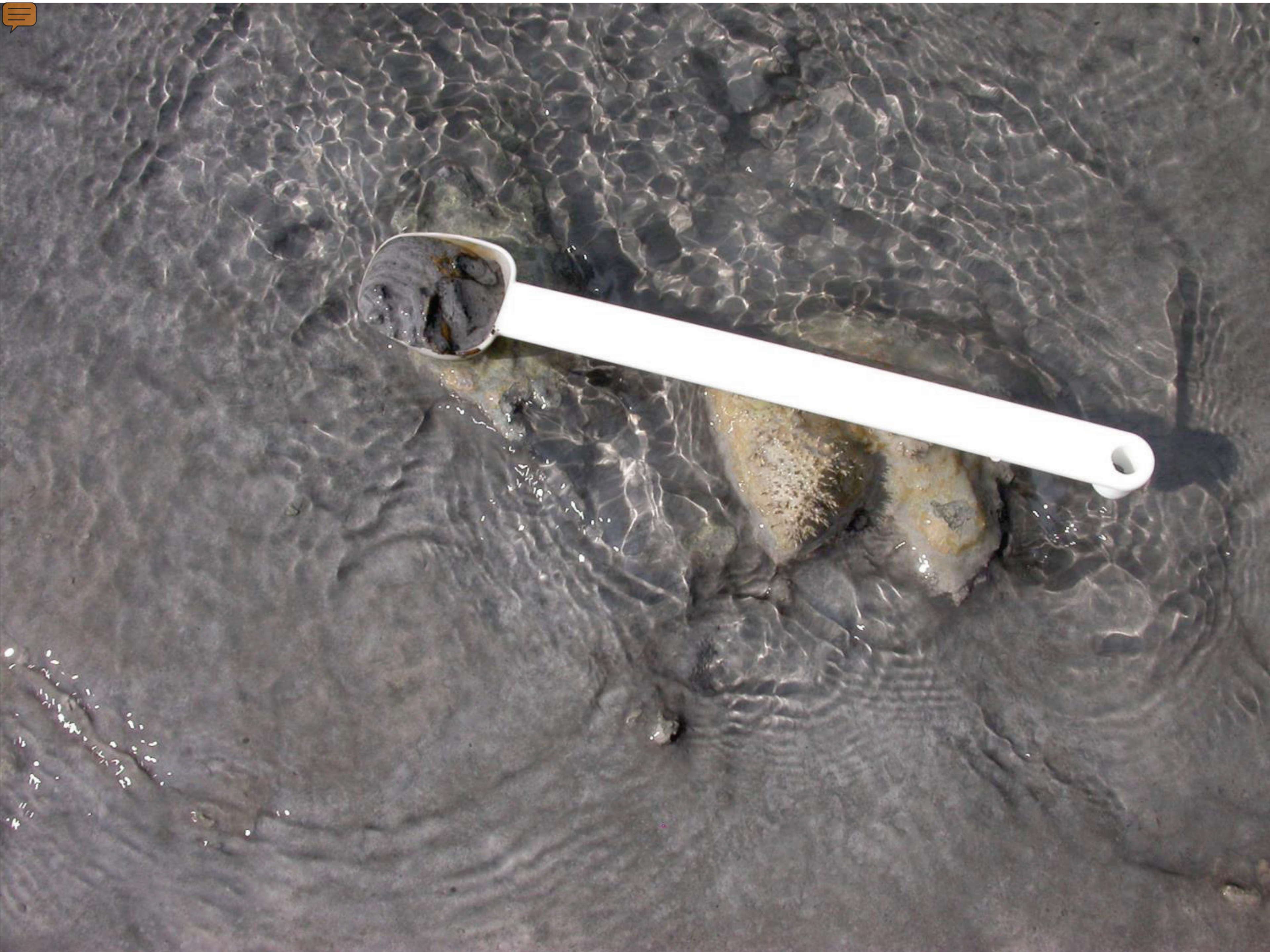

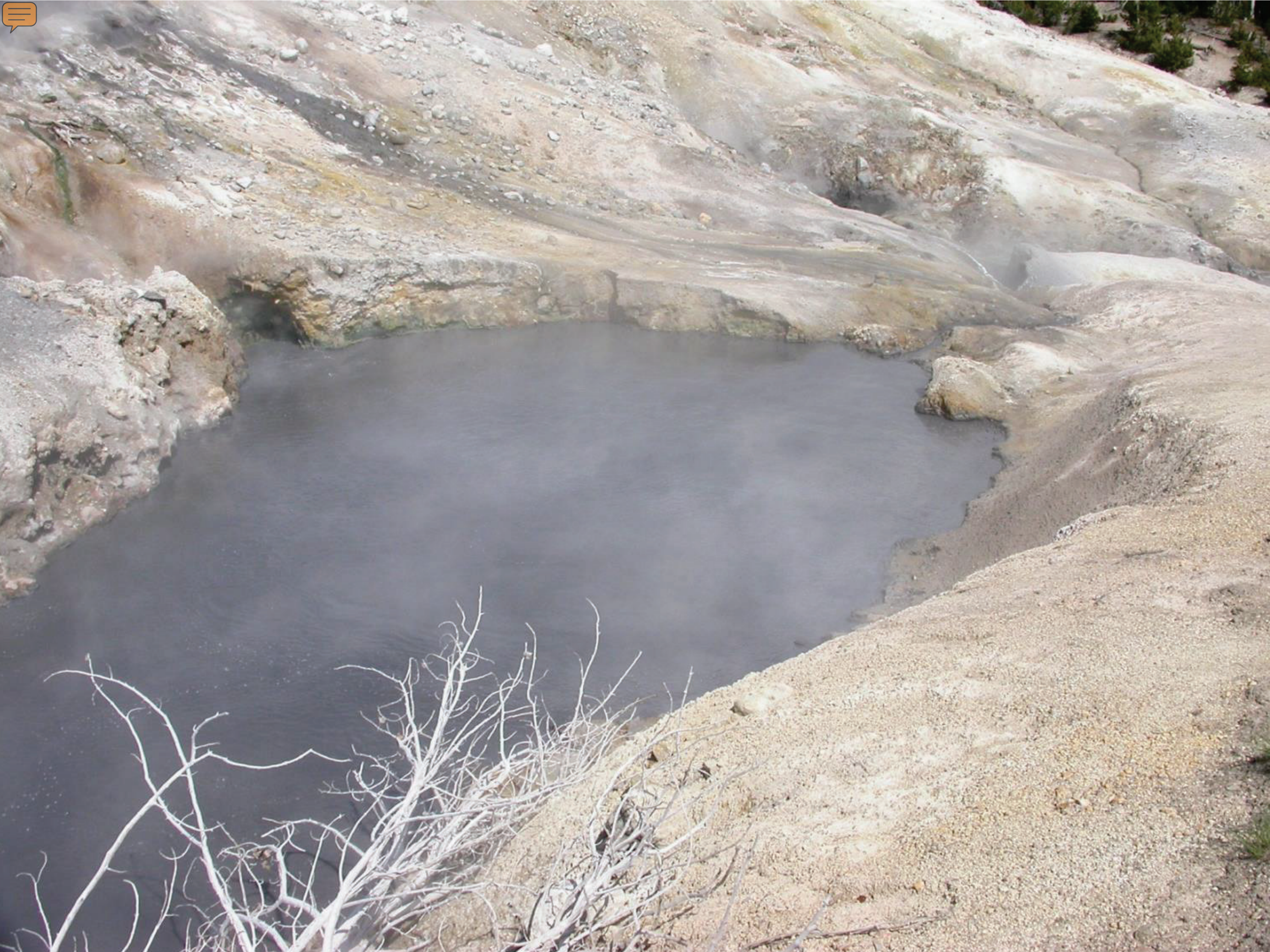

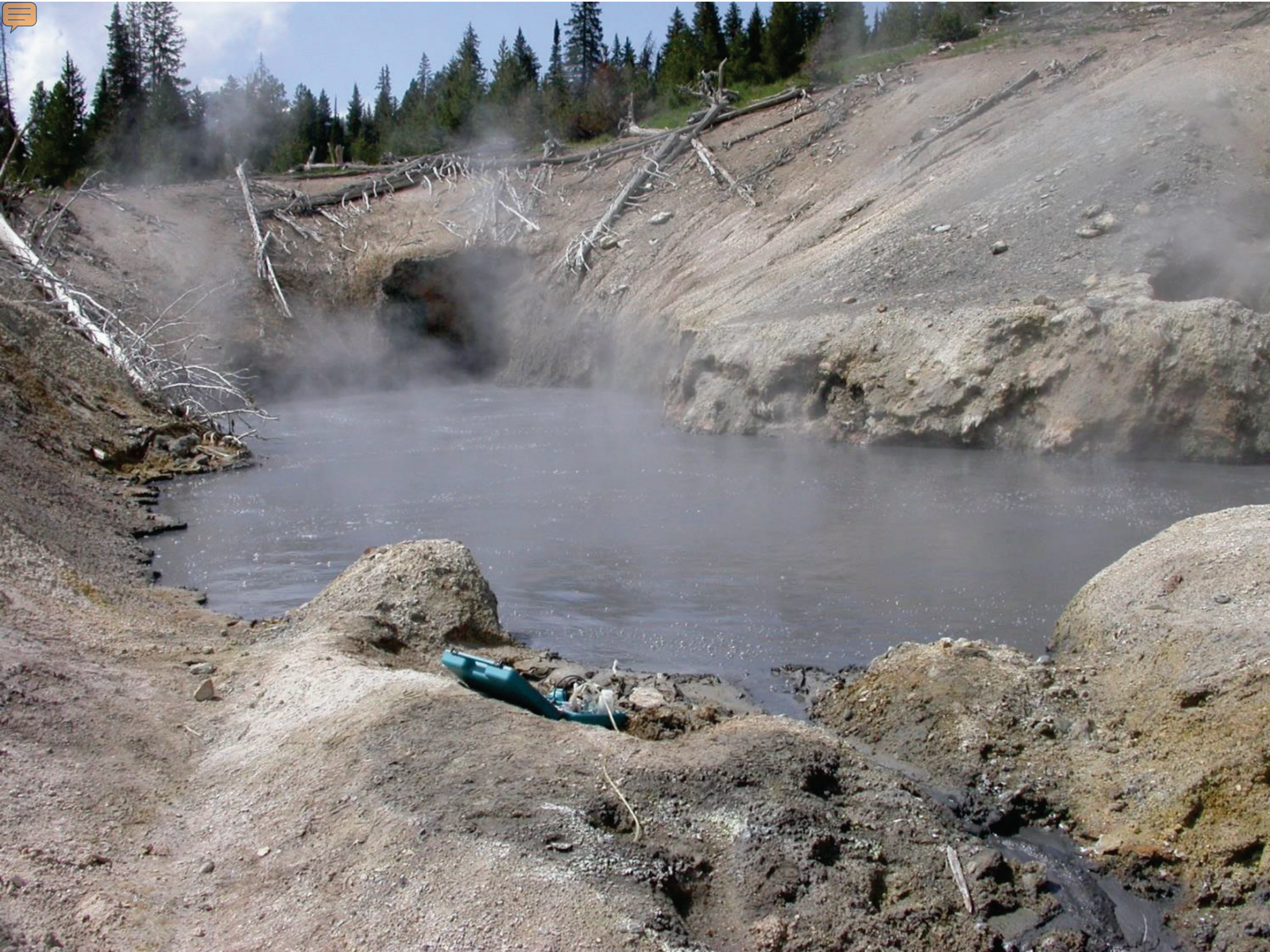

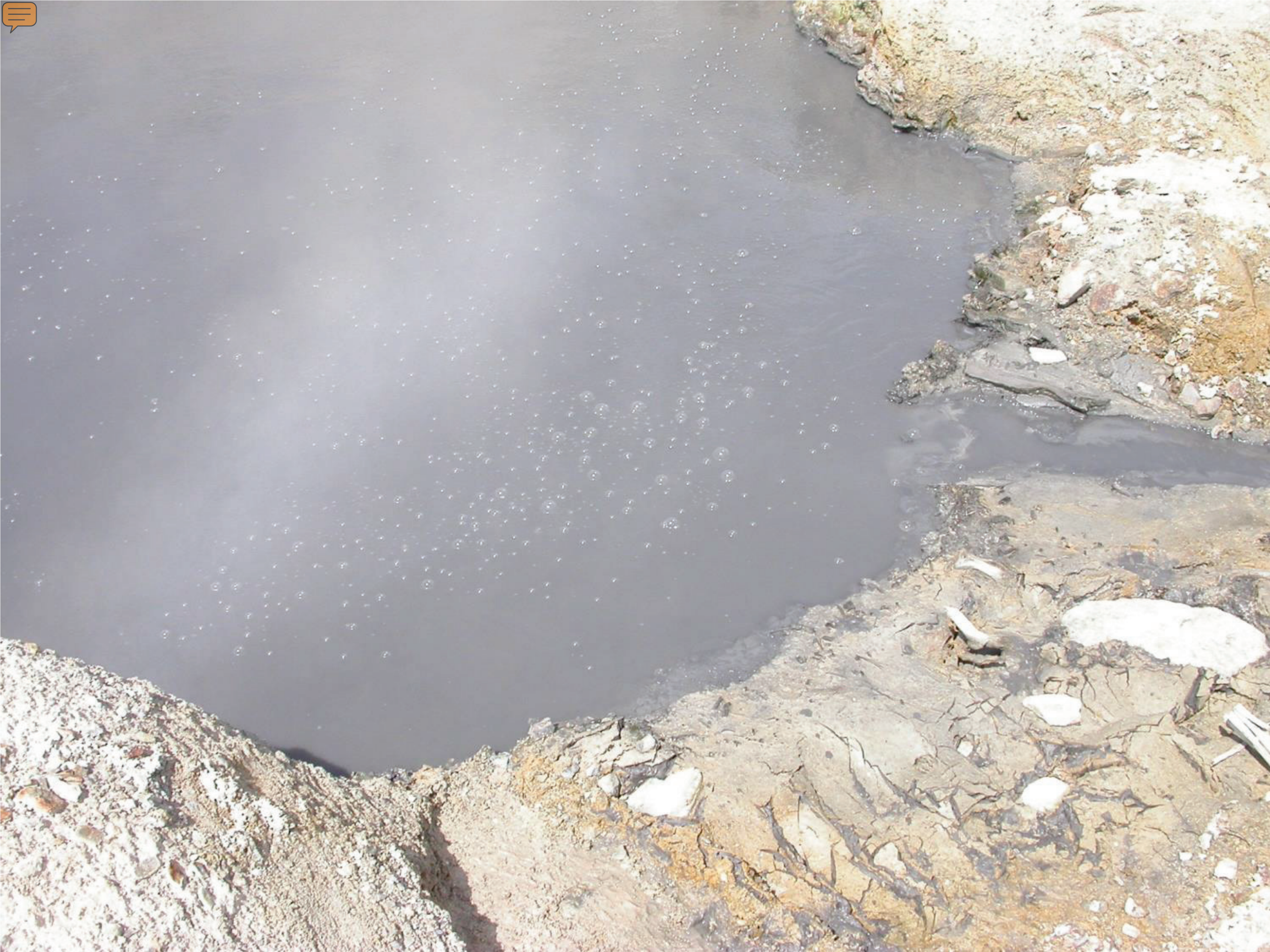

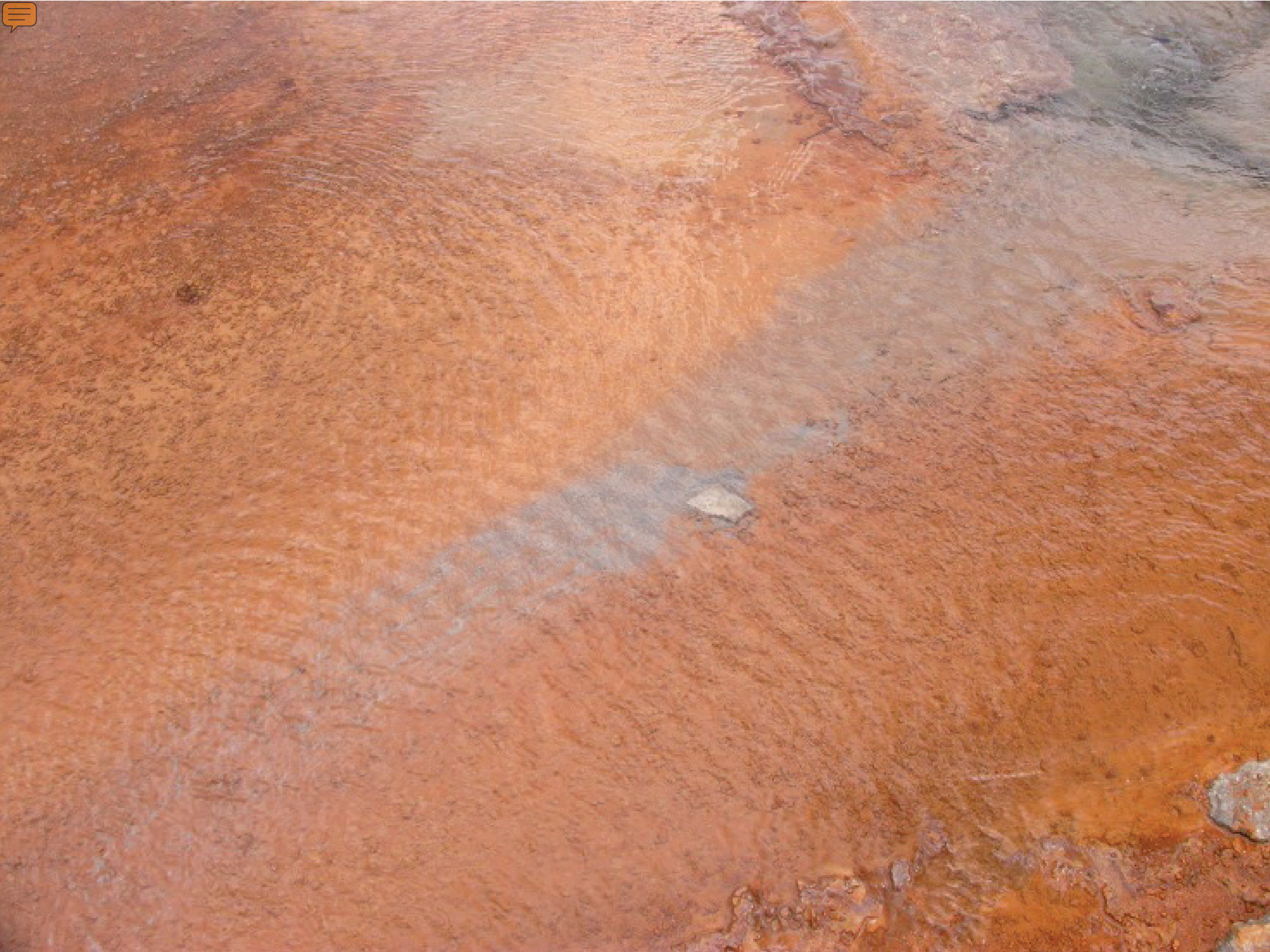

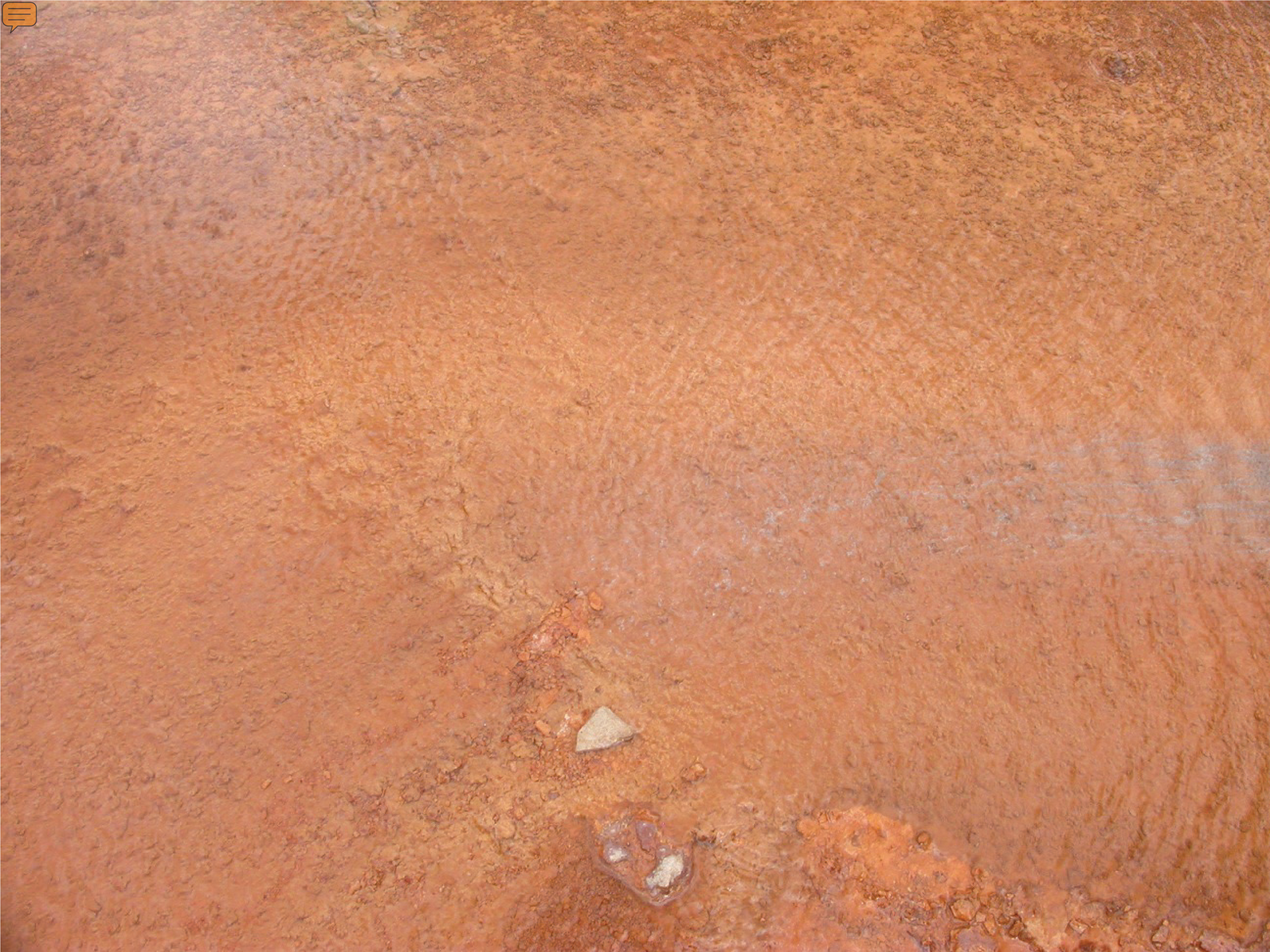

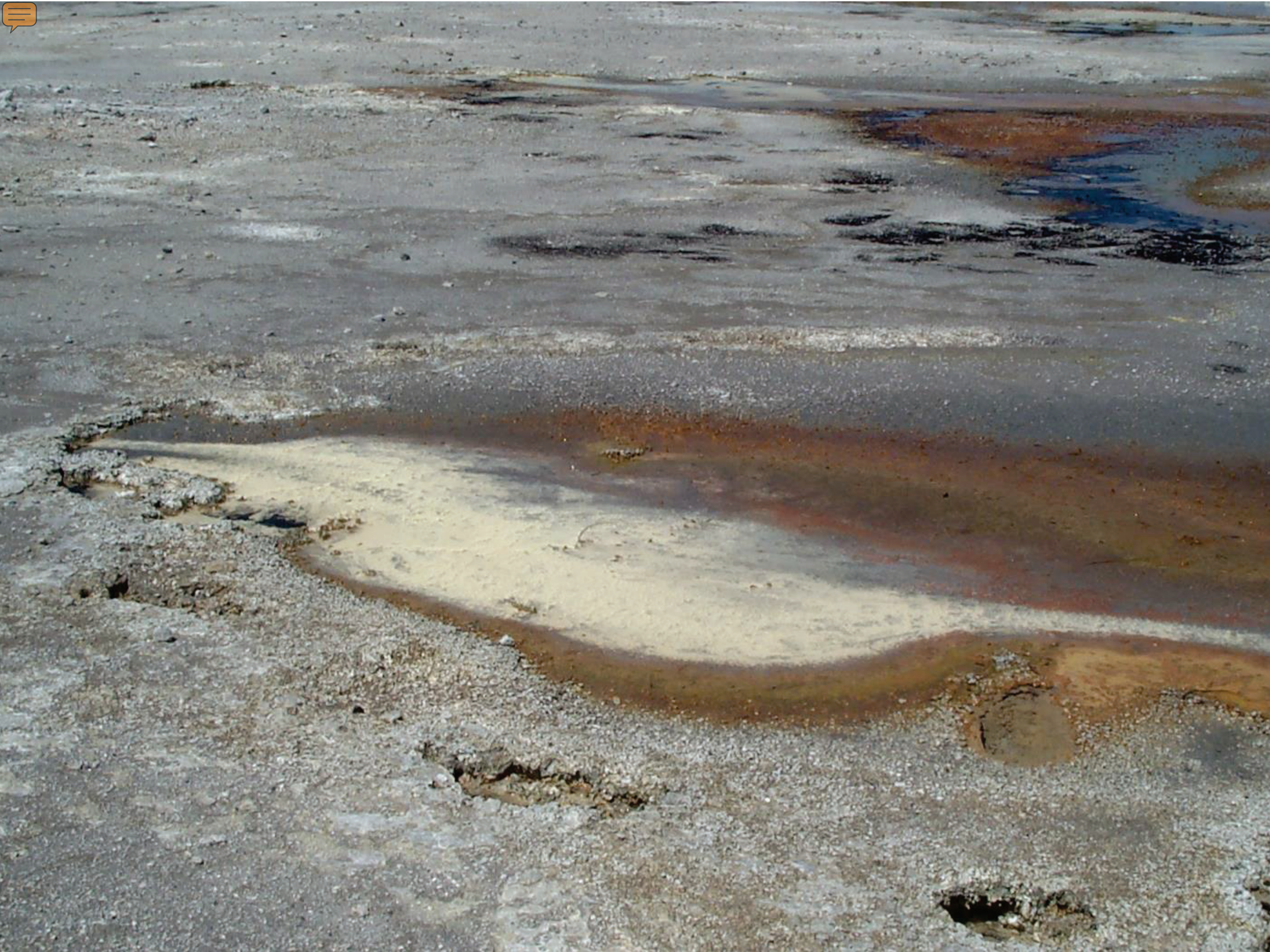

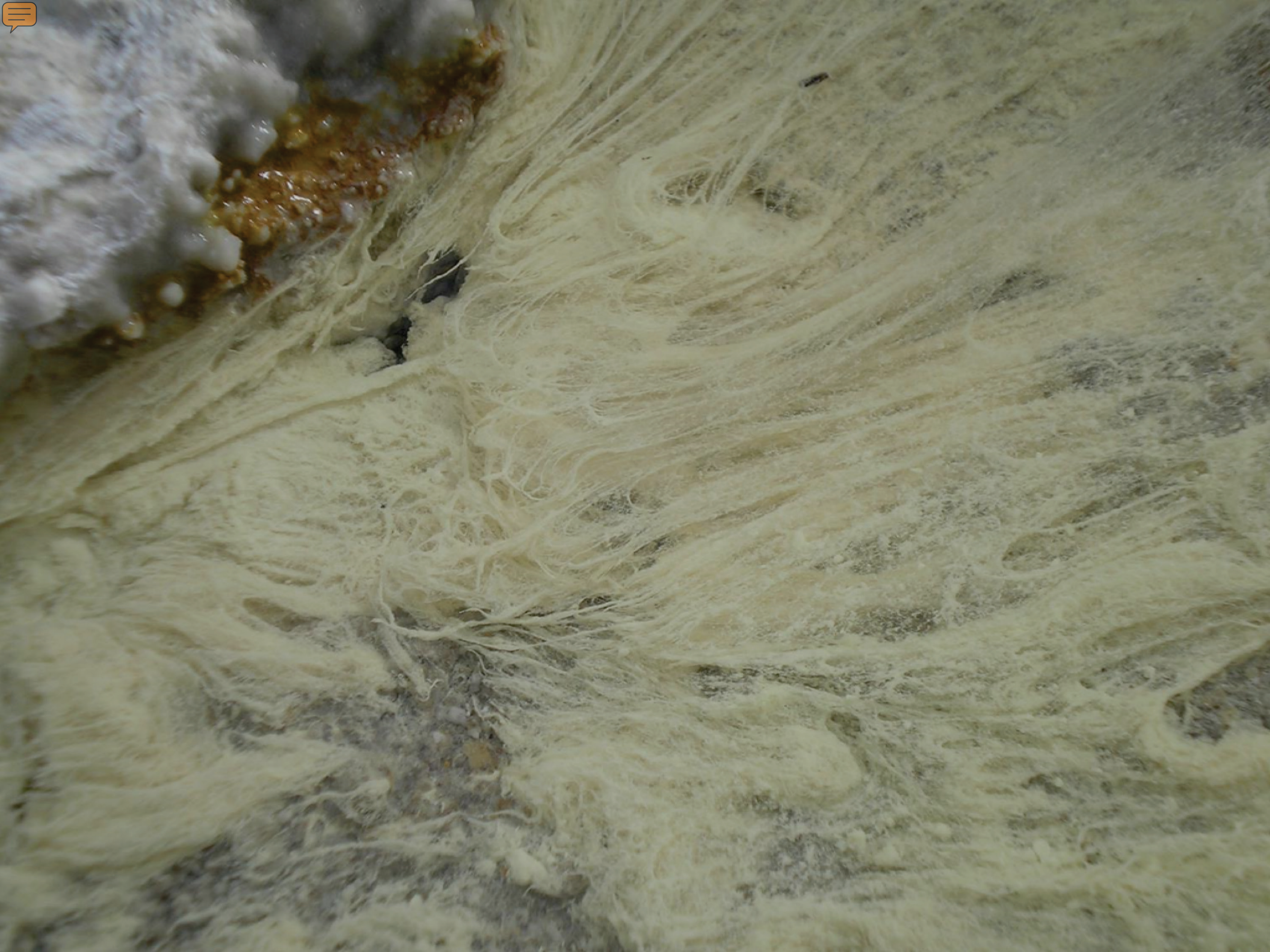

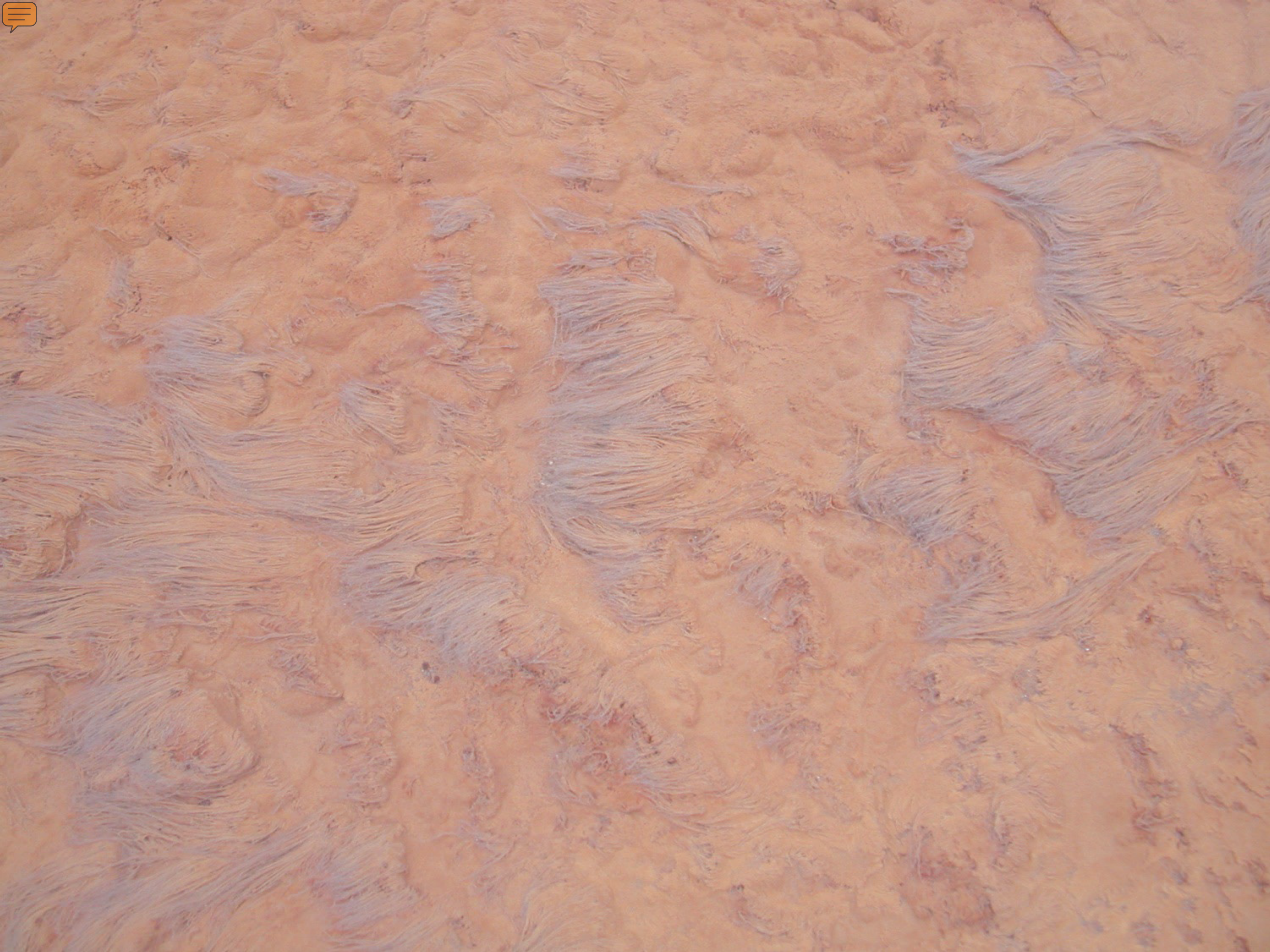

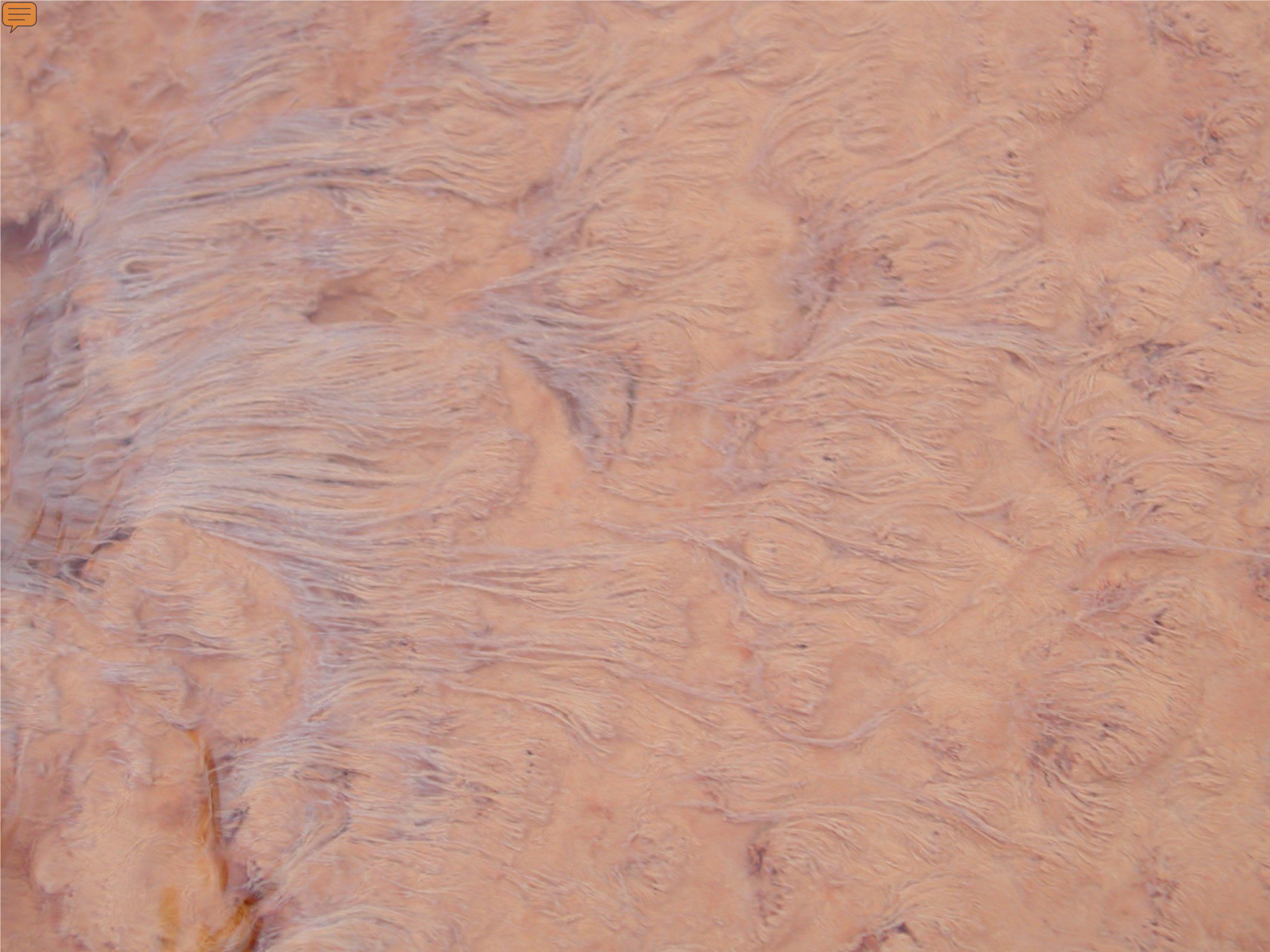

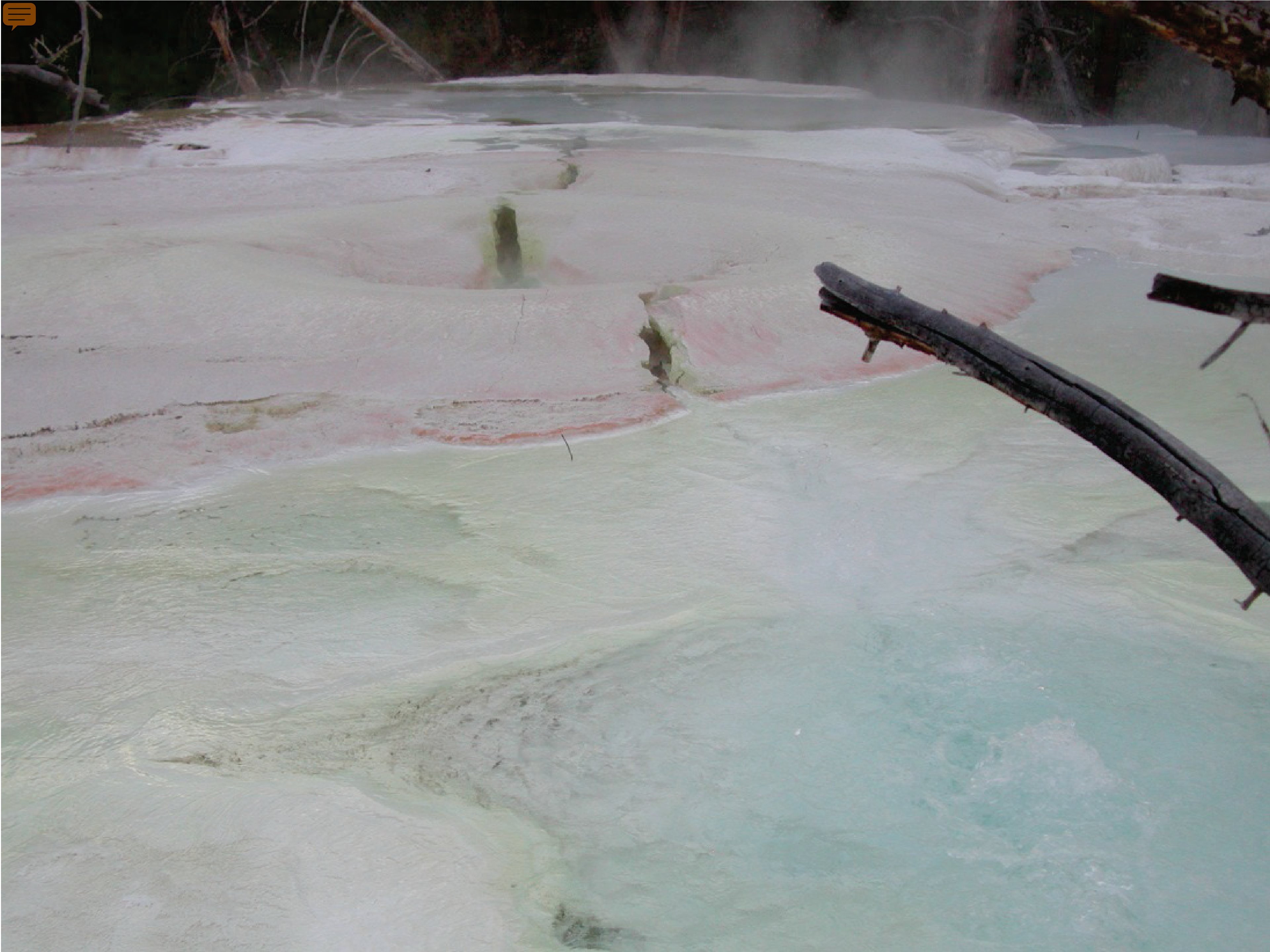

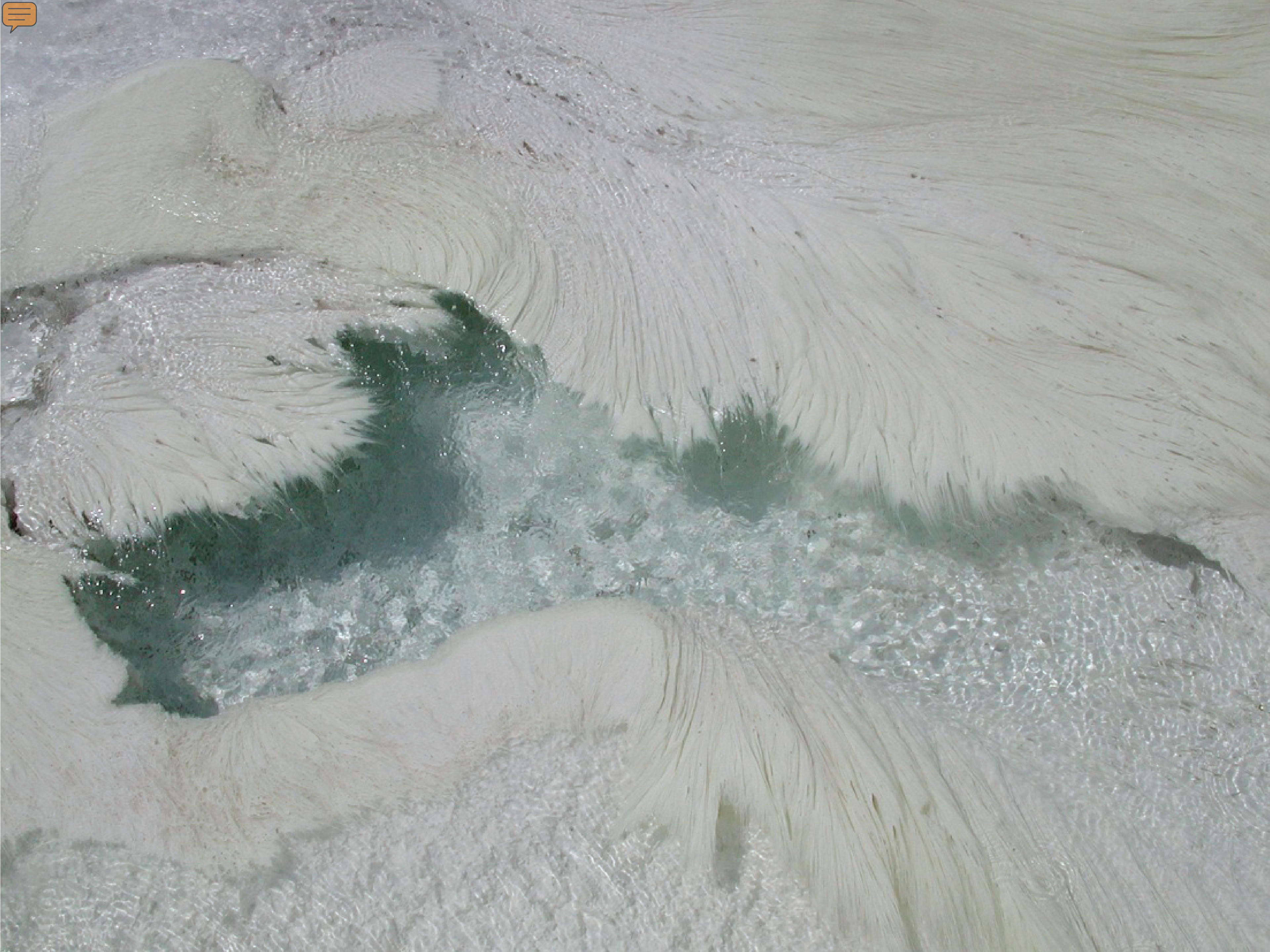

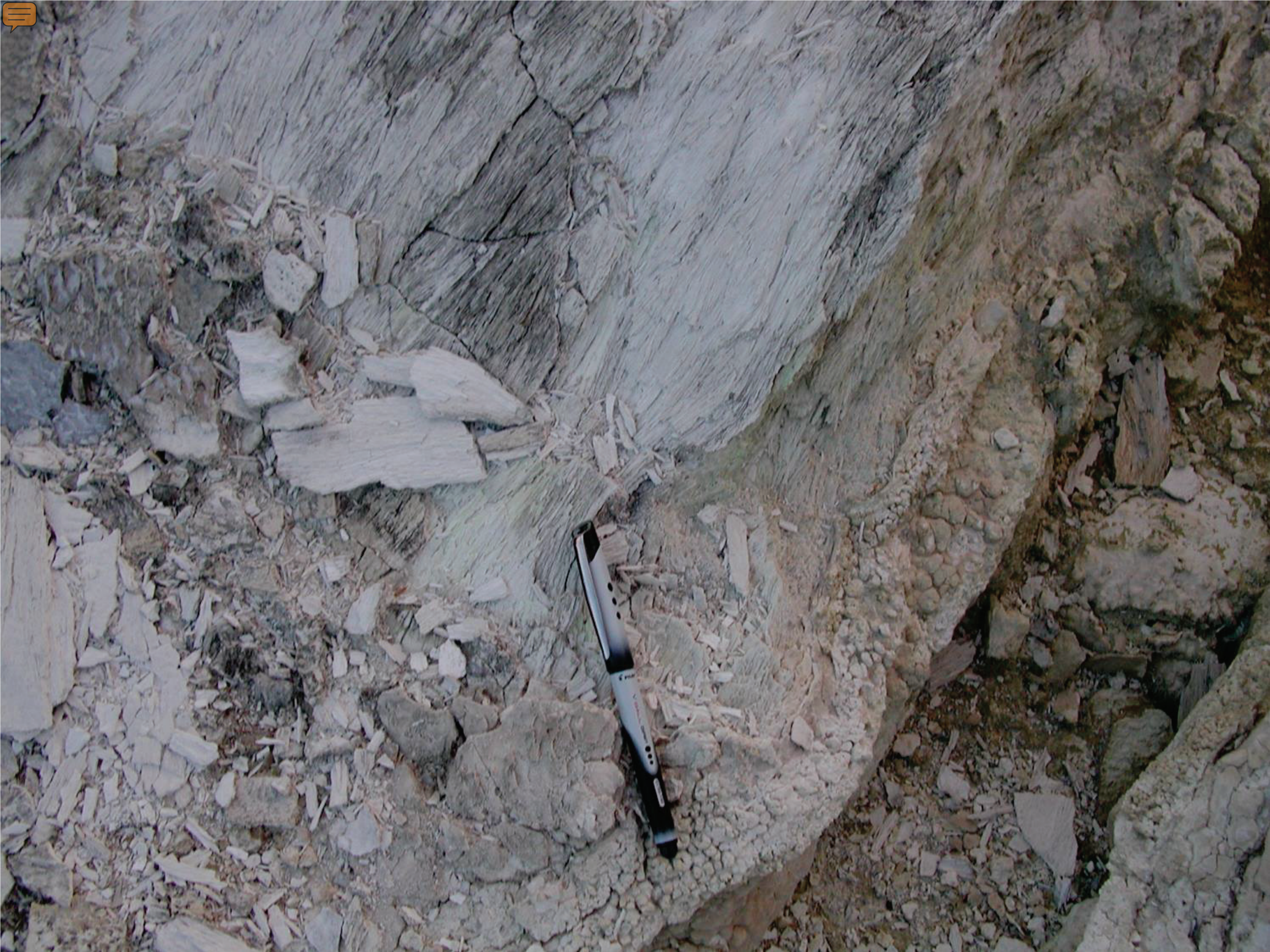

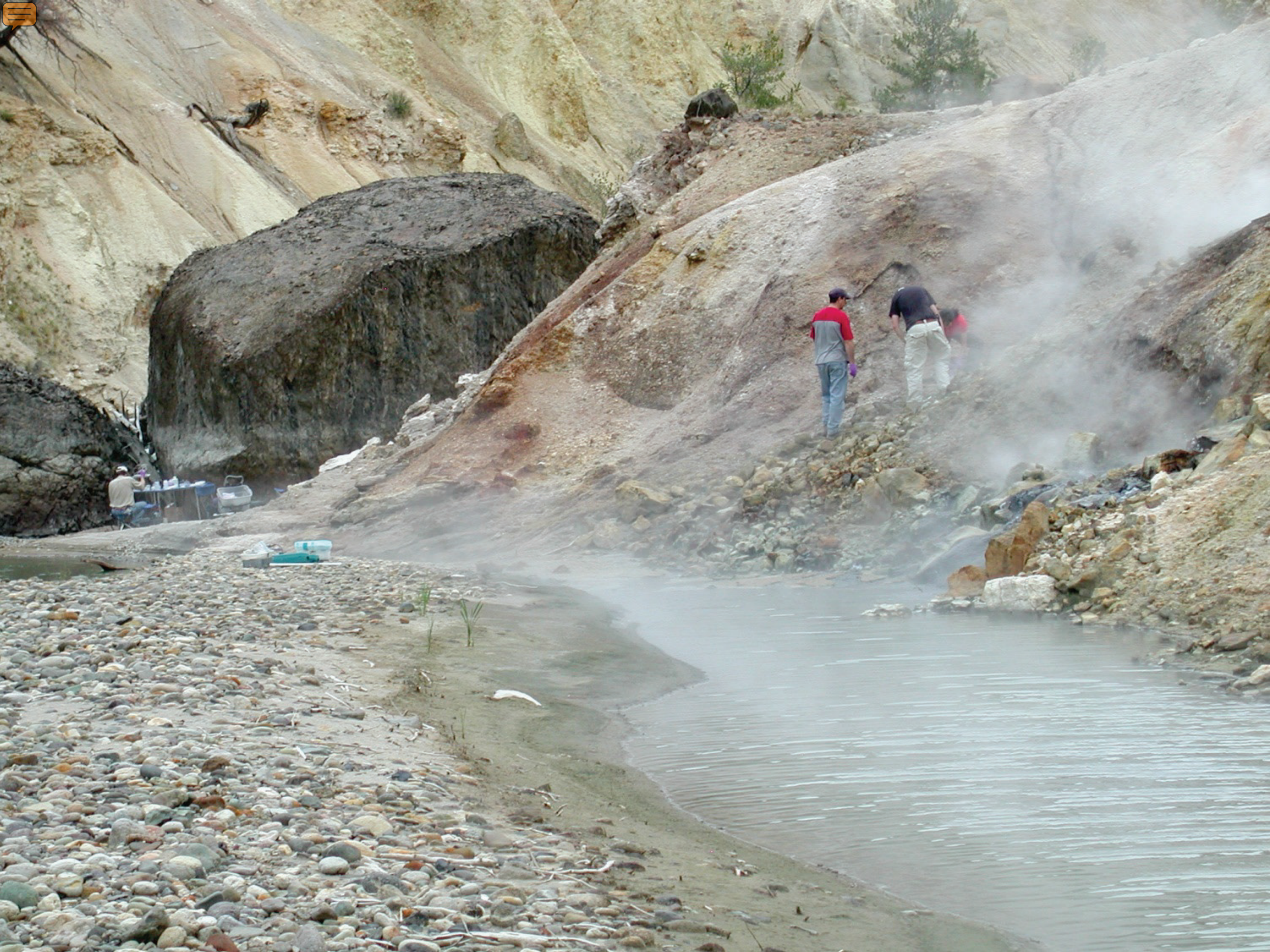

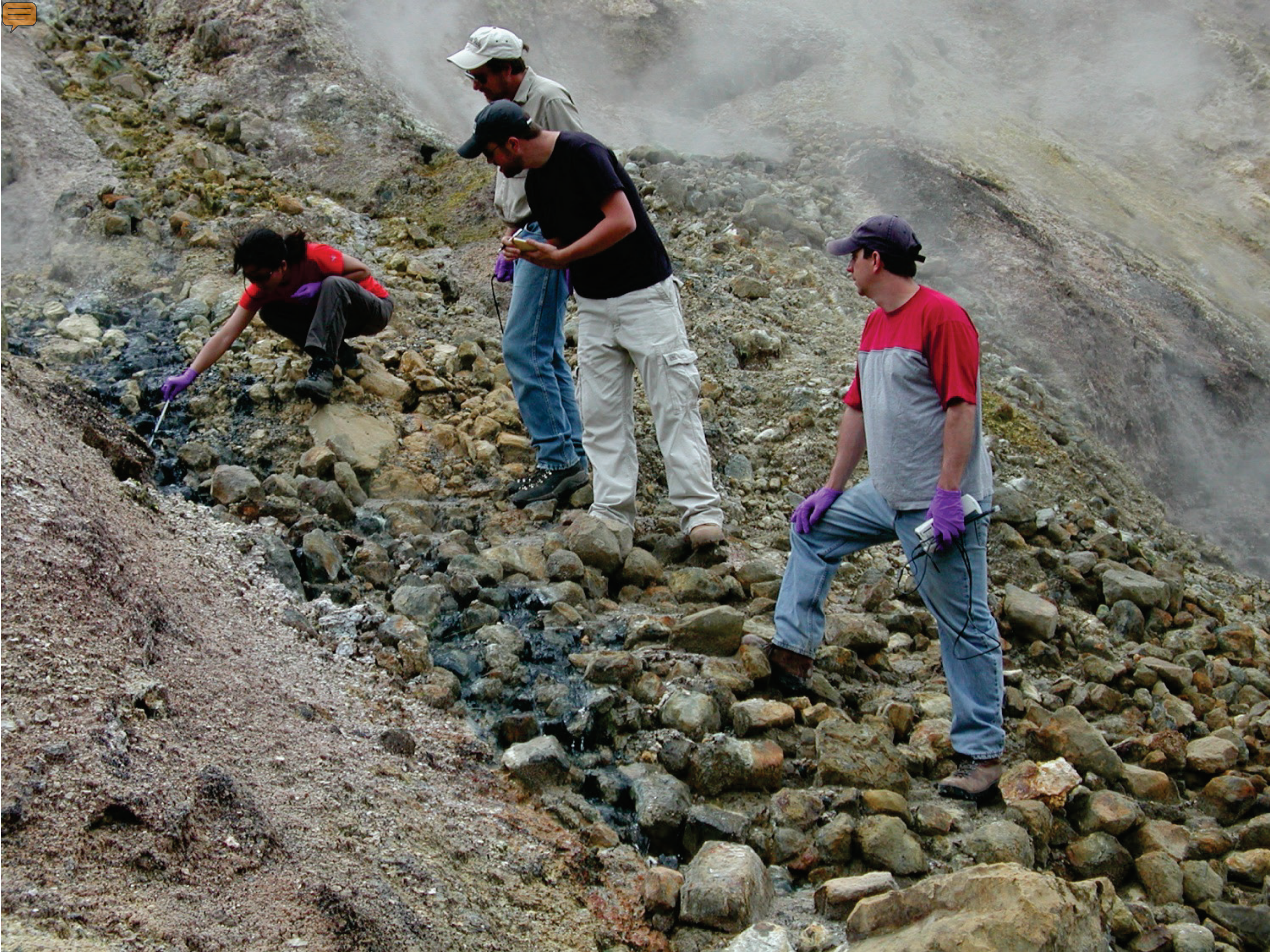

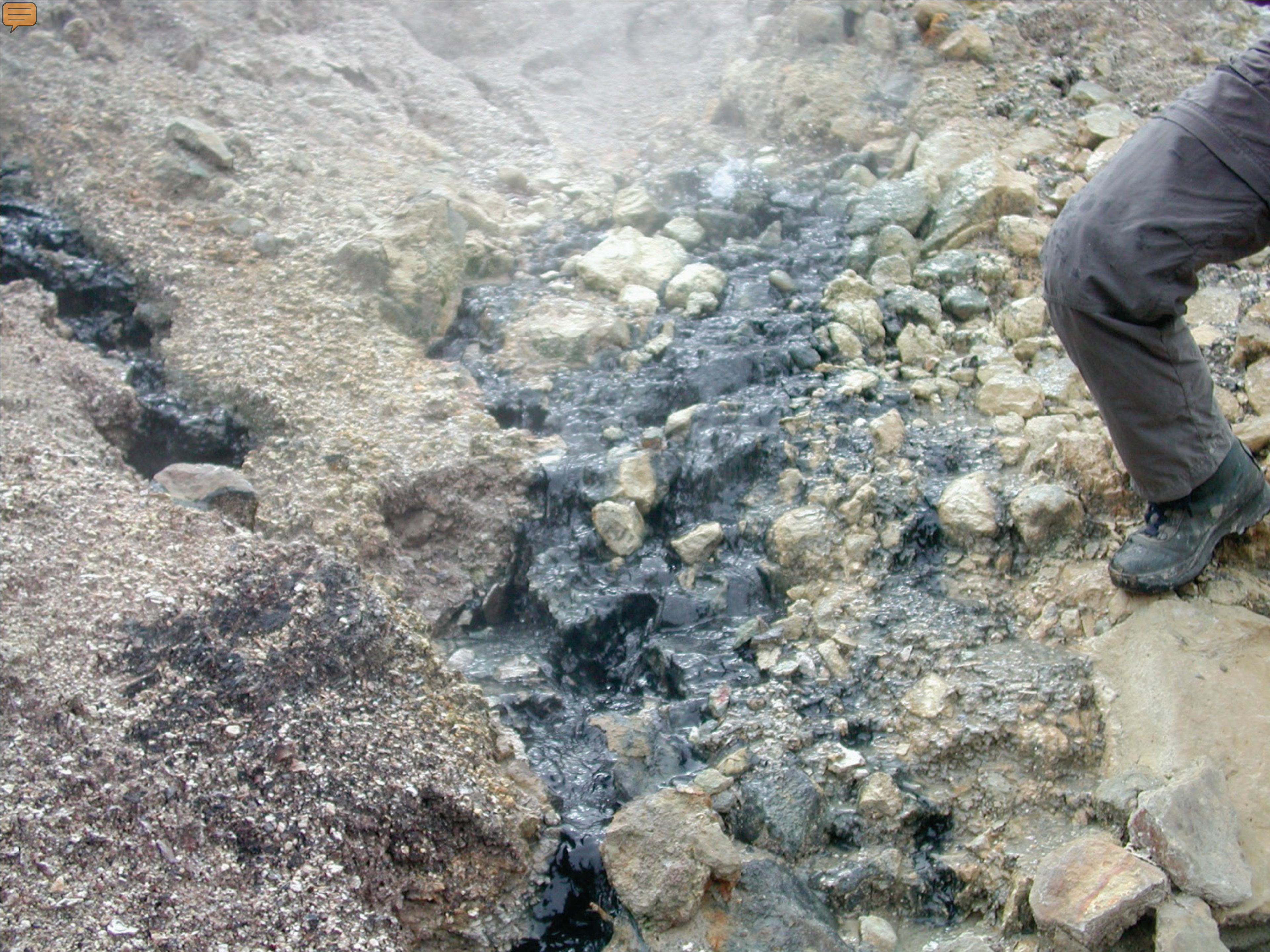

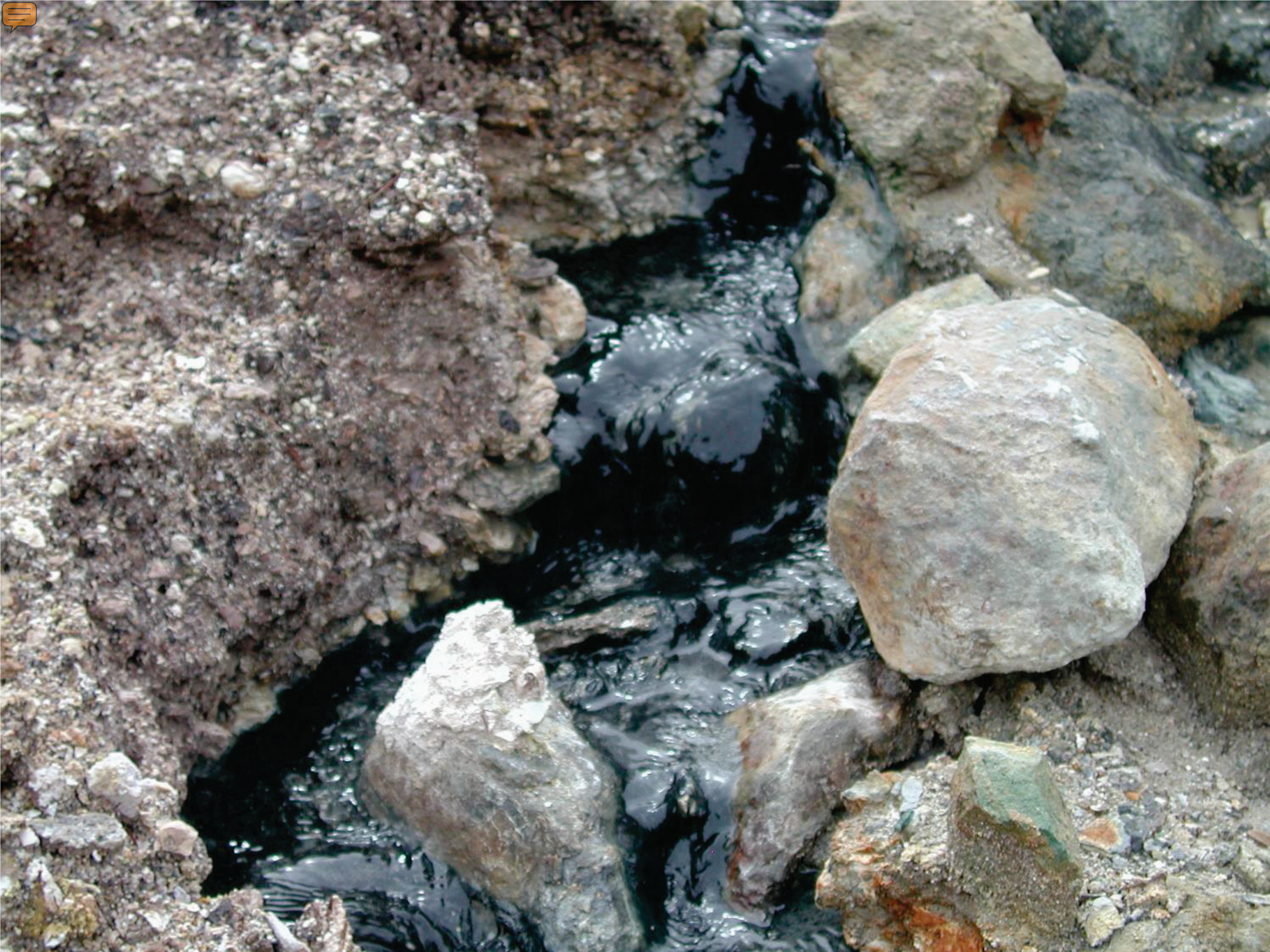

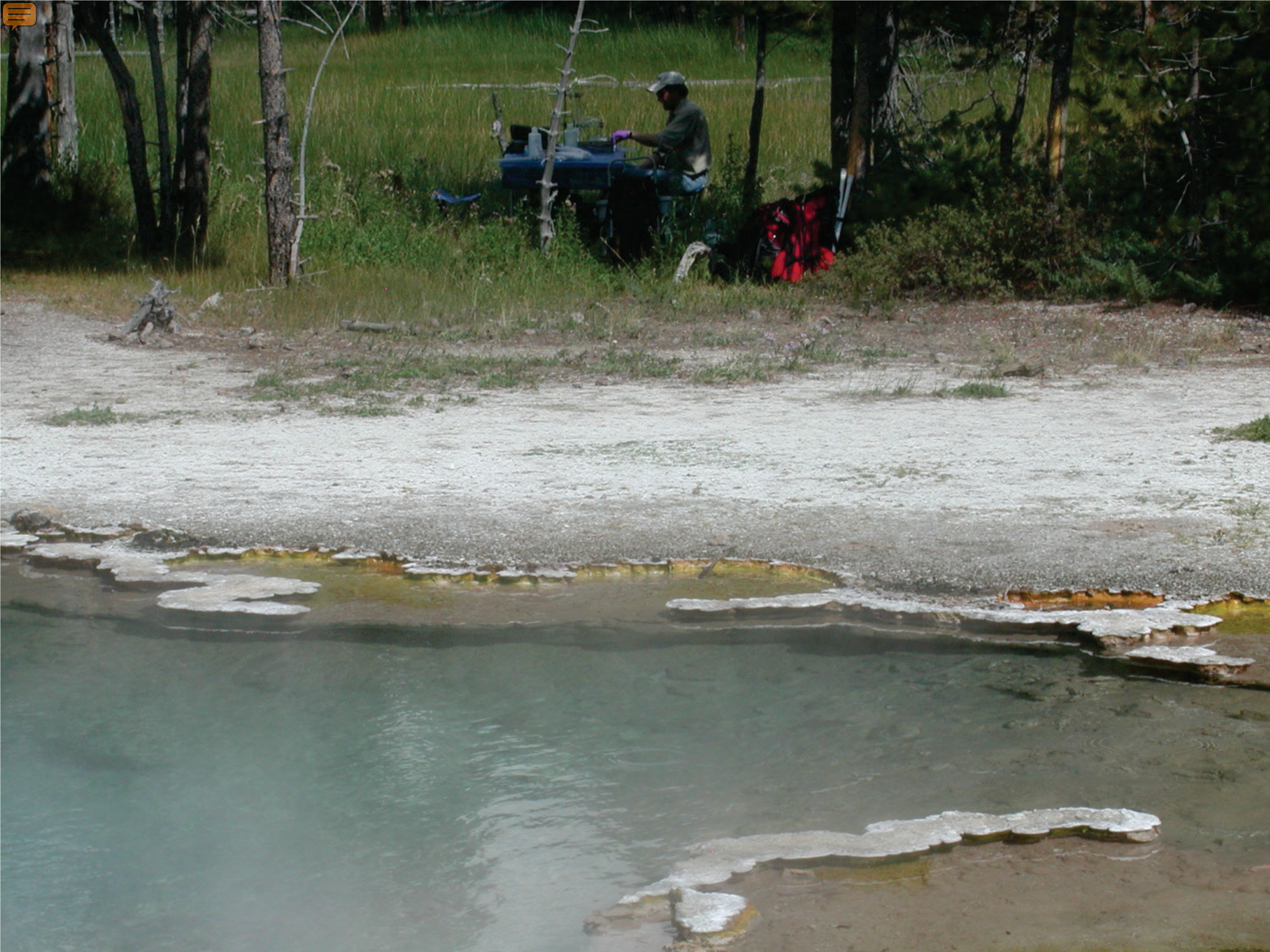

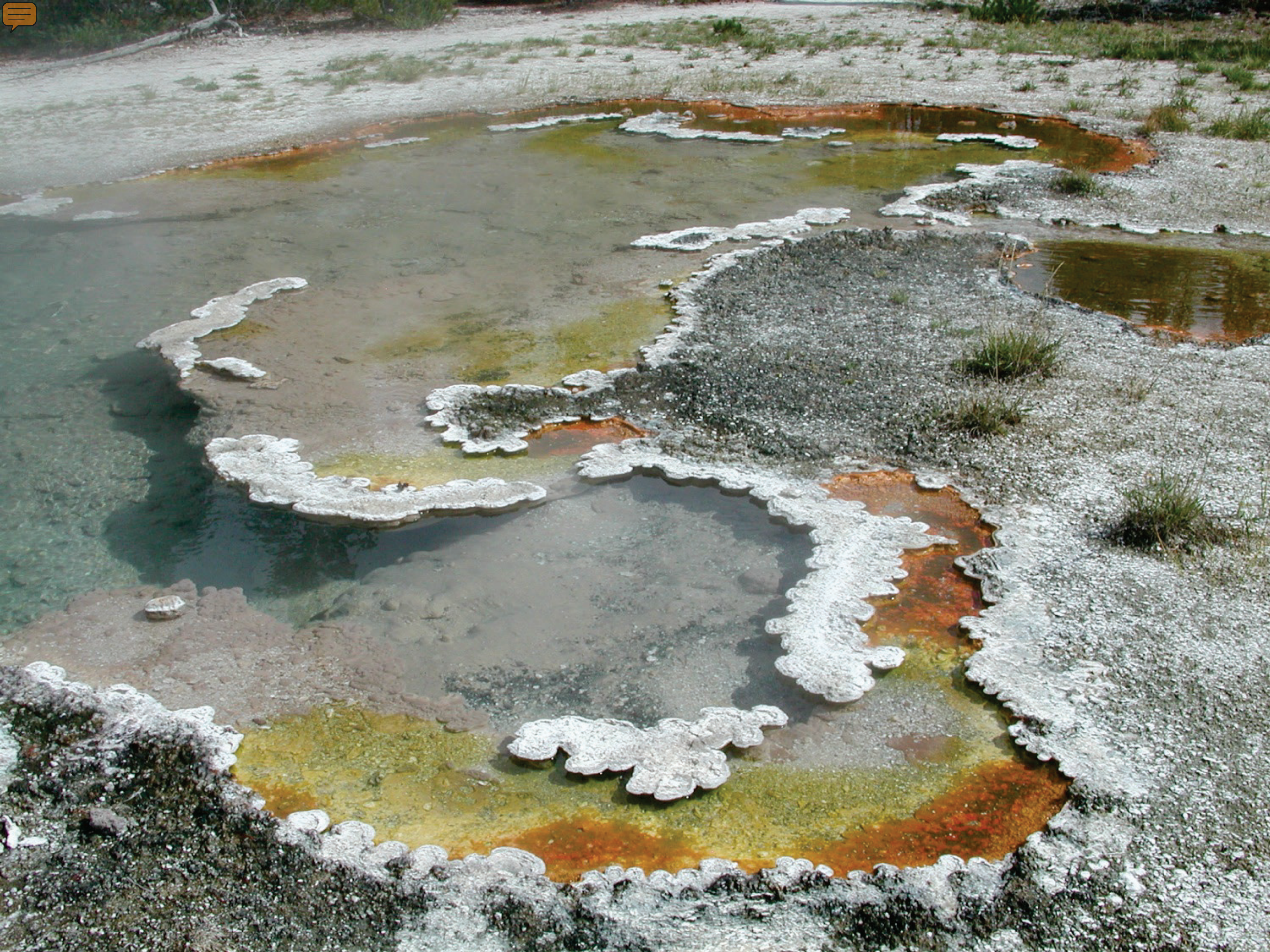

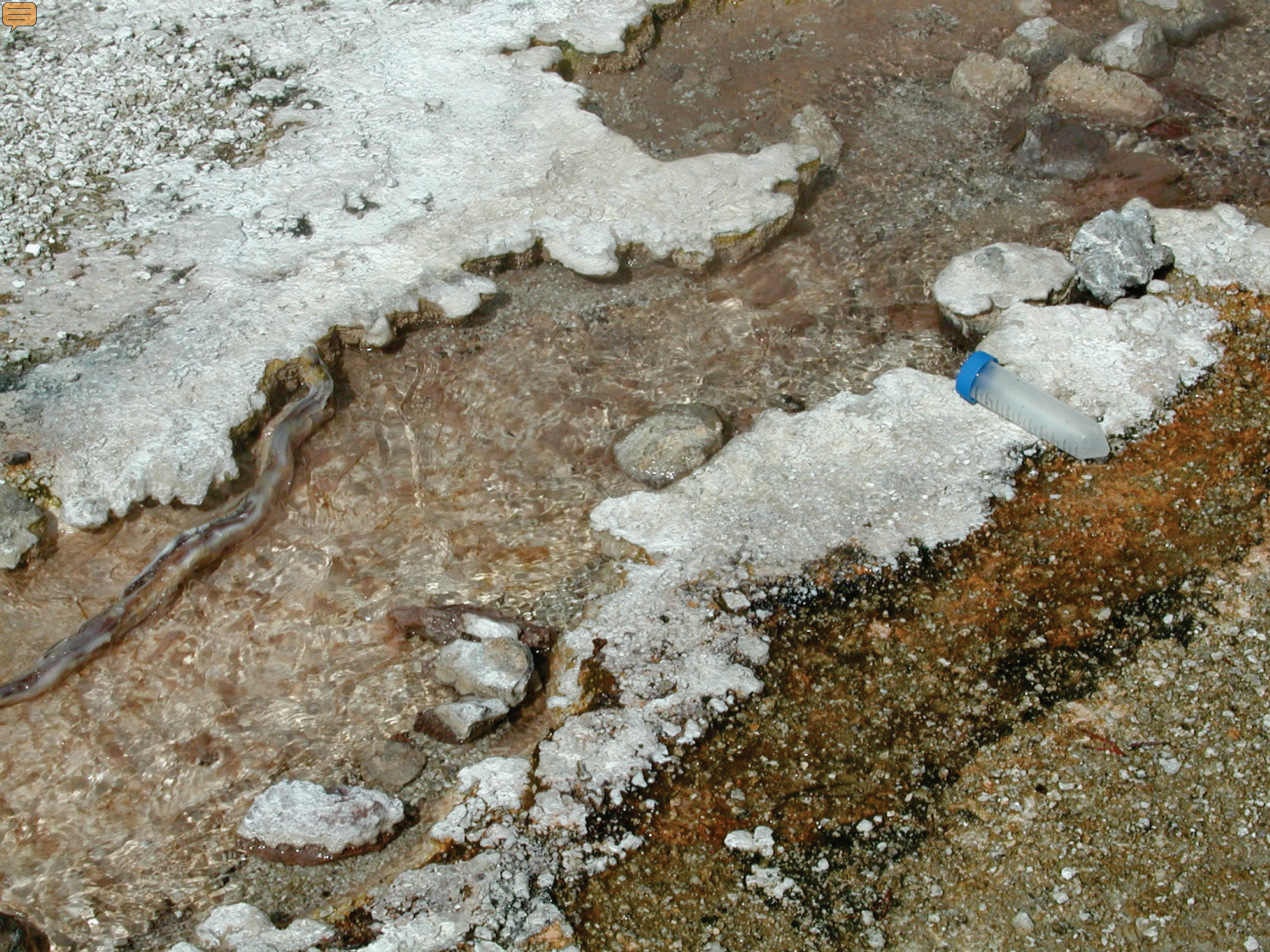

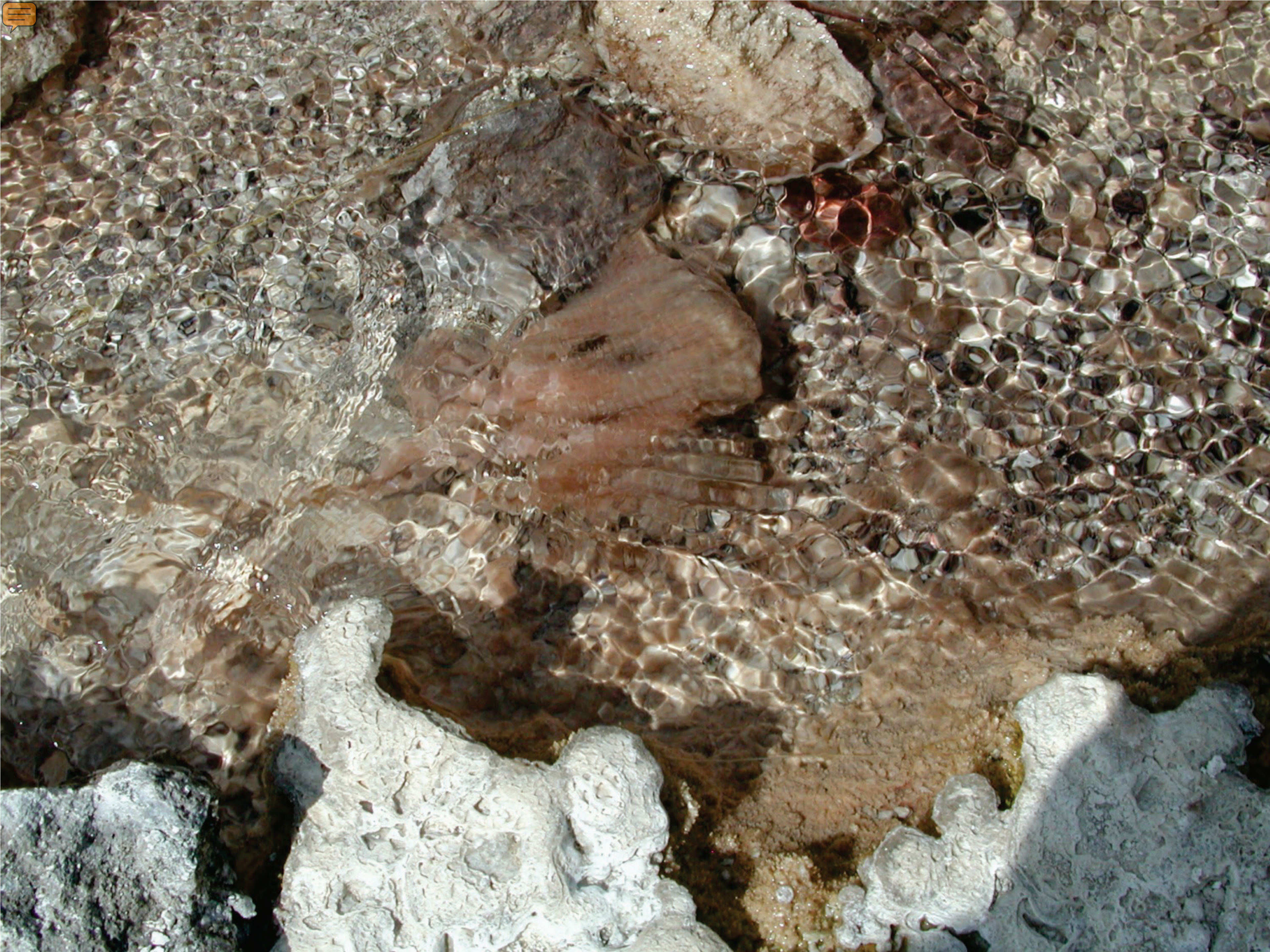

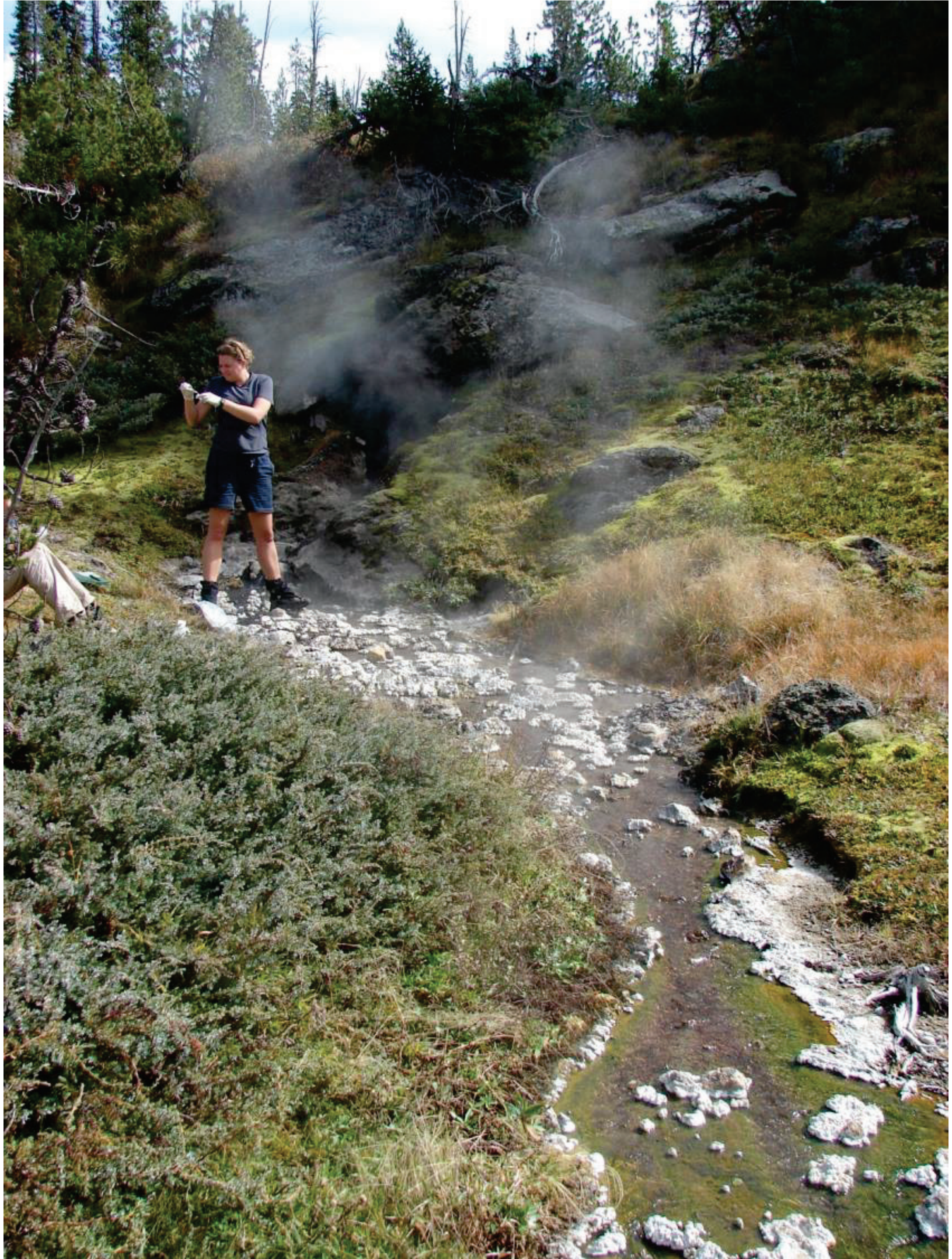

Supplement: Supplementary Figure S1 — Additional site photographs emphasizing landscape context of geothermal habitats and field sampling efforts (included as a separate file containing 53 annotated photographs). [file 41697_Inskeep_Presentation1.ZIP › 41697_Inskeep_Figure_S1.pdf]
