# Supplementary figures and images for: The YNP Metagenome Project: Environmental Parameters Responsible for Microbial Distribution in the Yellowstone Geothermal Ecosystem
Source: Front Microbiol. 2013 May 6;4:67. doi: 10.3389/fmicb.2013.00067 (PMC3644721; doi:10.3389/fmicb.2013.00067)

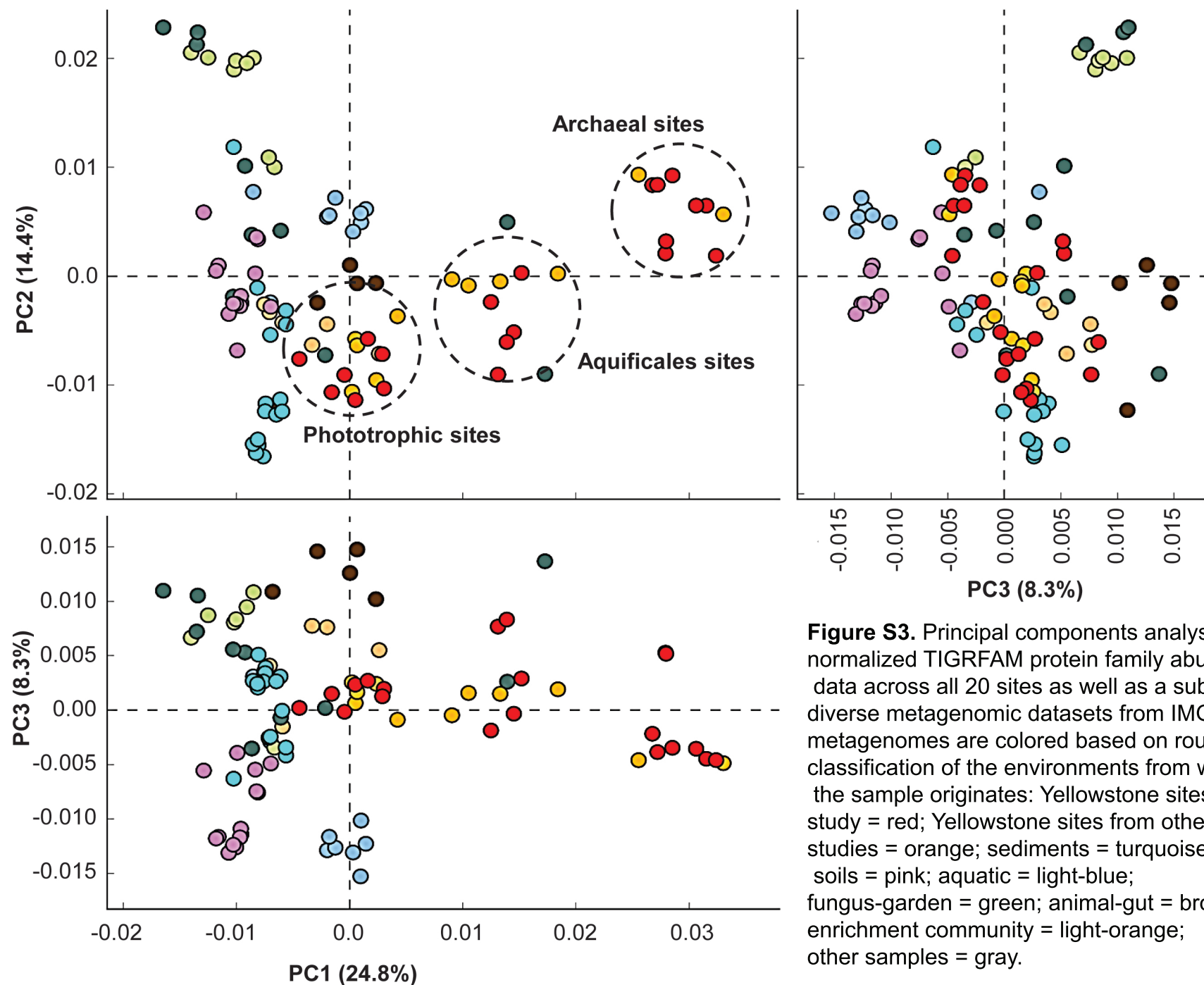

Supplement: Supplementary Figure S1 — Additional site photographs emphasizing landscape context of geothermal habitats and field sampling efforts (included as a separate file containing 53 annotated photographs). [file 41697_Inskeep_Presentation1.ZIP › 41697_Inskeep_Figure_S3.pdf]
